# The molecular mechanisms of cardiac development and related diseases

**DOI:** 10.1038/s41392-024-02069-8

**Published:** 2024-12-23

**Authors:** Yingrui Li, Jianlin Du, Songbai Deng, Bin Liu, Xiaodong Jing, Yuling Yan, Yajie Liu, Jing Wang, Xiaobo Zhou, Qiang She

**Affiliations:** 1 Department of Cardiology, The Second Affiliated Hospital of Chongqing Medical University, Chongqing, China; 2 Department of Cardiology, Angiology, Haemostaseology, and Medical Intensive Care, Medical Centre Mannheim, Medical Faculty Mannheim, Heidelberg University, Germany; DZHK (German Center for Cardiovascular Research), Partner Site, Heidelberg-Mannheim, Mannheim, Germany

**Keywords:** Cardiology

## Abstract

Cardiac development is a complex and intricate process involving numerous molecular signals and pathways. Researchers have explored cardiac development through a long journey, starting with early studies observing morphological changes and progressing to the exploration of molecular mechanisms using various molecular biology methods. Currently, advancements in stem cell technology and sequencing technology, such as the generation of human pluripotent stem cells and cardiac organoids, multi-omics sequencing, and artificial intelligence (AI) technology, have enabled researchers to understand the molecular mechanisms of cardiac development better. Many molecular signals regulate cardiac development, including various growth and transcription factors and signaling pathways, such as WNT signaling, retinoic acid signaling, and Notch signaling pathways. In addition, cilia, the extracellular matrix, epigenetic modifications, and hypoxia conditions also play important roles in cardiac development. These factors play crucial roles at one or even multiple stages of cardiac development. Recent studies have also identified roles for autophagy, metabolic transition, and macrophages in cardiac development. Deficiencies or abnormal expression of these factors can lead to various types of cardiac development abnormalities. Nowadays, congenital heart disease (CHD) management requires lifelong care, primarily involving surgical and pharmacological treatments. Advances in surgical techniques and the development of clinical genetic testing have enabled earlier diagnosis and treatment of CHD. However, these technologies still have significant limitations. The development of new technologies, such as sequencing and AI technologies, will help us better understand the molecular mechanisms of cardiac development and promote earlier prevention and treatment of CHD in the future.

## Introduction

The heart, as the first functional organ in the fetus, undergoes a complex developmental process starting from the differentiation of mesodermal cells during gastrulation. This process involves three main regions with cardiac precursor cells, namely, the cardiogenic mesoderm, the proepicardium, and the cardiac neural crest. Through the coordinated regulation of various signaling pathways, heart development begins with the migration of mesodermal cells from the primitive streak, moving to the both sides of the embryonic midline formatting two heart-forming regions (HFRs), each consisting of the first heart field (FHF) on the anterior lateral side and the secondary heart field (SHF) on the anterior medial side. The HFRs then merge to form the cardiac crescent, from which cells of the FHF eventually fold to form the primitive heart tube, initiating subsequent pulsation. Subsequently, the heart tube undergoes rapid growth through the recruitment of cells from the SHF and cell proliferation. Simultaneously, it bends to the right and initiates looping, ultimately leading to the formation of the right and left ventricles and atria, accompanied by the development of myocardial trabeculae, growth of the epicardium, development of the coronary vascular system, and subsequent formation of cardiac septa and valves.^1–3^

Based on previous studies exploring cardiac development, numerous signaling pathways, particularly those involving various transcription factors (TFs) and growth factors (GFs), have been found to play pivotal roles in different phases of heart development.^4–6^ For instance, the transcription factor NK2 homeobox 5 (NKX2-5) is a marker of cardiac precursor cells that regulates the proliferation and differentiation of these cells in the early phases of cardiac development.^7^ Another important transcription factor for cardiac development, GATA binding protein 4 (GATA4), has been found to interact with NKX2-5 through its zinc finger structure and specific residues in the C-terminal extension,^8^ while bone morphogenetic protein (BMP) 4, one of the GFs essential for embryonic heart development, has been suggested to regulate NKX2-5 expression via GATA4.^9^ Therefore, the molecular mechanisms of cardiac development are complex and intricate, involving numerous molecules and pathways in different cells that interact to form a finely tuned-regulatory network, thereby promoting normal cardiac development. Over the last few decades, advancements in genetics, molecular biology, and cell biology have deepened our understanding of the mechanisms of cardiac development. The development of stem cell and organoid technology allows us to mimic the process of animal or human cardiac development in vitro, although this technology still faces many challenges, such as the immaturity of differentiated cells and differences from the in vivo environment. Nevertheless, these studies provide a possible in vitro model for further understanding and validating the molecular mechanisms of cardiac development.

Multi-omics analysis, stemming from the development of bioinformatics, also provides technical support for understanding the process of cardiac development at different levels. A study by Hu et al. identified novel core TFs [Kruppel-like factor 11 (KLF11) and E2F transcription factor 6 (E2F6)] and dynamic changes in enriched key pathways through multi-omics analysis.^10^ Another study delineated cellular niches within eight regions of the human heart, revealing the characteristics of cells in the cardiac conduction system and their multicellular niches.^11^ These new technologies enable us to understand the molecular mechanisms of cardiac development at different cellular and regulatory levels, comprehensively improving our knowledge of cardiac development.

Disruption of the molecular regulatory network of cardiac development by genetic or nongenetic factors can lead to various types of cardiac malformations, which are characteristic of congenital heart defects (CHDs), with ventricular septal defects (VSDs) being the most common. CHDs encompass various types, and depending on the location of the cardiac malformation, they can be classified as septal defects, conotruncal defects, valve defects, among others. Several critical CHDs, including hypoplastic left heart syndrome (HLHS), transposition of the great arteries, and pulmonary atresia, can result in severe consequences if not treated promptly. Moreover, certain developmental diseases affecting multiple organ systems are often associated with a high incidence of CHD, such as DiGeorge syndrome, commonly linked with Tetralogy of Fallot (ToF),^12^ and Noonan syndrome, often associated with pulmonary valve stenosis (PVS).^13^ These different types of CHDs have distinct pathogenic mechanisms. For instance, mutations in TBX5 frequently lead to septal defects, while abnormalities in Notch and TGFβ signaling are often associated with defective valve development. However, the pathogenesis of CHD remains unclear, with only 15% of cases attributed to genetic inheritance and 30% associated with environmental risk factors, including fever, infections, maternal smoking, alcohol consumption, diabetes, and hypertension.^14^ These factors can affect any aspect of the cardiac developmental regulatory network, leading to abnormalities in the differentiation and proliferation of cardiomyocytes, endocardial cells, and smooth muscle cells and ultimately resulting in congenital heart defects.^14,15^ Although surgical procedures and palliative care are the main treatments for CHD, advancements in understanding cardiac development offer promising avenues for innovative therapeutic approaches, particularly in the early prevention and treatment of CHD. Advances in genetic technologies, bioinformatics, and big data analytics may enable the precise identification of genetic or nongenetic factors contributing to CHD, facilitating the development of precision and individualized disease prediction models and more effective prevention strategies, ultimately reducing the occurrence of CHD and the necessity for surgical interventions in the future.

This review aims to summarize the processes and molecular mechanisms involved in cardiac development, as well as the latest applied technologies and discoveries in this field, which will significantly enhance our understanding of cardiac development. Furthermore, we explore diseases associated with abnormal cardiac development and offer insights into future directions for managing CHD.

## The history of cardiac development research

**Fig. 1:**
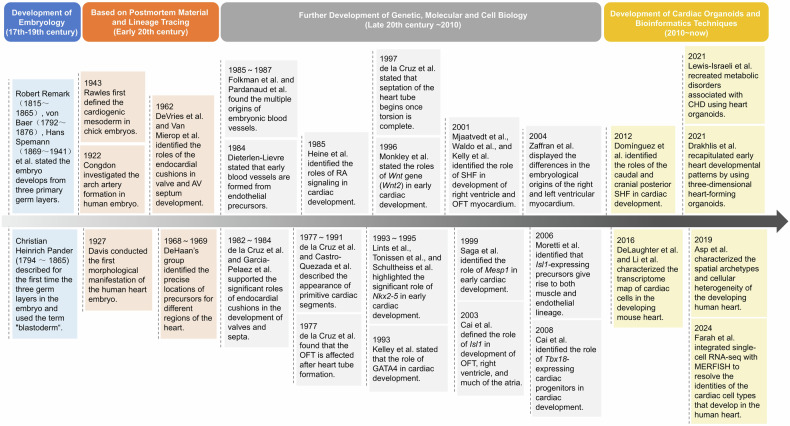
Timeline of milestone events in cardiac development research history. Each significant breakthrough in cardiac development research has been accompanied by advancements in related disciplines and technologies. The research of cardiac development starting with early studies observing morphological changes to exploration of molecular mechanisms using various molecular biology methods. Nowadays, advancements in stem cell technology and sequencing technology, such as the generation of cardiac organoids and multi-omics sequencing, have enabled researchers to understand the molecular mechanisms of cardiac development better. AV atrioventricular; AVC atrioventricular canal, CHD congenital heart disease, GATA4 GATA binding protein 4, hPSCs human pluripotent stem cells, Isl1 islet 1, MERFISH multiplexed error-robust fluorescence in situ hybridization, Mesp1 mesoderm posterior 1, Nkx2-5 NK2 homeobox 5, OFT outflow tract, PE proepicardium, RA retinoic acid, SHF second heart field, Tbx18 T-box transcription factor 18. This figure was created using Microsoft Powerpoint

The exploration of cardiac development has a long and intricate journey (Fig. 1). With the advent of microscopy at the end of the 17th century, embryology experienced rapid development. This progress was significantly influenced by William Harvey (1578–1667) through his observations of deer and chick embryos, Caspar Friedrich Wolff (1733-1794) with his theory of epigenesis, and Karl Ernst von Baer (1792–1876) with his formulation of von Baer’s laws. These foundational works paved the way for Robert Remak’s (1815–1865) theory of the three germ layers in embryonic development.^16,17^ In 1927, Davis conducted the first morphological study of human heart embryos using postmortem material. He proposed that the heart consists of different segments, each giving rise to a definitive cardiac cavity, which he termed “primitive cardiac cavities”—including the aortic bulb, bulbus cordis, left ventricle, and atria (right and left). Davis also described a V-shaped plate called the cardiogenic plate within the splanchnopleure and the appearance of the sinus venosus.^18,19^

In 1943, Rawles defined the cardiogenic mesoderm of the chick embryo as the region within the HH5 (Hamburger and Hamilton stage 5) lateral plate mesoderm with myocardial potential.^20^ With the advancement of molecular biology techniques, scientists have further explored cardiac development through lineage tracing and in vivo labeling. DeHaan’s group meticulously studied the morphological changes and fate map of the precardiac mesoderm in chicks. By transplanting radiolabeled donor embryos into stage HH5 chick embryos and comparing the initial and final positions of each transplant, they found that the morphogenesis of the heart likely occurs in a highly coordinated manner. This study identified the precise locations of precursors for different regions of the heart within these areas.^21–23^ Based on in vivo labeling and deletion experiments, researchers such as Castro-Quezada et al. ^24^ and de la Cruz and associates^25–27^ mapped the fate of the heart tube in chick embryos, indicating that primitive cardiac cavities do not directly form future definitive cardiac structures. Instead, new segments (primitive cardiac segments) appear during development from the heart tube to the early looping period, each contributing only to specific regions of the mature heart, differing from Davis’s conclusions. During this period, the complex regulatory mechanisms of cardiac development began to be understood. Researchers discovered that through the processes of torsion and looping of the cardiac tube, various primitive cardiac segments change their spatial positions and establish new relationships with each other, ultimately facilitating the normal septation of the heart.^28,29^

Moreover, significant findings have been made regarding the formation of the cardiac septum, valves, conduction system, and blood vessels.^18^ Endocardial cushions of the atrioventricular canal were found to play a crucial role in the formation of the cardiac septum and leaflets.^28,30–32^ Transplantation experiments suggested that embryonic blood vessels originate from multiple sources, forming and growing through vasculogenesis, angiogenesis, and the incorporation of local and wandering angioblasts.^33–37^ The roles of various molecules and signals in heart development have become increasingly recognized, including retinoic acid (RA);^38–40^ TFs such as NKX2-5,^41^ GATAs,^42,43^ and myocyte enhancer factor 2 (MEF2);^44^ and GFs such as fibroblast growth factor (FGF),^45^ transforming growth factor (TGF)-β,^46^ BMP-2.^47^ Other important factors involved in heart looping include flectin,^48,49^ heart and neural crest derivatives expressed (HAND)1, and HAND2.^50^ These discoveries have deepened our understanding of cardiac development, highlighting the complex, organized regulatory networks involved.

In 1977, de la Cruz and colleagues discovered that the outflow tract (OFT) is affected after heart tube formation, but the origins of these secondarily added cells remain uncertain.^28^ In 2001, several groups found that the OFT in chick hearts (HH18-22) is formed by mesodermal cells outside the classical heart-forming region, and in mice (from E8.25 to E10.5), the right ventricle (RV) and OFT myocardium are added from the pharyngeal arch core and splanchnic mesoderm, termed the SHF.^51–53^ Subsequent research has focused on SHF markers, functions, and regulation, with SHF now thought to reside in the pharyngeal mesoderm, contributing differently to the arterial and venous poles of the heart. Precursor cells from the anterior SHF contribute to the myocardium of the RV and OFT, as do smooth muscle cells associated with the great arteries. Cells added to the venous pole contribute to the myocardium of the atria and atrial septum.^54–58^ Islet 1 (ISL1),^59^ FGF8,^60^ and sine oculis homeobox homolog 2 (SIX2) have been identified as SHF markers,^57^ although some of these markers are nonspecifically expressed in other embryonic regions, including the FHF region.^61,62^ With the progress of technology and our deepened understanding of heart development, more detailed molecular regulatory mechanisms, such as the roles of epigenetic regulation and cilia in cardiac development, are being studied. These issues will be elaborated upon in the next chapter.

**Fig. 2:**
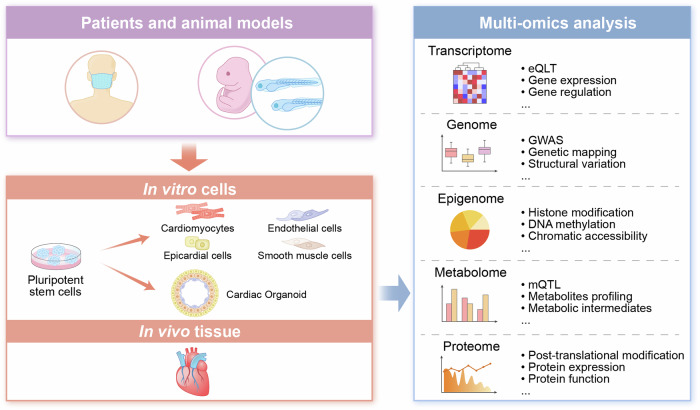
The molecular mechanisms of cardiac development were explored using pluripotent stem cell technology and multi-omics analysis. Pluripotent stem cells are generated by reprogramming adult somatic cells from CHD patients or animal models into a pluripotent state. These cells can differentiate into various cardiac cells, such as cardiomyocytes, smooth muscle cells, endothelial cells, and epicardial cells, through different differentiation protocols. Pluripotent stem cells can also form cardiac organoids through self-organization and specific differentiation methods, thereby creating an in vitro model of cardiac development. Multiomics analysis encompasses genomics, transcriptomics, proteomics, epigenomics, and metabolomics. By analyzing heart tissues obtained from CHD patients or animal models or cells derived from pluripotent stem cells, multi-omics analysis can be used to investigate the molecular mechanisms involved in the regulation of cardiac development at multiple levels. CHD congenital heart disease. This figure was created using Adobe Illustrator

Recent developments in stem cell and bioinformatics techniques, especially the advent of human pluripotent stem cell (hPSC) technology and multi-omics analysis, have ushered our understanding of heart development in a new phase (Fig. 2). Through single-cell RNA sequencing analysis of mesoderm posterior 1 (*Mesp1*^*+*^) cardiac progenitors in mouse embryos at E6.75 and E7.25, Lescroart et al. identified distinct populations of *Mesp1*^*+*^ progenitors committed to different cell lineages and regions of the heart, including endothelial cells, cardiomyocytes (CMs), and anterior and posterior SHF populations, suggesting that *Mesp1*^*+*^ cardiac progenitor cells rapidly segregate from the outer layer of the embryo into distinct cardiovascular lineages.^63^ Single-cell sequencing technology has also enabled precise observation of gene expression and cell fate in FHF and SHF cells. A recent study revealed that primitive streak cells contributing to the ventricles exhibit a distinct molecular signature compared to those forming the OFT and atrium.^64^ Xiong et al. conducted single-cell transcriptomic analysis of *Nkx2-5* and *Isl1* lineages in mouse embryos from E7.75 to E9.25 and revealed that FHF cells differentiate rapidly into CMs, whereas SHF cells undergo gradual transitions to achieve their final cell fate, underscoring finely tuned regulation across multiple stages. Additionally, SHFs are attracted to the FHF-populated heart tube region through chemotactic interlineage communications mediated by macrophage migration inhibitory factor (MIF)—C-X-C motif chemokine receptor 2 (CXCR2).^65^ Another study replicated the development of FHF, anterior SHF, and posterior SHF in hPSCs using different differentiation protocols and identified the genetic characteristics of these cardiac lineages through single-cell sequencing, highlighting the pivotal roles of varying levels of Activin/Nodal and BMP signals in inducing mesoderm differentiation in FHF and SHF.^66^ Table 1 summarizes recent technological advancements in the field of cardiac development.

**Table 1:** Recent single-cell RNA-seq studies on cardiac development

Species	Time	Technologies	Targets	Findings	Ref (PMID)
Mouse	2016	Single-cell RNA-seq	Characterize the transcriptome map of cardiac cells from mouse hearts from E8.5 to E10.5.	*Isl-1* lineage cardiac muscle cells are predominantly classified as related to the OFT or RV, whereas non-*Isl-1* lineage cardiac muscle cells are classified as pertaining to the left ventricle and atrioventricular canal. It has been demonstrated that myocardial cells with *Nkx2-5* defects cannot commit to or differentiate into ventricular cardiomyocytes	27840109
Mouse	2016	Single-cell RNA-seq	Characterize the transcriptome map of cardiac cells from mouse heart from E9.5 to P21	Cardiomyocytes exhibit significant heterogeneity, including subgroups expressing extracellular matrix molecules, cell cycle regulators, and other subsets. Ventricular myocardium shows three transcriptional maturity stages: E9.5-E11.5 expresses genes related to proliferation and morphogenesis; E14.5 to P3 expresses genes associated with myocardial differentiation and metabolic transition; P3 to P21 expresses genes indicative of more mature cardiomyocytes	27840107
Mouse	2018	Single-cell RNA-seq and ATAC-seq	Characterize mouse CPCs marked by Nkx2-5 and Isl1 expression from E7.5 to E9.5	*Isl1*^+^ and *Nkx2-5*^+^ CPCs exhibit remarkable heterogeneity. *Nkx2-5*^+^ CPCs contribute to cardiac lineage differentiation and coexpress markers of both cardiac and smooth muscle cells at E8.5, revealing the enrichment of Hox transcription factors in *Isl1*^*+*^ CPCs branching into cardiac muscle	30451828
Zebrafish	2018	Tomo-seq	Characterize the transcriptome map with high spatial resolution of the developing zebrafish heart from 2 days postfertilization	Identify additional differentially expressed genes in pacemaker cells. *Isl1* induces the Wnt/β-catenin signaling pathway in pacemaker cells to establish parasympathetic control of rhythmic membrane depolarizations	29400650
Zebrafish	2018	Single-cell RNA-seq and ATAC-seq	Characterize the gene expression and accessible chromatin landscape of Smarcd3-F6 enhancer-expressing cells in zebrafish at 10 h postfertilization	162 open chromatin regions overlapping with conserved noncoding elements have been identified. These conserved open chromatin elements drive gene expression in the developing heart	30478328
Mouse	2018	Single-cell RNA-seq	Characterize mouse cardiovascular progenitors marked by *Mesp1* expression from E6.75 to E7.25	Mesp1 promotes epithelial-mesenchymal transition, migration and cardiovascular specification	29371425
Human fetal hearts	2019	Single-cell RNA-seq, spatial transcriptomics and in situ sequencing	Characterize the spatial archetypes and cellular heterogeneity of the developing human heart from 4.5 to 9 PCW	The development of EPDCs begins at approximately 4.5-5 PCW, subsequently promoting the differentiation of several fibroblast-like cell types. Cardiac neural crest cells are present in the mediastinum and OFT during the early stages of heart development. Schwann progenitor cells first appear in later developmental stages in the mediastinum, OFT, and atrioventricular subepicardial mesenchyme regions. Three types of myocardial cells-atrial, ventricular, and Myoz2-enriched myocardial cells-have been identified, with Myoz2-enriched cells present in both atrial and ventricular regions	31835037
Human fetal hearts	2019	Single-cell RNA-seq	Characterize the transcriptome map of human fetal hearts from 5 to 25 PCW	At 5 PCW, 20% of cardiac cells express epicardial cell markers, indicating proepicardial cell presence. From 5 to 6 PCW, there is a sharp increase in the expression of extracellular matrix genes. From 5 to 7 PCW, the Notch signaling pathway promotes differentiation of myocardial cells from the trabecular layer through regulation of the neuregulin/ERBB signaling	30759401
Human fetal hearts/hPSCs	2019	Single-cell RNA-seq	Characterize the transcriptome map of the developing human heart using hPSCs and human fetal hearts from 4.5 to 10 PCW	The human-specific early cono-ventricular region is populated by cardiac progenitor cells marked by LGR5, potentially interacting with RSPO3. LGR5 facilitates the differentiation of ISL1^+^TNNT2^+^ intermediates and promotes myocardial cell induction through human-specific transcriptional interactions such as MESP1-LGR5 and LEF1-ISL1	30713072
Mouse	2019	Single-cell RNA-seq	Characterize cells related to the mouse cardiac conduction system at E16.5	Identification of subpopulations of the sinoatrial node, atrioventricular node, and Purkinje fiber cells, and discovered novel related genes including new sinoatrial node genes *Igfbp5*, *Cpne5*, *Rgs6*, *Ntm*, and *Smoc2*; as well as a new atrioventricular node gene *Cpne5*; and new Purkinje fiber genes *Igfbp5*, *Cpne5*, and *Ntm*	31284824
Mouse	2019	Single-cell RNA-seq	Identify transcriptional characteristics of wild-type and *Hand2*-null mouse heart cells from E7.75 to E9.25	At E7.75, cardiac progenitor cells of the AHF, OFT, and RV precursors in *Hand2*-null embryos show transcriptional dysregulation. By E8.25, the chromatin remodeling gene Smyd1 is downregulated in *Hand2*-null AHF cells. *Hoxa1*, *Hoxb1*, *Upp1*, and *Sema3c* exhibit ectopic expression in OFT and RV cells from E8.5 to E9.25	31341279
Mouse	2019	Single-cell RNA-seq	Characterize the transcriptional features of E13.5 and E14.5 mouse hearts with *Lats1/2* deficiency	*Lats1/2* promotes retinoic acid signaling by inhibiting the negative regulator Dhrs3, enhancing the differentiation of cardiac subepicardial mesenchymal cells into fibroblasts. It also regulates extracellular matrix composition and vascular remodeling	29689192
Mouse	2019	Single-cell RNA-seq	Characterize the *Nkx2.5* and *Isl1* lineages in mouse embryos from E7.75 to E9.25	FHF cells rapidly differentiate into cardiomyocytes, while SHF cells undergo progressive transitions during differentiation to reach their final cell fate, indicating finely regulated processes across multiple stages. Additionally, SHFs are attracted to the FHF-populated heart tube region through chemotactic-guided inter-lineage communication (MIF-CXCR2)	31221018
Mouse	2019	Single cell RNA-seq	Characterize cardiac cells from the mouse heart at E10.5	Using a set of cell cycle genes, each cell’s cell cycle stage was analyzed. AVC myocardial cells exhibit reduced expression of cell cycle genes compared to ventricular myocardial cells. Expression of *Tgfβ1* from the endocardium and *Rspo1* from the epicardium may play roles in establishing a proliferation gradient between compact and trabecular myocardium	31142541
Mouse	2019	Single-cell RNA-seq	Characterize cardiac cells from the mouse heart from E9.5 to E13.5	Constructed MOCA, which includes more than 500 cell types and 56 trajectories	30787437
Zebrafish	2020	Single-cell RNA-seq	Characterize epicardial cells in zebrafish from 3 to 7 days postfertilization	Identified three clusters of epicardial cells (Epi1, Epi2, and Epi3). Epi1 cells expressing *Tcf21*, *Tbx18*, and *Wt1b* primarily promote the complete development of the epicardium. Epi2 cells expressing *Tbx18*, *Acta2*, and *Mylka* may be associated with the smooth muscle layer function during bulbus arteriosus development. Epi3 cells expressing C*xcl12a* may be involved in guiding leukocyte entry into the developing heart	32084358
Mouse	2020	Single-cell RNA-seq	Characterize PEO and epicardial cells in mice from E9.25 to E15.5	PEO cells express *Wt1*, *Sema3d*, *Tcf21*, and *Tbx18*. *Upk3b* serves as a reliable marker for PEO. Differentiation toward the epicardial fate occurs after the initial appearance of mesenchymal cells. Early epicardial cells with high *Wt1* and *Tcf21* levels transition to mesenchymal cells with low *Wt1* but high *Tcf21* levels after undergoing EMT	32359445
Human fetal hearts/hPSCs	2020	Single-cell RNA-seq	Elucidate the transcriptome and functional defects of HLHS endocardium in humans	FN1 is the most significantly downregulated gene in HLHS endocardium. Transcription factor ETS1 and a chromatin remodeler, CHD7, show markedly reduced binding at FN1 promoter and enhancer regions. Lack of FN1 disrupts FN1-integrin α5β1 interactions, impairing hPSC cardiomyocyte growth	32810435
Mouse	2020	Single-cell RNA-seq	Characterize mouse mesodermal cells with the *Gli3R* mutation at E8.25	Deficiencies in the Hedgehog pathway caused by mutant *Gli3R* result in the most pronounced defects in the cranial mesoderm. Significant deficiencies are also observed in the somitic mesoderm and pharyngeal mesoderm, contributing to defects in the anterior cardiac lineage	32561646
hPSCs	2020	Single-cell RNA-seq	Molecular mechanisms of nicotine toxicity on hESC-derived cardiac differentiation	Nicotine exposure during cardiac differentiation leads to the downregulation of neural crest cells, cardiac progenitor cells, and mesodermal cells and the interruption of cardiac-specific transcription factor expression	32276728
Mouse	2021	Single-cell RNA-seq, ATAC-seq and ChIP-seq	From E8.0 to 10.5, elucidating the molecular mechanisms by which CPM cells sustain function and contribute to heart and BrM development	In CPM, identified MLP populations expressing *Tbx1*, *Isl1*, *Mef2c*, *Tcf21*, and *Foxf1*. The *Tbx1* gene promotes key genes for MLP expression in heart and BrM development, while preventing abnormal expression of non-mesodermal genes. *Isl1*, *Foxc2*, and *Six2* are potential direct transcriptional targets of *Tbx1*	34789765
Mouse	2021	single-cell RNA-seq	Characterize the molecular differences among the precursor cells of the ventricle, OFT, and atrium at E7 + 14 hours	The primitive streak cells contributing to the ventricles exhibit a distinct molecular signature compared to those forming the OFT and atrium	33999917
Mouse	2021	Single-cell RNA-seq	Characterize cells from the region corresponding to the embryonic heart from E7.75 to E8.25	A total of 12 cell clusters were identified. Expression, differentiation trajectories, and spatial localization analyses were conducted for 6 clusters associated with heart development	33414188
hPSCs	2022	single-cell RNA-seq	Explore the specific roles of signaling pathways regulating hPSC differentiation in induced hPSC differentiation	In hPSC differentiation, varying concentrations of BMP4 and Activin A play crucial roles in inducing mesodermal specification in the FHF and SHF, and delineating lineage specification and progression of hPSCs through single-cell sequencing techniques	36055193
Mouse	2022	Single-cell RNA-seq, LC-MS/MS, ATAC-seq, spatial transcriptomics and ChIP-seq	Reveal interactive regulatory factors and pathways in the developing mouse heart from E10.5 to postnatal week 8	As cardiac development progresses, there is a physiological decrease in MAPK phosphorylation and an increase in AKT phosphorylation. Potential roles of key transcription and chromatin regulatory factors have been predicted, such as CREB-binding protein (CREBBP) and C/EBP-related transcription factor NFIL-6. Core transcription factors essential for cardiac development and their potential functions have been identified, including TEAD1, TBX5, and RREB1. Metabolic changes characterizing cardiac development have been characterized	36577384
Human fetal hearts/hPSCs	2022	Single-cell RNA-seq and ATAC-seq	Characterize the chromatin and transcriptional landscape of cardiac cells from 6 to 12 PCW	Revealed coordinated landscapes of dynamic cis-regulatory elements and genes defining major cell types, lineages, and differentiation trajectories at early and mid-gestational time points. Identified several mouse transcription factors crucial in determining terminal differentiation cell fates, such as SOX17 and SOX18. Predicted the impact of de novo noncoding mutations on cell type-specific chromatin accessibility profiles. Identified a ranking of cell types enriched for prioritized CHD mutations within their cis-regulatory elements	36563664
Mouse	2022	Single-cell RNA-seq	The impact of maternal hyperglycemia on mouse embryo hearts from E9.5 to E11.5	Maternal hyperglycemia primarily affects *Isl1*^+^ SHF progenitor cells and *Tnnt2*^+^ cardiomyocytes	35970860
Mouse	2023	Single-cell RNA-seq	Characterize cardiac conduction system cells in the embryonic heart from E8.5 to P3	Provides a robust resource for studying the development of the heart’s cardiac conduction system and other cardiac components	37666871
Mouse	2023	Single-cell RNA-seq	Analyze patterns and cell-specific distributions of coding and noncoding RNAs from E9.5 to P0	Identified eight types of cardiac cells and several new coding, lncRNA, and pcRNA markers	38110334
hPSCs	2023	Single-cell RNA-seq	Identifying the FHF origin and left ventricular identity of hPSC-derived cardiomyocytes	Using a small molecule WNT-based 2D differentiation protocol, TBX5-positive cardiomyocytes constitute over 95% of all generated cardiomyocytes in two hiPSC cell lines, indicating a gradual upregulation of FHF markers	37284748
Drosophila	2023	Single-cell RNA-seq	Characterize *Drosophila* cardiac cells marked by *Hand* expression during developmental stages 13-16	Six distinct cardiac cell types are identified in the embryonic fly heart: cardioblasts, including both Svp^+^ and Tin^+^ subtypes; and five types of pericardial cells distinguished by four key transcription factors (*Eve*, *Odd*, *Ct*, and *Tin*), which include the newly described ‘end of the line’ pericardial cell	37526610
Mouse	2023	Single-cell RNA-seq	Study of transcriptomic changes induced by conditional deletion of *Hand2* in endocardium using *Nfatc1*Cre at E11.5	*Hand2* in the endocardium acts on target genes such as *Klf2* and *Igf2*, influencing multiple endocardial transcriptional networks beyond the Notch pathway, particularly in shear‒stress response, revealing multiple significant roles of *Hand2* in endocardial morphogenesis	36620995
Mouse	2023	Single-cell RNA-seq	Reveal the genetic characteristics and dynamic changes of CNCCs under normal conditions and when *Tbx1* is inactive from E8.5 to E10.5	Determined cell type markers and dynamic cell fate of CNCCs; altered BMP and FGF-MAPK signaling pathways, along with other signaling pathways, when *Tbx1* is inactive, may lead to cardiovascular abnormalities; identified genes and ligand-receptor pairs associated with intercellular communication between mesodermal cells and CNCCs	36941249
Human fetal hearts	2024	Single-cell RNA-seq and MERFISH imaging	Characterize cardiac cells from human fetal hearts between 9 and 16 PCW	Constructed a comprehensive cell atlas of developing human hearts with spatial and molecular single-cell resolution. Identified newly discovered cardiac cell populations and multicellular interactions	38480880
Human fetal hearts/Mouse	2024	Single-cell RNA-seq	Aligning the transcriptional developmental stages of mouse and human hearts	Mouse atrial cardiomyocytes at E9.5 to E13.5 correspond to approximately 5-6 weeks of human embryonic age, while ventricular cardiomyocytes correspond to 13-15 weeks of human embryonic age. Endothelial cells in the mouse heart correspond to embryos of approximately 6-7 weeks in humans	38542214
Human fetal hearts	2024	Single-cell RNA-seq	Profiling the gene expression landscapes of human fetal hearts from the four-time points: 8, 10, 11, and 17 PCW	Providing a comprehensive map of the cellular diversity and transcriptional of human fetal hearts. Dynamic cell-cell communication was identified in the process of fetal heart development	38876166
hPSCs	2024	Single-cell RNA-seq	The molecular mechanisms of PFOS toxicity on hESC-derived cardiac differentiation	After PFOS exposure, there is an increase in primitive endoderm cells and a decrease in the proportion of cardiac progenitor cells and cardiomyocytes, resulting in aberrant differentiation of hESCs into cardiac cells	38183750
Mouse	2024	Single-cell RNA-seq	Characterize *Tbx18*-positive cardiac cells in mice from E7.5 to P21	*Tbx18*^*+*^ cardiac cells can be classified into at least two cell types with distinct gene expression profiles: fibroblast-like cells and cardiomyocytes. During the late developmental stages of *Tbx18*^+^ cardiac cells, there is an increase in the expression of extracellular matrix and EMT genes	38265516

*Acta2* actin alpha 2, *AHF* anterior heart field, *ATAC* assay for transposase-accessible chromatin, *AVC* atrioventricular canal, *BMP* bone morphogenetic protein, *BrM* branchiomeric skeletal muscles, *CHD* congenital heart disease, *CHD7* chromodomain-helicase-DNA-binding protein 7, *ChIP* chromatin immunoprecipitation, *CNCC* cardiac neural crest cell, *CPC* cardiac progenitor cells, *CPM* cardiopharyngeal mesoderm, *Cpne5* copine 5, *CREBBP* CREB-binding protein, *Cxcl12a* C-X-C motif chemokine ligand 12a, *CXCR2* C-X-C motif chemokine receptor 2, *Dhrs* dehydrogenase, *EMT* epithelial-mesenchymal transition, *EPDC* epicardium-derived cell, *ETS* ETS proto-oncogene, *FGF* fibroblast growth factor, *FHF* first heart field, *FN* fibronectin, *Fox* Forkhead box, *Hand2* heart and neural crest derivatives expressed 2, *Gli3R* glioma-associated oncogene homolog 3, *HLHS* hypoplastic left heart syndrome, *Hoxa* homeobox, *hPSC* human pluripotent stem cell, *Igf2* insulin-like growth factor, *Igfbp5* insulin-like growth factor binding protein 5, *Isl1* Islet-1, *Klf* krüppel-like factor, *Lats* large tumor suppressor kinase, *LC-MS/MS* liquid chromatography-tandem mass spectrometry, *LEF1* lymphoid enhancer-binding factor 1, *LGR5* leucine-rich repeat-containing G-protein coupled receptor 5, *lncRNA* long non-coding RNA, *MAPK* mitogen-activated protein kinase, *Mef* myocyte-specific enhancer factor, *MERFISH* multiplexed error-robust fluorescence in situ hybridization, *MIF* macrophage migration inhibitory factor, *MLP* multilineage primed progenitor, *MOCA* mouse organogenesis cell atlas, *Mylka* myosin light chain kinase a, *Myoz2* Myozenin 2, *NFIL-6* nuclear factor interleukin-6, *Nkx2-5* NK2 homeobox 5, *Ntm* neurotrimin, *OFT* outflow tract, *PC* pericardial cell, *pcRNA* protein-coding RNA, *PCW* post-conception week, *PEO* proepicardial organ, *PFOS* perfluorooctane sulfonate, *Rgs6* regulator of G-protein signaling 6, *RREB1* Ras-responsive element binding protein 1, *RSPO* R-spondin, *RV* right ventricle, *Sema* semaphorin, *SHF* second heart field, *Six* Sine oculis homeobox homolog, *Smarcd3* SWI/SNF-related matrix-associated actin-dependent regulator of chromatin subfamily D member 3, *Smoc2* SPARC-related modular calcium-binding protein 2, *SOX* sex-determining region Y-box, *Svp* slit ventricle, *TEAD1* TEA domain transcription factor 1, *Tbx* t-box transcription factor, *Tcf21* transcription factor 21, *Tin* tinman, *Upk* uroplakin, *Upp1* uridine phosphorylase 1,*WT1* wilms tumor 1

## The molecular mechanisms of cardiac development

**Fig. 3:**
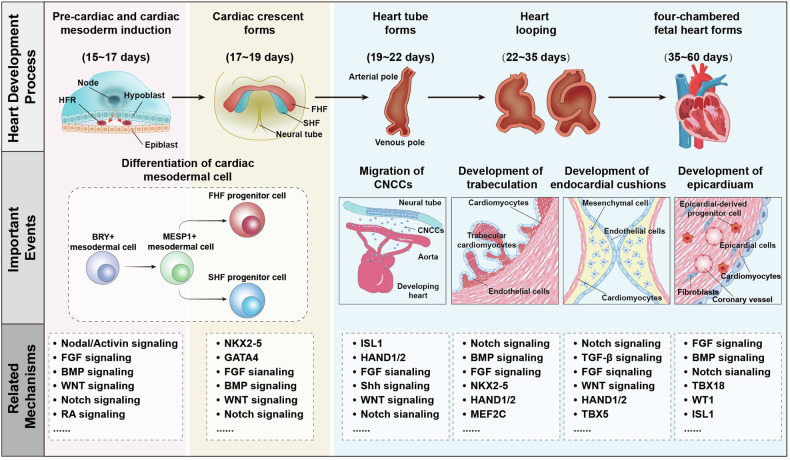
Human cardiac development and its regulatory mechanisms. Cardiac development involves five distinct stages. The first stage, mesoderm cardiac induction (15-17 days), begins with cardiac mesoderm progenitors migrating from the primitive streak to the heart-forming region, an anterior lateral area relative to the streak. During migration, these mesodermal cells acquire the ability to differentiate into the cardiac lineage and express markers such as Brachyury and MESP1. The key signaling pathways involved include the Nodal/Activin, BMP, and WNT signaling pathways. In the second stage (17–19 days), the cardiac crescent forms, and MESP1-derived cardiac mesodermal cells differentiate into FHF and SHF progenitors. Molecular signals, including WNT signaling, BMP signaling, and various TFs, are critical. FHF progenitor cells, positioned more anteriorly and laterally in the crescent, readily respond to molecular cues and begin differentiation. SHF progenitors remain proliferative and undifferentiated until they reach later stages when they contribute to the heart tube. The third to fifth stages include heart tube formation (19–22 days), cardiac looping (22–35 days), and the formation of the four-chambered fetal heart (35–60 days). During these stages, the cardiac crescent fuses at the midline and folds into a Y-shaped FHF-derived linear heart tube. SHF cells proliferate rapidly and contribute to the arterial and venous poles of the heart tube. Subsequently, the heart tube undergoes rapid growth and rightward looping, forming distinct chambers by approximately day 32 in humans that are fully septated and connected to the pulmonary trunk and aorta by week 7 of fetal development. Events such as the migration of CNCCs, myocardial trabeculation, and the development of endocardial cushions and the epicardium are crucial during these stages. Signaling pathways, including FGF signaling, Notch signaling, and Shh signaling, are involved in the migration of CNCCs, promoting the development of the aortic arch and outflow tract. FGF signaling, Notch signaling, BMP signaling, and others contribute to myocardial trabeculation. Furthermore, FGF signaling, Notch signaling, TBX5, HAND1/2, and related signals aid in the development of endocardial cushions, facilitating subsequent septum and valve development. Finally, FGF signaling, TBX18, WT1, Notch signaling, and others contribute to epicardial development, promoting coronary artery formation and subsequent proliferation of myocardial cells. BRY brachyury, BMP bone morphogenetic protein, CNCCs cardiac neural crest cells, FGF fibroblast growth factor, FHF first heart field, GATA GATA binding protein, HAND heart and neural crest derivatives expressed, HFR heart-forming region, ISL1 islet 1, MEF myocyte enhancer factor, MESP1 mesoderm posterior 1, NKX2-5 NK2 Homeobox 5, RA retinoic acid, Shh sonic hedgehog, SHF second heart field, TBX T-box transcription factor, TGF transforming growth factor, TFs transcription factors, WT1 Wilms tumor 1. This figure was created using Adobe Illustrator

To better understand the molecular mechanisms underlying cardiac development, it is essential to investigate the process of heart development. Various animals, such as chicks, mice, and human embryos, have been utilized in studies of cardiac development. Despite species differences, there are similarities in the processes and regulatory signals involved. Here, we primarily focused on human heart development to illustrate the developmental process (Fig. 3). In general, heart formation encompasses several stages, including pre-cardiac and cardiac mesoderm induction, formation of the cardiac crescent, heart tube formation, cardiac looping, and the formation of the four-chambered fetal heart. After birth, further maturation of cardiomyocytes involves cell cytoskeletal structure, metabolism, and a decrease in proliferation with occasional incomplete cell cycles. During the first week after birth, most cardiomyocytes complete their final cell division. Subsequently, the increase in heart size is largely achieved through the hypertrophy of cardiomyocytes. Recent reviews have extensively discussed cardiac development during this stage.^67–69^ This review focuses primarily on prenatal development. Cardiac development initiates when cardiac mesoderm progenitors migrate away from the primitive streak and coalesce in an anterior lateral region relative to the streak, known as the heart-forming region (HFR). These mesodermal cells acquire the ability to differentiate into cardiac lineages during migration, characterized by the expression of the *MESP1* gene, which subsequently gives rise to both FHF and SHF progenitors.^70,71^ This process is regulated by various signaling pathways and molecules, including Activin/Nodal, BMP, FGF, and WNT/β-catenin signaling.^1,72–74^ Concurrently, as cardiac mesoderm cells migrate and differentiate, some undergo epithelial-to-mesenchymal transition (EMT) to form endocardial cells between the primary cardiac mesoderm and endoderm. These endocardial cells organize into a small network of channels that merge into larger channels as development progresses.^75^

At approximately week 2 of human gestation, these cardiac mesoderm cells in the HFR form the horseshoe-shaped cardiac crescent, with the commitment to a cardiac fate remaining flexible.^70^ During this phase, *MESP1*^*+*^ cells further differentiate into FHF and SHF progenitors. Due to their positioning in the crescent (more anterior and lateral relative to SHF progenitors), FHF progenitors are more susceptible to regulation by BMP and FGF families, as well as inhibitors of the WNT pathway, which initiates their differentiation.^76,77^ In contrast, at a later stage, SHF progenitors remain in a proliferative undifferentiated state until they ingress into the heart tube.^78^

At approximately week 3 of human gestation, the cardiac crescent undergoes rapid growth and rightward looping, forming a linear heart tube with two posterior inflow tracts (venous poles) and one anterior outflow tract (arterial pole or outflow tract). The heart tube consists of the outer layers of two to three layers of myocardial cells and the inner layers of endocardial cells separated by the extracellular matrix (ECM) called cardiac jelly. At this stage, the heart tube begins to display slow contractions originating from the venous poles, and a sinusoidal morphology can be observed in electrocardiograms.^79^ Subsequently, SHF precursor cells from the dorsal and caudal regions of the pericardial cavity continue to migrate to the venous and arterial poles of the heart tube. SHF cells proliferate at a high rate mediated by classical WNT/β-catenin signaling and contribute to the arterial and venous poles of the heart tube during differentiation.^80,81^ Simultaneously, cardiac neural crest cells (CNCCs) delaminate from the neural tube and migrate toward the heart via preestablished pathways mediated by the BMP, FGF, WNT/β-catenin, and RA signaling pathways.^82^

At approximately the end of week 3 of human gestation, the heart tube undergoes further growth and rightward looping, with well-defined chambers visible around day 32 of human development. By week 7, the fetal heart was fully septated and connected to the pulmonary trunk and aorta.^1,83,84^ During this phase, SHFs continue to migrate to the venous and arterial poles of the heart tube. Myocardial cells in the outer curvature of the heart tube and atrial myocardial cells undergo transcriptional programs associated with chamber formation and re-entry of the cell cycle and proliferation, leading to ventricular “ballooning”.^85–87^ Concurrently, the cardiac jelly between the endocardial and myocardial layers disappears, and myocardial trabeculae become evident on the endocardial side of the chamber. Ventricular chambers start forming trabeculae under the control of Notch, Neuregulin (NRG), Ephrin (EFN), and BMP10 signalings.^88,89^ Subsequently, the outer layer of the myocardium begins to compact, reducing proliferation in myocardial trabeculae. During this period, development progresses in the cardiac septum, valves, epicardium, and conduction system. Atrial septation begins during the fourth developmental period and is mediated by the proliferation of atrial cells. Simultaneously, as the right ventricle and left ventricle form, the ventricular septum also begins to form.^86^ The septation process starts with the expansion of the cardiac jelly between the endothelial cell layer and myocardial cell layer of the atrioventricular canal (AVC) and OFT, which primarily involves posterior and anterior atrioventricular cushions in the AVC and parietal and septal outflow cushions in the OFT. Initially filled by endocardium-derived mesenchymal cells, Notch, BMP, and TGF-β signalings regulate the endocardial-to-mesenchymal transition (EndMT) in cushions.^90–92^ In addition to contributions from cells in the cushions, semilunar valves in the aorta and pulmonary trunk receive extracardiac contributions from CNCCs.^90,93^ These cells migrate to the heart via specific pathways mediated by EFN, FGF, semaphorins, and connexin 43,^94^ contributing to the development of the aortic arch arteries and OFT. Ablation of CNCCs results in disrupted Ca^2+^ handling and depressed ejection fraction,^95^ an interrupted aortic arch,^96^ a shortened OFT,^97^ and abnormal OFT septation.^98^

During this phase, the sinus venosus and sinoatrial node precursor are formed from T-box transcription factor (TBX)-5^+^, TBX18^+^, and hyperpolarization-activated cyclic nucleotide-gated potassium channel 4 (HCN4^+^) SHF progenitor cells. The dominant pacemaking activity gradually moves to the final sinoatrial node area.^99,100^ Developing ventricular and atrial chamber muscles acquire rapid cell-to-cell conduction properties due to the expression of connexin 40, connexin 43, Nav1.5, and other ion-handling proteins.^101^ Parts of the trabecular myocardium of the ventricles remodel into the Purkinje fiber network. The electrocardiogram of the heart also begins to transition from a sinusoidal waveform to a waveform resembling that of a mature heart, characterized by P waves, PR intervals, and QRS complexes.

The proepicardium (PE) arises around day 21 of human pregnancy from the coelomic mesenchyme of the septum transversum near the venous pole of the linear heart tube. Its development is primarily mediated by FGF and BMP signaling.^102^ These cells specifically express TBX18 and Wilms tumor 1 (WT1)^103^ and are released from the PE precursor by PE cell vesicles, flattening and spreading upon contact with the exposed myocardium to form the epicardium. The formed epicardium contributes to the formation of cardiac fibroblasts, vascular smooth muscle cells, and valves through epicardial EMT and the formation of epicardium-derived cells (EPDCs), mediated by TGF-β, Notch, and RA signals.^104–107^ The epicardium also secretes GFs such as FGF9, insulin-like growth factor (IGF)-2, and RA, which regulate the proliferation of ventricular myocardium closely adjacent to the epicardium.^67,104^ Next, we will provide a detailed summary of each important molecular mechanism regulating cardiac development and its effects at different stages of development.

### Growth factors

Numerous studies have shown that GFs can mediate embryonic heart growth, determination, and differentiation through intercellular signaling at various stages of cardiac development. Among the most critical are the FGF family and members of the TGF-β family, including TGF-β1, TGF-β2, TGF-β3, BMP, Nodal, and Activin (Table 2). The FGF family comprises 22 multifunctional proteins identified in humans and mice that significantly influence early mesoderm induction, FHF and SHF formation, cardiac neural crest, and epicardium development.^108^ Previous studies have shown that FGF1 and FGF2 can induce mesoderm from naive prospective ectodermal cells in *Xenopus*.^109,110^ Further research revealed that paraxial mesoderm induction requires FGF signaling, while axial mesoderm induction relies on FGF signaling primarily for maintenance.^111^ FGF2 is also expressed in the PE, stimulating its differentiation into the epicardial lineage. In the proepicardial base, coexpression of BMP2 and FGF2 inhibits myocardial and epicardial differentiation.^102^ FGF4 and FGF8, along with their receptor FGFR4, are crucial for proper patterning of the paraxial mesoderm in the gastrula and left-right organizer, and their dysfunction leads to the loss of key genes involved in symmetry breakage, resulting in CHD associated with heterotaxy.^112^ FGF4 and FGF8 signal primarily through Hedgehog (Hh) signaling in the mesoderm.^113^ During the heart tube formation and looping stages, FGF8 and FGF10 are detectable in SHF.^53,114^ Abnormal *Fgf8* expression results in significant loss of the *Nkx2-5-*Cre lineage and severe outflow and RV truncations by E9.5 in mouse embryos, mediated by decreased cell proliferation and aberrant cell death in both the pharyngeal endoderm and splanchnic mesoderm, while the remaining heart chambers (left ventricle and atria) appear grossly normal.^115^ Another study revealed that mesodermal *Fgf8* is required for the correct alignment of the OFT and ventricles, while *Fgf8* from the pharyngeal endoderm regulates outflow tract septation.^116^ High levels of FGF8 expression in the pharyngeal endoderm and ectoderm exhibit a chemotactic effect on cardiac crest cells, mediated by FGFR1 and FGFR3 along with mitogen-activated protein kinase (MAPK)/extracellular signal-regulated kinase (ERK) intracellular signaling.^117^ FGF8 and FGF10 are involved in SHF progenitor proliferation, influencing the OFT and RV.^60^ Mutations in the FGF10 receptor *Fgfr2-IIIb* also lead to an underdeveloped OFT and RV, along with VSD associated with an overriding aorta or double outlet right ventricle (DORV), and include thin myocardial walls, trabecular abnormalities, and muscular VSD. *Fgf10* and *Fgfr2-IIIb* mutant embryos lack pulmonary arteries and veins.^118^ Analysis of *Fgf10*^-/-^ hearts and primary cardiomyocyte cultures revealed that *Fgf10* regulates myocardial cell proliferation in fetal heart regions via the Forkhead Box O3 (FOXO3)/p27(kip1) pathway.^119^ FGF15 is present in the pharyngeal endoderm. Deficiency of *Fgf15* results in heart defects consistent with malalignment of the aorta and pulmonary trunk, which correlates with early morphological abnormalities in the outflow tract due to abnormal behavior of the cardiac neural crest.^120^

**Table 2:** Growth factors and transcription factors are involved in cardiac development

			Functions in cardiac development	Consequence of Loss of function	References
Growth factors	FGF family	FGF1	Involved in mesoderm induction; Involved in coronary development		^110,126^
		FGF2	Involved in mesoderm induction; Regulation of the differentiation of epicardium; Involved in coronary development		^102,109,110,126^
		FGF4	In the early phase, involved in left-right organizer patterning;	Heterotaxy	^112,113^
		FGF7	Involved in coronary development		^126^
		FGF8	Involved in left-right organizer; Involved in the proliferation of SHF progenitor cells; Involved in the development of outflow and right ventricle; Regulation of outflow tract septation; Chemotactic effect on cardiac neural crest cells	Heterotaxy; Abnormal OFT and RV development	^60,112,113,115–117^
		FGF9	Regulation of proliferation of cardiomyocytes, cardiac progenitor cells and cardiac fibroblasts; Involved in atrial and ventricular development; Involved in coronary development	Tissue loss in the apex of the heart and the area around the interventricular sulcus; Atrial enlargement; Biventricular dilation; Abnormal coronary development	^121,124,125^
		FGF10	Involved in the proliferation of SHF progenitor cells; Involved in the development of outflow and right ventricle; Involved in the development of ventricular septal, conotruncus, myocardial trabeculation and pulmonary vessels; Regulation of proliferation of cardiomyocytes	Abnormal OFT and RV development; VSD; Overriding aorta; DORV; Abnormal myocardial trabeculation; Lack of pulmonary arteries and veins	^53,60,118,119^
		FGF15	Involved in the development of outflow tract	Malalignment of the aorta and pulmonary trunk	^120^
		FGF16	Regulation of proliferation of cardiomyocytes, cardiac progenitor cells and cardiac fibroblasts; Involved in ventricular and trabecular development	Dilation of the ventricle with compromised trabeculae	^122–124^
		FG20	Regulation of proliferation of cardiomyocytes		^121^
	TGFβ family	Activin/Nodal	Involved in left-right organizer; Specification of mesendodermal cell fates; Involved in primitive streak and mesoderm formation	Heterotaxy; Lack of mesoderm and heart tissue	^128–136^
		BMP2	Involved in mesoderm and FHF formation; Involved in the development of cardiac cushion in AVC; Involved in epicardial maturation	Lack of cardiac crescent and primitive ventricle; Abnormal cushion development	^102,142,162,167^ ^231^
		BMP4	Involved in mesoderm and FHF formation; Regulation of proliferation in cushion; Involved in the development of AVC, arteries and OFT septation; Involved in the differentiation of pacemaker cells; Involved in epicardial maturation	Lack of cardiac crescent and primitive ventricle; Atrioventricular canal defect; Abnormal morphogenesis of branchial-arch arteries; OFT septation defect	^134,137–139,144^ ^145,163^
		BMP10	Regulation of proliferation of cardiomyocytes; Involved in heart wall thickness	Severely thinned ventricular wall	^143^
		TGF-β1	Involved in the development of cardiac cushion, including regulation of proliferation of mesenchymal cells and EMT; Involved in the development of valves and vessels; Involved in epicardial and coronary development	Abnormal AVC development; Abnormal coronary development; Thinned myocardium	^150–152,155–157^ ^165,166^
		TGF-β2	Involved in the development of cardiac cushion, including regulation of proliferation of mesenchymal cells and EMT; Involved in the development of septation and valves; Involved in epicardial and coronary development	Enlarged valves; ASD; VSD; Abnormal coronary development; Thinned myocardium	^153–157,165–169^
		TGF-β3	Involved in the development of cardiac cushion, including regulation of proliferation of mesenchymal cells and EMT; Involved in epicardial and coronary development	Abnormal AVC development; Abnormal coronary development; Thinned myocardium	^155–157,165,166^ ^168,169^
Transcriptional factors		NKX2-5	Involved in early cardiac morphogenesis, differentiation of cardiomyocytes and cardiac ventricle formation; Involved in the differentiation of cardiac endothelial cells and smooth muscle cells; Involved in the development of septation, valves, conotruncus and pulmonary myocardium; Involved in the development of the cardiac Purkinje fiber network	Various cardiac malformations (ASD, VSD, AVSD, ToF, PDA, TGA, et al.); Conduction defects	^7,62,65,175–179^ ^181–183^
		GATA4	Involved in early cardiac morphogenesis and differentiation of cardiomyocytes and cardiac ventricle formation; Involved in the development of septation, valves, conotruncus and heart wall thickness;	Various cardiac malformations (ASD, AVSD, VSD, PDA, TOF, DORV, et al.)	^184–188,191,193^
		ISL1	Involved in SHF formation; Involved in septation, atrial, OFT and right ventricle development Involved in coronary development;	Lack of OFT, RV, and atria; VSD; DORV	^59,65,179,194,195^ ^197^
		PITX2	Regulation of left-right specification of the atria; Involved in OFT development; Involved in valves and pulmonary myocardial sleeve development	Heterotaxy; Swelling atrioventricular canals; Atrium juxtaposition; Lack of tricuspid and mitral valves; Sinoatrial node and pulmonary myocardial sleeve defects	^180,198–203^
		NFYa	Regulation of proliferation and metabolism of cardiomyocytes		^204^
		HAND1	Involved in left ventricle and cardiac cushion formation; Involved in myocardial trabeculation and compaction	Left ventricle defects; Endocardial cushion defects	^209–211^
		HAND2	Involved in the right ventricle and OFT formation; Involved in heart wall thickness, valves and septation development; Involved in myocardial trabeculation and compaction; Involved in epicardial development	Hypoplasia of the right ventricle; Thin myocardium; Dilated aortic sac; VSD; OFT defects; Tricuspid atresia; Double inlet left ventricle; Reduced ventricular trabeculation; Abnormal coronary development	^50,206–208,211–215^
		MEF2C	Involved in SHF formation; Involved in heart tube looping; Involved in cardiac ventricle formation; Involved in OFT, septation and right ventricle development	VSD; Single ventricular chamber;	^218,222,223^
		TBX1	Regulation of differentiation of multilineage-primed cells into SHF cells; Regulation of proliferation of SHF cells; Regulation of cardiac progenitor cell differentiation; Involved in aortic arch and OFT development	Aortic arch patterning defects; OFT defects; VSD; ToF	^225–229^
		TBX2	Involved in the development of cardiac cushions and chamber differentiation	Lack of AVC	^230,231^
		TBX3	Involved in the development of cardiac cushions and chamber differentiation	Lack of AVC	^231^
		TBX5	Involved in SHF development and heart tube looping; Involved in the development of septation, valves and heart wall thickness; Involved in cardiac conduction system development	ASD; VSD; AVSD; Thin myocardium wall	^217–222^
		TBX18	Involved in sinus venosus and epicardial development	Abnormal coronary development	^232–236^
		TBX20	Involved in AVC, valve, OFT and RV development	Atrioventricular canal constriction; Outflow tract and right ventricular hypoplasia	^237–239^
		WT1	Involved in epicardial development	Abnormal coronary development	^232–234^
		TCF21	Involved in epicardial development	Abnormal coronary development	^232–234^

*ASD* atrial septal defect, *AVC* atrioventricular canal, *AVSD* atrioventricular septal defect, *BMP* bone morphogenetic protein, *DORV* double outlet right ventricle, *EMT* epithelial-mesenchymal transition, *FGF* fibroblast growth factor, *FHF* first heart field, *GATA4* GATA binding protein 4, *HAND1* heart and neural crest derivatives expressed 1, *HAND2* heart and neural crest derivatives expressed 2, *ISL1* islet 1, *MEF2C* myocyte enhancer factor 2C, *NFYa* nuclear transcription factor Y subunit alpha, *NKX2-5 NK2* homeobox 5, *OFT* outflow tract, *PDA* patent ductus arteriosus, *PITX2* paired like homeodomain 2, *RV* right ventricle, *SHF* second heart field, *TBX* T-box transcription factor, *TCF21* transcription factor 21, *TGA* transposition of the great arteries, *TGFβ* transforming growth factor beta, *ToF* Tetralogy of Fallot, *VSD* ventricular septal defect, *WT1* Wilms tumor 1

The FGF9 subfamily includes FGF9, -16, and -20, which control myocardial differentiation and proliferation. FGF9, FGF16, and FGF20 are expressed in the endocardium and epicardium and signal specifically to the myocardium via FGFR1c and FGFR2c. *Fgf9* knockout hearts exhibit disproportionate tissue loss in the heart apex and interventricular groove, reduced myocardial proliferation around the interventricular groove, atrial enlargement, and biventricular dilation.^121^ *Fgf16* deficiency in embryonic hearts results in different outcomes in various mouse models. In C57 embryonic mice, *Fgf16* deficiency results in slight decreases in heart weight and myocardial cell number by 6 months of age.^122^ In Black Swiss mice, *Fgf16* deficiency leads to in-utero death, with common (or primitive) ventricular dilation and compromised trabeculae.^123^ These differences may stem from the distinct genetic backgrounds of the different mouse models. In the C57 embryonic mice, cardiac development does not completely depend on FGF16 for growth and differentiation. Specific genetic factors in C57 embryonic mice may rescue the embryonic lethality caused by *Fgf16* deficiency. For example, relatively high levels of FGF9 in the C57BL/6 strain and the heightened sensitivity of Black Swiss mice to reduced FGF8 levels may explain the rescue of the embryonic lethality phenotype.^123^ Recent research has shown that FGF16 and FGF9 bind to different FGF receptors on cardiac progenitor and fibroblasts, promoting their proliferation. FGF16 also enhances proliferation in naive cardiac progenitor cells isolated from mouse hearts and human cardiomyocytes derived from induced pluripotent cells.^124^ Notably, functional redundancy may exist among the same FGF subfamily members; FGF16 and FGF20 likely act redundantly with FGF9 during cardiac development, signaling through FGFR1c and FGFR2c in the myocardium, similar to FGF9.^121^ Additionally, FGFs, including FGF1, -2, -7, and -9, are involved in angiogenesis and coronary development.^125,126^

The TGFβ family can be broadly divided into the TGFβ cluster and the BMP cluster. TGFβ and Activin ligands bind to specific receptor combinations to inhibit decapentaplegic (SMAD) 2/3 phosphorylation, while BMPs bind to receptors, leading to SMAD1/5/8 phosphorylation. Phosphorylated SMADs form complexes with SMAD4 and translocate to the nucleus to induce gene transcription.^127^ TGF-β family members also play significant roles in mesoderm induction and patterning. One of the earliest roles of TGF-β signaling in vertebrate development is the specification of mesendodermal cell fates by Nodal signaling. Loss of Nodal signaling results in the failure to form mesodermal and endodermal tissues.^128,129^ Nodal signals through Activin type II receptors (ActRII or ActRIIB) and primarily act on type I receptors ActRIB/ActR-like kinase (ALK)-4 or ALK7. Activin acts through ActRII or ActRIIB in conjunction with Alk4 or ActRIA/ALK2.^72^ Nodal family members are involved in patterning the mesoderm.^130^ As coreceptors of Nodal, Cripto is crucial for gastrulation and mesoderm formation. Cripto deficiency leads to the loss of somite and heart tissue in embryos, as well as the absence of cardiac-specific markers such as myosin heavy chain (*Myh*)*-6*, *Myh7*, myosin light chain (*Myl*)-*2*, *Myl7*, and natriuretic peptide A (*Nppa*).^131,132^ The absence of Activin-related receptors also disrupts primitive streak and mesoderm formation.^133–136^

At least 20 structurally and functionally related BMPs, including BMP-2, BMP-4, and BMP-10, which play roles in cardiac development, have been identified. Three receptor types, including BMP receptors, Activin receptors (ActRII, ActR-IIB), and an even larger number of type I receptors termed ALKs, mediate BMP binding, such as BMP2 and BMP4 binding to ALK3 and ALK6.^72^ Deficiency of *Bmp4* and the BMP receptor causes embryonic lethality at E9.5, highlighting their crucial role in gastrulation and primitive mesoderm formation.^134,137–139^ Conditional deletion of *BmpR1a* in the *Mesp1*-Cre lineage results in the absence of the cardiac crescent and later primitive ventricle, indicating the essential role of BMP receptor signaling in FHF formation.^140^ Similarly, another study using *Mox2*-Cre to delete BmpR1a in the epiblast conditionally led to the lack of a distinct cardiac crescent structure or subsequent cardiac tissues.^141^

TGFβ and BMP signaling also play crucial roles in the later stages of cardiac development. *Bmp2* deficiency leads to abnormal heart development in the exocoelomic cavity.^142^ *Bmp10*-deficient mice exhibit significantly reduced myocardial cell proliferation and severely thinned ventricular walls, accompanied by downregulation of the cardiac TFs *Nkx2-5* and *Mef2c*.^143^ Conditional deletion of *Bmp4* in the *Tnnt2* or *Nkx2-5*-Cre linages results in AVC defects, abnormal morphogenesis of branchial-arch arteries, and defective OFT septation.^144,145^ Additionally, the contribution of TGF-β/BMP signaling to cushion development underscores its role in valve and septa development. During development, endocardial cells in the AVC and OFT become hypertrophic and undergo EndMT, transforming into mesenchymal cells that migrate into the cardiac jelly, eventually forming cardiac cushions.^146^ As EndMT progresses and mesenchymal cells proliferate, cardiac cushions continue to grow and develop into valves and septa. In mice, *Tgfb1* and *Tgfb2* are expressed in the endocardium and endothelium during development, particularly in the AVC and OFT, with *Tgfb3* expression beginning after the onset of EndMT.^147–149^ Although single *Tgfb1* or *Tgfb3* deletions do not cause cardiac malformations, embryos and mothers lacking *Tgfb1* show severe cardiac abnormalities, including valve disarray and vascular defects, indicating that maternally derived TGF-β1 is sufficient to rescue the cardiac phenotype in these knockout mice.^150–152^ *Tgfb2* gene inactivation leads to heart defects, including atrial septal defects (ASDs) and VSDs, as well as enlarged cushions and valves.^153,154^ TGF-β1, -2, and -3 bind to TGFβRII with high affinity, activating ALK5 and downstream signaling. *Alk5* deficiency leads to reduced mesenchymal cell proliferation and EndMT.^155,156^ Although mice with myocardial-specific *Alk5* deletion do not exhibit cardiac defects, mice with endocardial-specific *Alk5* deletion exhibit severely underdeveloped AV cushions, leading to septal defects.^157^ Further studies indicated that *Tgfb2* activation in the endocardium requires interaction with TGFbRIII, without which the endothelial cell-cell separation step is inhibited.^158,159^ BMP ligands BMP2, -4, -5, -6, and -7 are expressed in the myocardium, covering the AVC and OFT.^160,161^ Myocardial-specific *Bmp2* knockout results in reduced cardiac jelly and acellular cushion formation in the AVC.^162^ *Bmp4* deficiency reduces cushion growth due to impaired proliferation, leading to OFT septation defects.^145^ However, another study suggested that *Bmp4* is not required for EndMT indicating that *Bmp4* mainly participates in the expansion and remodeling of the OFT endocardial cushion, rather than its initial EndMT, which results in severe OFT and VSDs.^163^ Notably, *Bmp4* deficiency results in the upregulation of *Bmp7* in the OFT myocardium, and compound knockout mice lacking both *Bmp4* and *Bmp7* show severely underdeveloped OFT cushions, indicating potential redundancy among *Bmp4* and *Bmp7* ligands.^145^ Additionally, in human pluripotent stem cells, BMP4 plays a crucial role in pacemaker cell differentiation, promoting the appearance of sinoatrial node CMs in conjunction with RA and Wnt signaling.^164^

TGF-β family members are also critical for epicardial development. *Tgfb2* is expressed in the PE as early as E9.5, and *Tgfb3* is expressed throughout the epicardium from E11.5 onward.^147,165^ Conditional deletion of *Alk5* in epicardial cells using *Gata5*-Cre disrupts interactions between the epicardium and myocardium, leading to myocardial thinning, defects in the smooth muscle cell layer surrounding coronary arteries, and abnormal capillary formation in the myocardium.^157^ *Tgfbr3* knockout mice exhibit coronary vessel formation failure and die by E14.5,^166^ which may be mediated by the inhibitory effect of TGF-β2 and BMP2 on epicardial cell invasion.^167^ In vitro application of TGF-β stimulates monolayer epicardial cells, inducing EMT characterized by morphological changes and increased EMT markers.^165,168,169^ BMP signaling also participates in proepicardial lineage determination. Myocardial-specific *Alk3* knockout in epicardial cells results in a markedly reduced AV groove and severely underdeveloped fibrous annulus, although epicardial and EPDC formation remain unaffected.^170^ Inhibiting BMP signaling can rescue epicardial maturation defects caused by *Wt1* knockout, suggesting that BMP signaling must be absent at specific stages for normal epicardial maturation.^171^

### Transcription factors

The normal development of the heart involves the participation of many TFs, including NKX2-5, GATA family proteins, MEF2 factors, T-box factors, and the Lim-homeodomain protein ISL1. These TFs interact with each other and, along with other signals, regulate heart development (Table 2). For instance, BMP4 regulates the expression of NKX2-5 through GATA4, and NKX2-5 can regulate JARID2 expression to control heart development.^9,18,172–174^

NKX2-5 is first expressed in embryonic heart progenitor cells and continues to be expressed during the embryonic, fetal, and adult stages, where it controls multiple aspects of heart development.^7^ NKX2-5 can act synergistically with other cardiac TFs to promote CM differentiation and chamber identity. For example, it collaborates with HAND2 and MEF2C to promote cardiac ventricle formation^175,176^ and with GATA4 to promote cardiomyocyte differentiation.^177^ Previous studies have shown that *Nkx2-5*^+^ cardiac progenitor cells (CPCs) are not only involved in the cardiomyocyte lineage but also contribute to cardiac endothelial cells and smooth muscle cells.^62,178^ Recent single-cell sequencing has shown that *Nkx2-5*-expressing progenitor cells rapidly differentiate into CMs, contributing only to the CM lineage, possibly because the single-cell analysis focused only on cells actively expressing *Nkx2-5*, excluding their derivatives that had already ceased *Nkx2-5* expression. One hypothesis is that the expression of *Nkx2-5* quickly ceases after these progenitor cells differentiate into stable endocardial or smooth muscle cells. At E8.5, *Nkx2-5*^+^ cells co-express multiple cardiomyocyte and smooth muscle cell markers, suggesting that this population has the potential to differentiate into both cardiomyocytes and smooth muscle cells. However, this may also reflect the known expression of smooth muscle genes in immature cardiomyocytes.^65,179^ *Nkx2-5*^+^ progenitor cells also participate in the formation of the pulmonary myocardium at the junction of the pulmonary veins and atria. Interestingly, atrial cells do not participate in this process, suggesting that the pulmonary myocardium may originate from pulmonary mesenchymal cells.^180^ Additionally, NKX2-5 is crucial for the differentiation of the cardiac Purkinje fiber network.^181,182^ Thus, defects in NKX2-5 manifest not only as various cardiac developmental abnormalities but also as cardiac conduction defects and arrhythmias.^183^

GATA4 also plays a crucial role in heart development. GATA4 deficiency leads to the failure of midline fusion of the heart primordia and extensive endoderm defects, resulting in embryonic death.^184,185^ A gradual reduction in GATA4 leads to abnormal heart development, including in the common AVC, DORV, and underdeveloped ventricular myocardium.^186^ Patients with deletions in the *GATA4* locus exhibit various cardiac developmental defects, including septation defects, OFT alignment defects, dextrocardia, and pulmonary stenosis (PS).^187,188^ This may be related to the interaction of GATA4 with other transcription factors, including NKX2-5, HAND2,^189,190^ TBX5,^191^ and ISL1.^192^ Combined defects in *Gata4* and *Tbx5* lead to complete atrioventricular septal defects (AVSDs), single atrioventricular valves, and myocardial thinning, while genetic interactions between *Gata6* and *Tbx5* can lead to neonatal lethality with thin myocardium.^191^ Mutations in *Gata4* disrupt the physical interaction between *Gata4* and *Tbx5*, leading to cardiac septal defects.^193^ These studies suggest the synergistic role of GATA4 and TBX5 in cardiac septum formation.

ISL1 is a LIM domain protein and a transcription factor that is transiently expressed during the appearance and expansion of SHFs and is downregulated as differentiation progresses.^194^ During development, ISL1 is expressed in the myocardial lineages of the distal OFT, atrial septum, and sinoatrial and atrioventricular nodes. It also contributes to the development of endothelial and vascular smooth muscle lineages, including the smooth muscle of coronary vessels.^195^ Further studies have shown that homeobox (*Hox*) genes (*Hoxa1*, *Hoxb1*, and *Hoxa3*) are temporarily expressed in early *Isl1* progenitor cells.^179^ These genes encode highly conserved homeodomain TFs that play roles in septation, CNCCs migration, and OFT development.^196^ *Isl1*-expressing progenitor cells undergo gradient changes in key gene modules to adopt their terminal cell fate, indicating multiple steps of fine-tuned orchestration, thereby promoting the development of the OFT and RV.^65,179^ Mice lacking *Isl1* show a complete absence of the OFT, RV, and most of the atria.^59^ Patients with heterozygous *ISL1* mutations exhibit DORV combined with VSD,^197^ highlighting the crucial role of ISL1 in cardiac development.

Paired-like homeodomain transcription factor 2 (PITX2) encodes a paired related homeodomain transcription factor essential for cardiac development. During cardiac development, PITX2 has two main functions: morphogenesis of the OFT and left-right specification of the atria. As a target of canonical Wnt signaling, PITX2 functions in both cardiac neural crest and mesoderm-derived SHF, regulating OFT myocardial proliferation and promoting OFT development in the SHF.^198,199^ In the left atrium, PITX2 determines left atrial morphology. Mutations in *Pitx2* result in left atrial characteristics, including venous valves and trabeculated myocardium, resembling those of the right side.^198^ *Pitx2* deficiency leads to AVC with prominent swelling and juxtaposition of the atrium, accompanied by undeveloped tricuspid and mitral valves and the formation of a common atrioventricular valve, indicating the role of Pitx2 in valve development as well.^200^ Single-cell sequencing revealed that *Pitx2* deficiency disrupts the differentiation dynamics of both anterior and posterior SHF-derived progenitor cells, preventing the activation of *Tgfb1* and *Hand1* in the OFT.^201^ Additionally, *Pitx2*-deficient embryos exhibit bilateral or ectopic sinoatrial nodes and defects in the pulmonary myocardial sleeve, which may explain the susceptibility to atrial fibrillation observed in adult animals with reduced PITX2 expression.^180,202,203^

Nuclear transcription factor Y subunit a (NFYa) is involved in regulating cell proliferation and metabolism. A recent study revealed that NFYa is expressed in the embryonic heart. Spatial and single-cell transcriptome sequencing revealed that *Nfya* deficiency leads to reduced CM proliferation and impaired mitochondrial metabolism, accompanied by a decrease in the number of immature regenerative cells and an increase in the number of trabecular and mature CMs. These effects are likely due to NFYa’s interaction with its cofactor SP2, which activates metabolism- and proliferation-related genes at the transcriptional level.^204^ However, research on the role of NFYa in heart development is still limited, and further studies are needed to explore its functions in this context.

HAND proteins are basic helix-loop-helix (bHLH) proteins that can form homo or heterodimers with bHLH partners, regulating gene expression. During mouse heart development, HAND factors are expressed in various or overlapping regions of CNCCs, the epicardium, the myocardium, and the endocardium.^205^ Early studies showed that HAND1 is primarily expressed in the left ventricle and is also expressed in CNCCs and the OFT, while HAND2 is strongly expressed in the endocardium and is also expressed in CNCCs, the OFT, and the epicardium. Mice lacking *Hand2* exhibit severe right ventricular hypoplasia, a thin myocardium, a dilated aortic sac, and VSD.^50,206–208^ Mice with a global knockout of *Hand1* die at E9.5 due to defects in extraembryonic tissues and cardiac morphology.^209^ Cardiac-specific *Hand1* deletion leads to defects in the left ventricle and endocardial cushions and dysregulation of ventricular gene expression, with embryos surviving to the perinatal stage but dying from various cardiac abnormalities.^210^

Further studies revealed that conditional loss of *Hand1* and *Hand2* in the left ventricle causes abnormal trabeculation and thickened compact myocardium by mediating abnormal proliferation and differentiation, highlighting the role of HAND factors in left ventricular development.^211^ Interestingly, transgenic embryos with *Hand2* expression throughout the ventricles show expanded boundaries between the left and right ventricles, with complete septal absence, indicating the critical importance of *Hand2* expression balance for ventricular morphology.^212^ Single-cell sequencing identified *Hand2* as a specifier of OFT cells but not RV cells. Temporal single-cell transcriptome analysis revealed that *Hand2* deletion results in an undifferentiated OFT myocardium, while the right ventricular myocardium, although differentiated, fails to migrate properly to the arterial pole or differentiate, accompanied by disrupted RA signaling and anterior-posterior patterning of cardiac progenitors.^213^ Using *Mef2c*-, *Tie2*-, or *Nfatc1*-Cre to delete *Hand2* specifically, researchers found that endocardial HANDs act downstream of the Notch endocardium-to-myocardium signaling pathway to regulate NRG1, leading to tricuspid atresia, double inlet left ventricle, and reduced ventricular trabeculation, suggesting that HAND2 plays a role in endocardial development.^208,214^ During epicardial development, HAND2 is downstream of HAND1, and HAND2 deficiency impairs epicardial EMT, preventing the normal formation of cardiac fibroblasts and coronary vessels, further emphasizing the role of HAND2 in epicardial development.^215^

*TBX5* mutations were first discovered in human Holt-Oram syndrome patients who had secondary atrial and ventricular septal defects and defects in the cardiac conduction system.^216^ *Tbx5*-deficient embryonic mice exhibit incomplete heart septation and conduction block, along with underdeveloped atria, while *Tbx5* overexpression inhibits ventricular maturation.^217^ TBX5 can physically interact with MEF2C to activate the expression of MYH6 in cardiomyocytes, and dual knockdown of *Tbx5* and *Mef2c* causes severe defects in heart tube looping.^218^ Previous research has highlighted the role of TBX5 in septation. TBX5-mediated Hh signaling is required in the SHF for atrial septation in mice.^219^ TBX5 expression at ventricular boundaries defines the location of muscular septum formation in avian hearts, with TBX5-misexpressing hearts showing ventricular septum formation issues, resulting in a single ventricle.^220,221^ Additionally, *Tbx5* and *Mef2c* exhibit genetic interactions during ventricular septum formation, producing muscular septal defects, and *Mef2c* co-regulates *Tbx5* target genes.^222^ Using *Mef2c*-anterior heart field (AHF)-Cre mice, researchers found that AHF *Mef2c*^*+*^ cells primarily contribute to the development of the OFT, RV, and septal endocardial and myocardial components.^223^ The entire murine left ventricle, including the left side of the septum, expresses *Tbx5*, and further analysis revealed a small group of cells in the intersectional lineage expressing both *Mef2c* and *Tbx5*.^224^ These results suggest that TBX5 and MEF2C play synergistic roles in cardiac development, particularly during septation.

Other T-box factors also play significant roles in heart development. TBX1 is expressed in a multilineage-primed population within the cardiopharyngeal mesoderm, regulating the progressive differentiation of these cells into anterior and posterior SHF cells in the posterior pharyngeal region.^225^ TBX1 also interacts with the [brahma-related gene 1 (BRG1)/brahma (BRM)-associated factor 60a] (Baf60a)/[switch (SWI)/sucrose non-fermentable (SNF)-related, matrix-associated, actin-dependent regulator of chromatin] (Smarc)-d1 subunit of a chromatin remodeling complex to regulate Wnt5a expression, influencing cardiac progenitor differentiation.^226^ *Tbx1* deficiency leads to DiGeorge syndrome with various cardiac defects, including reduced proliferation in the SHF, aortic arch patterning defects, and OFT anomalies.^227–229^ Genetic tracking using *Tbx2*-Cre alleles showed that myocardial cells of the free wall and base of the left ventricle are gradually added from *Tbx2*-expressing cells in the AVC, which downregulates *Tbx2* expression upon leaving the canal. TBX2 functionally suppresses the chamber program in the AVC.^230^ TBX2 and TBX3 coordinate with BMP2 to trigger cushion development and locally repress chamber differentiation during chamber differentiation, stimulating AVC myocardium and AV nodal phenotype development and coordinating heart development.^231^ TBX18, along with other TFs such as WT1 and transcription factor 21 (TCF21), is expressed in epicardial progenitor cells and contributes to epicardial development. These functions are crucial for epicardial EMT and the subsequent formation of coronary vessels and interstitial fibroblasts.^232–234^ Single-cell sequencing further confirmed the role of *Tbx18*^*+*^ cells in epicardial EMT and the development of fibroblasts and cardiomyocytes.^235^ The cardiogenic mesoderm contains an additional subset of *Tbx18*-expressing progenitors proposed to form the *Tbx18*^*+*^/*Nkx2-5*^*-*^ sinus venosus and PE.^236^ TBX20, which acts upstream of TBX2 in heart development, causes heart formation defects when deficient, including AVC constriction, OFT and RV hypoplasia, and reduced expression of NKX2-5, HAND1 and -2, and MEF2C.^237–239^

### WNT signaling

In addition to various GFs and TFs, multiple signaling pathways regulate heart development. Table 3 summarized the signaling pathways commonly involved in cardiac development. The WNT signaling pathway plays a crucial role in heart development, especially during the early stages.

**Table 3:** Signaling pathways involved in cardiac development

Signaling	Functions in cardiac development	Consequence of Loss of function	References
Wnt signaling	Involved in mesoderm induction and patterning; Involved in SHF progenitor cell patterning and expansion; Involved in the development of OFT; Involved in heart tube formation, looping and RV formation; Differentiation of cardiomyocytes; Involved in the proliferation of mesenchymal cells and endocardial cushion development; Involved in epicardial and valve development	OFT morphogenic defects; RV defects; Lack of heart tube; ASD; Impaired cardiac function; Abnormal coronary development	^140,249–282^
Retinoic acid signaling	Involved in mesoderm formation and induction; Involved in FHF, SHF development and heart looping; Involved in OFT, atria and sinus venosus development; Involved in myocardial trabeculation; Differentiation of pacemaker cells; Involved in epicardial development; Regulation of specification of atrial cells	Atrioventricular cushion defects; VSD; Conotruncal ridge defects; DORV; Persistent truncus arteriosus; Aorticopulmonary window; Hypoplasia of the atria and sinus venosus; Abnormal coronary development	^18,64,121,233,278^ ^285–311^
Notch signaling	Involved in left-right organizer; Inhibition of cardiac differentiation in the early phase; Involved in AVC, endocardial and valve development; Migration of CNCC; Regulation of proliferation of mesenchymal cells; Regulation of conduction system development; Involved in myocardial trabeculation; Involved in epicardial development	AVC deformities; Tricuspid atresia; Double inlet left ventricle; Reduced ventricular trabeculation; Bicuspid aortic valve; Right ventricular hypoplasia; Overriding aorta; VSD; HLHS; DORV; Hypoplastic atrioventricular node; Trabeculation defect; Abnormal coronary development	^88,286,315–348^
Hedgehog signaling	Involved in heart tube development; Differentiation of cardiomyocytes; Involved in pharyngeal arch development, cushion formation and atrial septation; Migration of CNCC; Involved in sinus node development	Arch arteries and outflow tract defects; hypoplasia of the atrium; AVC defects; Abnormal sinus node development	^352–358^
Hippo signaling	Involved in mesoderm induction and heart tube development; Regulation of proliferation of cardiac cells, including trabecular cardiomyocytes, endocardial cells, epicardial cells and endothelial cells	Thin ventricular walls; Abnormal heart size; Cardiac bifida; Endocardial cushion defects; Trabeculation defects; Abnormal coronary development	^390–397,399–407^

*ASD* atrial septal defect, *AVC* atrioventricular canal, *CNCC* cardiac neural crest cells, *DORV* double outlet right ventricle, *FHF* first heart field, *HLHS* hypoplastic left heart syndrome, *OFT* outflow tract, *RV* right ventricle, *SHF* second heart field, *VSD* ventricular septal defect

WNT was first discovered in mice in 1982 and was identified as a homolog of the *Drosophila wingless* gene in 1987.^240,241^ WNT, a secreted signaling molecule, includes 19 ligands that function through Frizzled (Fz) receptors.^242,243^ Although three distinct WNT signaling pathways (the canonical WNT/β-catenin, the noncanonical WNT/planar cell polarity and WNT/calcium pathways) operate independently, they interact to form a complex signaling network influencing every stage of cardiac development.^240^ WNT1, -2, -3, -3a, -8, and -8b primarily act through the canonical pathway, while WNT4, -5a, -5b, -6, -7a, and -11 primarily act through noncanonical pathways.^244^ When WNT binds to a seven-transmembrane (7TM) heterodimeric receptor complex containing Fz receptor 2 (Fz-2) or the frizzled and protein low-density lipoprotein receptor-related protein (LRP)-5/6, the canonical WNT pathway is activated, leading to Disheveled (Dsh) hyperphosphorylation, which inhibits glycogen synthase kinase-3β (GSK-3β) in vertebrates, causing β-catenin accumulation and translocation to the nucleus, where it affects the expression of development-related genes.^245^ The WNT/planar cell polarity pathway activates the c-Jun N-terminal kinase (JNK) and [rat sarcoma homologous (Rho)-associated protein kinase] (ROCK) kinases through interactions between Dsh proteins and Rho family GTPases.^246,247^ The WNT/calcium pathway relies on phospholipase C, which triggers the release of calcium ions into the cytoplasm, activating protein kinase C, calcium/calmodulin-dependent protein kinase II, and calcineurin.^248^

The WNT/β-catenin pathway is essential for mesoderm induction and patterning.^249–251^ Previous studies suggested that WNT signaling is necessary for the patterning and expansion of SHF progenitors by activating a series of FGFs, such as FGF3, FGF10, FGF16, and FGF20, which in turn activate FGF signaling.^252,253^ Ablation of β-catenin in *Isl1*-expressing progenitors disrupts heart development in multiple ways, causing defects in the expression of essential cardiac genes such as *Tbx2*, *Tbx3*, and *Pitx2*. Conversely, activation of β-catenin signaling in *Isl1*^+^ progenitors inhibits differentiation and causes morphogenic defects in the OFT.^254,255^ Conditional knockout of β-catenin in *Mesp1*^+^ cells disrupts cardiac looping and right ventricle formation, accompanied by the expansion of ISL1- and BMP4-expressing cells and the absence of the heart tube.^140^ Deletion of *Wnt2* and *Wnt11* similarly results in a reduction in posterior SHF progenitors and defects in the cardiac OFT and ventricular wall, respectively.^256–258^ Another study revealed that deletion of *Wnt5a* and *Wnt11* leads to a significant loss of SHF progenitors during heart development, accompanied by increased Wnt/β-catenin signaling.^259^ Overexpression of *Wnt5a* affects the deployment of SHFs, preventing them from entering the OFT and leading to OFT shortening. *Wnt5a* deletion results in a reduction in the inferior OFT myocardial wall and its derivative, subpulmonary myocardium, and fails to extend into the arterial and venous poles, causing both OFT and ASD.^260–262^ A recent study revealed that WNT11 restricts the WNT/β-catenin signaling pathway through caspase-mediated degradation, which is necessary for cardiomyocyte differentiation.^263^ Single-cell sequencing revealed a specific subset of proximal OFT progenitor cells expressing the WNT signal activator gene leucine-rich repeat-containing G protein-coupled receptor 5 (*LGR5*) in the human heart, which may promote the expansion of a transitional cell population to achieve correct arterial pole alignment.^264^ These findings indicate that WNT signaling plays a crucial yet complex role in SHF development.

Precise activation and inhibition of WNT signaling are key to regulating normal heart development. Research has further suggested that WNT signaling has a biphasic role in cardiac development. While activation of the WNT/β-catenin pathway is essential for early specification of mesoderm and cardiac progenitor cells, its activation at later stages inhibits heart development.^265,266^ The endoderm-derived WNT inhibitor Dickkopf (Dkk) can suppress WNT signaling and induce heart-specific gene expression in the posterior lateral plate mesoderm.^267^ This finding was corroborated by in vitro stem cell studies. During the early differentiation of human embryonic stem cells (hESCs) into human cardiomyocytes, WNT3 and WNT8A activate the canonical signaling pathway through Fz-7, promoting mesoderm induction and Brachyury expression. Subsequently, WNT5A/5B activates the WNT/planar cell polarity pathway through receptor tyrosine kinase-like orphan receptor 2 (ROR2), promoting MESP1 expression and differentiation of the cardiogenic mesoderm. Finally, WNT2, WNT5A/5B, and WNT11 activate the WNT/calcium pathway through F Fz-4 and Fz-6, working in conjunction with inhibition of the canonical pathway to promote CM differentiation.^268^ In hPSCs, temporal regulation of WNT signaling is critical for generating functional cardiomyocytes and pacemaker cells.^269–273^

WNT signaling also plays an important role in the endocardial cushion, valve, and epicardial development. At E12.5, WNT2 is expressed in the cushion mesenchyme, while WNT4 and WNT9B are mainly expressed in overlying endothelial cells. By E17.5, WNT3A and WNT7B are expressed in the atrioventricular and semilunar valves.^274^ Deletion of β-catenin leads to underdeveloped endocardial cushions with reduced mesenchymal cell proliferation.^275^ Overexpression of the WNT inhibitor Dkk1 prevents cushion formation.^276^ Moreover, *Tbx20* deficiency causes severe valve extension defects and impaired cardiac function in mice, which is potentially mediated by the WNT pathway.^277^ WNT5A and WNT9B are expressed in the epicardium.^278,279^ Epicardial-specific deletion of β-catenin results in lethality between E12.5 and birth, resulting in impaired coronary formation, subepicardial space expansion, weakened myocardial invasion, and severely thinned ventricular myocardium.^280,281^

Furthermore, in embryos lacking the epicardial transcription factor WT1, epicardial EMT is inhibited, which is mediated by weakened canonical and noncanonical WNT signaling.^279^ Deletion of β-catenin also disrupts adherens junctions and randomizes the mitotic spindle orientation of epicardial cells, thus impairing epicardial EMT.^282^ Overall, WNT signaling plays multiple roles in heart development, including the specification of cardiac mesoderm and subsequent differentiation and proliferation, serving as a critical regulatory mechanism throughout cardiac development.

### Retinoic acid

RA is synthesized from retinaldehyde through oxidation by retinaldehyde dehydrogenases (RALDH 1-3), with RALDH2 being the primary source of RA during embryonic development. RA enters the nucleus by binding to cellular RA binding proteins (CRABP) to regulate gene expression and can exert its effects by binding to retinoic acid receptors (RARs) and retinoic acid X receptors (RXRs).^283,284^ The critical role of RA in heart development has been established since early studies.^18^ RA is now known to play essential roles, from the early formation of anterior-posterior boundaries of the cardiac mesoderm to the development of the epicardium and the subsequent formation of normal cardiac morphology. RALDH2 is expressed in the posterior lateral plate mesoderm in mice at E7.5-E8.0, suggesting that caudal cardiac precursors are primarily exposed to RA.^285^ Subsequently, from E9.5-E12.5, RALDH2 expression gradually extends to the developing atria and epicardium.^64,286–288^ Deficiency of *Raldh2* in zebrafish leads to specific increases in cardiac precursor cells in the anterior lateral plate mesoderm, resulting in larger hearts with increased numbers of atrial and ventricular cardiomyocytes, indicating a crucial role for RA in restricting the cardiac precursor area in the anterior lateral plate mesoderm.^289,290^ Deletion of *Raldh2* at E8.0-E9.0, although after cardiac crescent formation, leads to downregulation of SHF-related genes at the pharyngeal level of the anterior SHF and posterior expansion.^291,292^ The LIM domain protein Ajuba inhibits ISL1 expression in an RA-dependent manner, thereby restricting SHF expansion.^293^

Furthermore, TBX1 in the anterior SHF antagonizes RA signaling, while the induction of TBX5 in the posterior SHF depends on RA signaling.^294^ Therefore, RA signaling plays crucial roles in the generation and maintenance of cardiac precursor cells in the anterior SHF and in delineating the boundary between the anterior and posterior SHF. Embryos with RXRa deficiency exhibit complex cardiac developmental defects, including VSD, atrioventricular cushion defects, and conotruncal ridge defects, with DORV, an aorticopulmonary window, and persistent truncus arteriosus (PTA).^295^ Deficiency of *Raldh2* results in incorrect heart looping, severe defects in atrial and sinus venosus development, impaired formation of ventricular trabeculae, and defects in OFT septation.^296^ Subsequent studies revealed that RA acts on ventricles through RXRa receptors in the epicardium.^278^ Similarly, a lack of RA signaling in zebrafish significantly increased differentiation of FHF cardiomyocytes, markedly decreased OFTs and disturbed pacemaker cell differentiation.^297^ Interestingly, excess RA also leads to cardiac developmental defects, particularly abnormal OFT development, indicating that proper regulation of RA signaling is crucial for normal OFT development.^298–300^

RALDH2 expression in the epicardium begins as early as in the anterior epicardium at E9.5. While RA signaling is not essential for the initial formation of the epicardium, it exerts a significant influence on its subsequent differentiation.^301^ Epicardial WT1 directly activates RA signaling to regulate the expression of platelet-derived growth factor receptor a (PDGFRa), thereby modulating epicardial EMT.^302^ RA induces the expression of TCF21 and FGF9 in the epicardium, promoting their differentiation into fibroblasts and participating in myocardial proliferation and ventricular expansion, respectively.^121,233^ Excess or reduced RA signaling disrupts epicardial EMT, resulting in reductions in ventricular coverage, coronary vessel density, altered vessel morphology, and impaired recruitment of epicardial-derived mural cells.^303^ Additionally, RA signaling in the epicardium is necessary for cytoskeletal rearrangements during epicardial EMT, thereby promoting the infiltration of EPDCs into the myocardium.^304^ Under physiological conditions, high levels of RA synergize with myocardial-derived vascular endothelial growth factor (VEGF) to inhibit EPDC differentiation into coronary smooth muscle cells, ensuring the establishment of an extensive endothelial network.^305^

In recent years, studies using stem cells as in vitro models have extensively investigated the role of RA in myocardial and epicardial differentiation. In mouse embryonic stem cells (mESCs), differentiation into atrial cardiomyocytes in vitro is observed with different concentrations of RA.^306^ In hESCs, RA treatment at the cardiac progenitor stage promotes differentiation into atrial cardiomyocytes.^307^ Furthermore, Devalla et al. reported that atrial differentiation depends on the stimulation of RA signaling shortly after the peak expression of MESP1 in the cardiac mesoderm stage, a process mediated by meis homeobox 2 (MEIS2) and antagonized by ISL1 to induce the expression of the atrial transcription factor nuclear receptor subfamily 2 group F member 1 (NR2F1).^308,309^ In the absence of RA or other late induction signals in vitro, default differentiation of MESP1^+^ cardiac mesodermal cells leads most cell lines generated with existing differentiation protocols toward the ventricular lineage. Inhibiting RA signaling during differentiation may direct cells toward a ventricular fate.^307,310^ In contrast to in vivo *findings*, in vitro studies have shown that RA treatment can guide hPSC differentiation into PE. RA treatment synergizes with BMP4, VEGF, and WNT to guide hPSC differentiation into pro-Epi-like and Epi-like cells.^284,310,311^

### Notch signaling

Notch proteins, including Notch1-4, are single-pass transmembrane receptors found in mammals. Their ligands are transmembrane proteins on adjacent cell surfaces, including Delta–Serrate–Lag family ligands such as Delta-like protein (DLL)-1, -3, and -4, as well as Jagged family ligands such as Jagged (JAG)-1 and -2. Upon ligand binding, the receptor-bound ligand is modified by the E3 ubiquitin-protein ligase mindbomb (MIB)-1, activating Notch signaling. Subsequently, the γ-secretase complex and disintegrin and metalloproteinase (ADAM) proteins cleave and release the Notch intracellular domain (NICD), allowing it to translocate into the nucleus. Inside the nucleus, the NICD binds to the recombination signal binding protein for the immunoglobulin kappa J region (RBPJ) and recruits the coactivator mastermind-like protein 1 (MAML1), releasing corepressors (Co-Rs). The hairy and enhancer of split (HES) and [hairy/enhancer-of-split related to tyrosine-arginine-proline-tryptophan (YRPW) motif] (HEY) families of basic helix–loop–helix transcription repressors are well-known Notch targets.^89,312–314^ Newly activated transcription complexes driven by Notch target genes promote normal heart development. Mutations in Notch signaling molecules can lead to various forms of cardiac developmental defects, including BAV, VSD, overriding aorta, hypoplastic left heart, and incomplete right ventricular development.^315–322^ Notch signaling begins to function early in cardiac development. Activation of Notch signaling in the cardiac mesoderm reduces myocardial gene expression in *Xenopus* embryos, accompanied by increased expression of mesocardium and pericardial roof genes, whereas inhibition of Notch signaling promotes cardiac differentiation.^323^ Activation of Notch signaling in early embryonic stages reduces cardiac mesodermal transcript expression,^324,325^ and this phenomenon of Notch signaling inhibiting cardiac differentiation early in differentiation is also observed in mESCs.^326–328^ A recent study revealed that noncanonical Notch signaling, which does not depend on the transcription factor RBPJ, can inhibit cardiac development by inducing damage to the SHF.^329^ Abnormal expression of *Notch1* driven by *Mesp1*-Cre leads to abnormal heart morphogenesis characterized by ventricular and AVC deformities.^330^ Defects in *Rbpj* and *Notch1* driven by *Mesp1*-Cre and *Isl1*-Cre, respectively, lead to incomplete RV development. In *Isl1*^+^ cells, *Notch1* promotes SHF differentiation by downregulating WNT/β-catenin signaling,^331^ while in *Mesp1*^+^ cells, Notch signaling similarly regulates SHF differentiation as an upstream signal of WNT and BMP.^332^ Defects in *Jag1* in the SHF lead to abnormalities in the mouse aortic arch and heart, accompanied by decreased expression of *Fgf8* and *Bmp4*, defective migration of CNCCs, and defects in EndMT within the OFT endocardium.^333^ These results indicate that Notch signaling plays a role early in cardiac development, coordinating the development of cardiac precursor cells in the cardiac mesoderm and interacting with signals such as FGF, BMP, and WNT to regulate SHF progenitor cell differentiation.

Notch signaling is crucial for valve system development. Notch is highly active in the AVC and OFT endocardial cushions, cooperating with TGF-β, BMP, and WNT signals to induce EndMT. Disruption of Notch signaling impairs EndMT, leading to valve malformations such as enlarged valve cusps, BAV, DORV, and septal defects.^330,334–340^ Furthermore, defects in Notch signaling lead to excessive interstitial cells and abnormal valve thickening, possibly mediated by apoptosis regulated by Notch-RBPJ signaling to modulate leaflet remodeling^341^ or by limiting interstitial cell proliferation mediated by the activation of heparin-binding EGF-like growth factor (HBEGF).^336^ Aberrant expression of *Notch1* using *Tie2*-Cre increases EndMT in AVC and ventricular endocardial cells but also leads to midgestation lethality with defects in angiogenic remodeling of embryonic and yolk sac vasculature, cardiac development, smooth muscle cell investment in vessels, and hematopoietic differentiation.^337,342^ In zebrafish, overexpression of *N1ICD* in endothelial cells leads to hypertrophic cardiac valves.^343^ Interestingly, Notch signaling also affects the development of the conduction system. Activation of Notch signaling results in fully penetrant accessory pathways and ventricular preexcitation, similar to what occurs in human Wolff‒Parkinson‒White syndrome, while inhibition of Notch signaling leads to hypoplastic AV nodes, specific loss of slow conduction cells expressing connexin-30.2, and loss of physiological atrioventricular conduction delay.^344^

Notch signaling also plays an important role in ventricular development. During the trabeculation stage, NOTCH1 is expressed in ventricular endocardial cells at the base of forming trabeculae, while its ligands DLL4 and JAG1 are expressed at the base of forming trabeculae and in myocardial cells forming trabeculae, respectively.^345,346^ Defects in DLL4 in the endocardium lead to impaired trabeculation and reduced expression of markers, including G protein-coupled receptor (*Gpr*)*-126*, *Bmp10*, *Efn-b2*, and *Nrg1*, resulting in trabecular formation disorders.^346^ Similarly, mutations in *Rbpj* and *Notch1* lead to reduced expression and signaling of EFNB2, NRG1, and BMP10, decreased myocardial proliferation, and embryonic lethality at E10.5.^345^ Thus, NOTCH1 plays an important upstream role in trabeculation by regulating the expression of EFNB2, NRG1, and BMP10.

Another study revealed that NOTCH1 signaling promotes ECM degradation, while NRG1 promotes myocardial ECM synthesis, with NRG1-mediated VRGFa regulation linking these two systems to promote trabecular remodeling and growth.^88^ Recent studies also suggest the importance of Notch in coronary development. Notch signaling is activated to varying degrees during the transition from the PE to the epicardium-coronary artery, and specific deletion of *Notch1* using *Wt1*-Cre leads to coronary artery differentiation disorders, thin myocardial walls, decreased myocardial cell proliferation, and reduced *Raldh2* expression.^286^ Defects in *Rbpj* in the epicardium lead to disturbances in EPDC differentiation into coronary smooth muscle cells, while conditionally activating Notch signaling leads to premature differentiation of epicardial cells into smooth muscle cells and prevents coronary artery vasculogenesis.^347^ Another study revealed that prior to primary coronary plexus formation, coronary arterial precursors are specified through Notch in venous sinuses, with subsequent arterial differentiation depending on the DLL4-JAG1-EFNB2 signaling cascade.^348^

### Hedgehog signaling and cilia

The Hh signaling pathway was first discovered in *Drosophila* and later identified in mice and humans with three Hedgehog genes: Sonic Hedgehog (Shh), Desert Hedgehog (Dhh), and Indian Hedgehog (Ihh).^349,350^ Among them, Shh has been found to be most strongly associated with heart development. The classical Shh signaling cascade involves the binding of Shh to its receptor, Patched-1 (PTCH1), which is located on the primary cilium membrane of the cell. Upon Shh binding, PTCH1 inhibits the transmembrane receptor Smoothened (SMO), promoting its accumulation in the primary cilium. This activation leads to the regulation of gene expression through glioma-associated (GLI) transcription factors.^351^ Both overexpression and downregulation of Hh pathway components can lead to cardiac developmental defects. In zebrafish, reduced Hh signaling results in defects in cardiac muscle cells, while increased Hh signaling leads to an excess of cardiac muscle cells, ensuring the appropriate number of myocardial progenitor cells during early cardiac development.^352^ Early expression of Shh occurs in the floor plate of the murine notochord, dorsal to the cardiac region, followed by the formation of a Shh gradient in the pharyngeal endodermal region and the ventral aspect of the cardiac region in developing embryos. After the initial formation and extension of the primitive heart tube, the Shh ligand is produced and secreted from the pharyngeal endoderm of branchial arches 3, 4, and 6.^353^ Loss of *Shh* results in widespread failure of pharyngeal arch development, leading to defects in the arch artery and OFT patterning, as well as abnormal migration of CNCCs, causing cardiac defects.^354^

Further studies confirmed that the Shh ligand produced by the endoderm is essential for CNCCs survival and filling of OFT cushions while also mediating signals from AHF myocardial cells to complete septation after cushion formation.^355^ Subsequent experiments also revealed that Shh is necessary for dorsal mesenchymal protrusion (DMP) and the formation of atrioventricular septation.^356,357^ Conditional deletion of *Smo* in the SHF impairs the development of the cardiac venous pole, resulting in hypoplasia of the atrium/inflow tract and bradycardia, accompanied by decreased expression of critical developmental genes in the inflow tract sinus node, as well as failure of EndMT in the atrioventricular cushion, suggesting its potential role in the development of the conduction system and valves.^358^

Compared with that of the Hh signaling pathway, the influence of cilia on cardiac development has been studied more deeply. Cilia are divided into motile, nodal, or primary types, with a 9 + 0 or 9 + 2 ultrastructural arrangement of axonemal microtubules. Many highly complex and tightly coordinated developmental signaling pathways in embryonic heart development depend on primary cilia, which are prominent antennae-like structures present in almost all eukaryotic cell types.^359,360^ The primary cilium is characterized by its core comprising an axoneme of nine microtubule doublets arranged around a central space devoid of microtubules.^361^ Intraflagellar transport (IFT) uses the microtubule doublet central core to transport proteins bidirectionally into and out of the cilium. In mammals, IFT utilizes kinesin family member 3A (KIF3A) for anterograde transport, while cytoplasmic dynein enables retrograde transport.^362^ Primary cilia not only regulate Hh signaling but also have close connections with calcium signaling,^363,364^ WNT signaling,^365^ TGF-β,^366^ and Notch signaling.^367^ These primary cilia are present in the endocardium, myocardium, and epicardium of the embryonic heart.^368,369^ The initial stage of cardiac left-right development is initiated within a transient ciliated epithelium known as the left-right organizer, where motile and primary cilia play a crucial role in establishing the left-right asymmetry of the body axis and proper placement and patterning of internal organs, including the heart.^370–372^ Defects in ciliary signaling result in severe congenital heart defects, including heterotaxy syndrome, AVSD, and transposition of the great arteries (TGA).^373–375^

In a study by Slough et al., cilia were found in the embryonic mouse heart from E9.5 to E12.5, and after *Kif3a* knockout, abnormalities in endocardial cushions and compact myocardium development were detected.^376^ Defects in polycystin (PKD)-2 and -1 located on primary cilia are associated with defects in cardiac development, including disturbances in ventricular and atrial septum formation, disorganization and thinning of myocardial walls, and DORV.^377,378^ In a globally reduced cilia model in the developing mouse heart, severe developmental defects were observed, including the formation of a single OFT, AVSD, an enlarged pericardial sac, dilated atrial chambers, and decreased expression of the Hh signaling molecules GLI1 and PTCH1, resulting in embryonic mice dying around E13.5.^379^ Deficiency in *Ift88* leads to ventricular dilation at E11.5 in mouse embryos, reduced myocardial trabeculation, and abnormal OFT development.^380^

Cilia plays a crucial role in valve development, and patients with ciliopathies often have cardiac valve defects.^381^ Multiple cilia genes have been found to be associated with AVSD. Furthermore, mutations in cilia motility-related genes lead to AVSD only in the presence of heterotaxy syndrome, whereas mutations in cilia signaling-related genes lead to AVSD regardless of whether there are left-right patterning defects, perhaps because AVSD caused by mutations in cilia-motility genes results from situs abnormalities and does not involve Hedgehog signaling in SHF. In contrast, mutations in cilia signaling-related genes disrupt Hedgehog signaling in SHF, leading to AVSDs with or without laterality defects.^382^ Nonciliated mouse embryonic endothelial cells with a mutation in *Tg737*/*Ift88* were found to induce EndMT under shear stress through TGF-β signaling.^383^ In another study, the absence of primary cilia led to the enlargement of mitral valve leaflets, manifested as the expansion of the ECM and histological damage.^369^ The expression of Dhh in the endocardium is necessary for cilium-induced activation of the T-lymphoma invasion and metastasis-inducing protein (TIAM)-1-Ras-related C3 botulinum toxin substrate (RAC)-1 axis, which in turn stimulates the ECM remodeling required for proper valve remodeling.^384^ Primary cilia are also expressed on aortic valve interstitial cells and are lost as these cells differentiate into fibroblast-like cells. Loss of *Ift88* results in primary cilium loss increased fibrotic ECM production, and the occurrence of BAV.^385^ In summary, these results highlight the importance of cilia in cardiac development, particularly in early left-right organizer and subsequent valve development, as well as their impact on the OFT and ventricle formation.

### Cell proliferation and Hippo signaling

Cell proliferation refers to the process by which cells multiply and increase in number and involves a series of events known as the cell cycle, which consists of distinct phases, such as the gap0 (G0) phase, gap1 (G1) phase, synthesis (S) phase, gap2 (G2) phase, and mitosis (M) phase. During heart development, cell proliferation plays a crucial role in the growth and formation of the heart. It involves the multiplication of cells, contributing to the expansion of different cardiac tissues, such as the myocardium, endocardium, and valves. In the early phases of cardiac development, upon the formation of the heart tube, the proliferation of primitive cardiomyocytes ceases. The heart tube experiences growth with the highly proliferative activity of SHF cells facilitated by WNT signaling.^80^ Subsequent growth of the embryonic heart primarily occurs through cell division in the ventricles and atrial chambers. As mentioned above, within the ventricles, trabeculae develop from the endocardial lining through highly proliferative activity mediated by Notch signaling. FGF signaling, IGF, and WNT signaling also participate in the proliferative expansion of EPDCs in the subepicardial myocardium. However, the proliferation rate of cardiomyocytes exhibits local variations within the developing heart, with the highest rates observed in the ballooning ventricles and decreased rates in distal parts of the OFT. The flow tract and atria are formed by highly proliferating cardiomyocytes.^386^ The Hippo pathway has emerged as a major regulator of the proliferation of differentiated cardiomyocytes during cardiac development,^387^ yet the proliferation of cardiomyocytes gradually diminishes with cardiac development.

The Hippo signaling pathway controls cardiac development through the regulation of cell proliferation, apoptosis, and cell fate determination. Activation of the mammalian Hippo signaling pathway begins with a kinase cascade involving sterile 20-like protein kinases (MST1/2) interacting with the adapter protein salvador (SALV), leading to phosphorylation of SALV, large tumor suppressor kinase (LATS)-1/2, and [monopolar spindle (Mps) one binder kinase activator-like 1] (MOB1). This results in phosphorylation, cytoplasmic retention, and degradation of the transcriptional coactivator Yes-associated protein (YAP) and the transcriptional coactivator with PDZ-binding motif (TAZ). YAP shuttles between the cytoplasm and nucleus, where it stimulates gene transcription. Inactivation of upstream kinases allows YAP and TAZ to enter the nucleus, where they interact with various transcription factors, including [transcription enhancer factor (TEA) domain family member 1-4 (Tead1-4)] and proline-proline-X-tyrosine (PPXY)-containing transcription factors [including p73, runt-related transcription factor (RUNX), erythroblastic oncogene B-B2 receptor tyrosine kinase 4 (ERBB4) cytoplasmic domain, and SMADs].^388,389^ In hPSCs, peroxisome proliferator-activated receptor gamma coactivator 1 (PGC1)/peroxisome proliferator-activated receptor α (PPARα) signaling promotes cardiomyocyte hypertrophy and contractility development via YAP1.^390^ Mutations in various genes in the Hippo pathway lead to defects in heart development, particularly causing abnormal cell proliferation states, thin ventricular walls, and abnormal heart size.^388,389,391^ In hESCs, loss of *YAP* has been reported to regulate cardiac mesoderm by influencing primitive streak differentiation in response to Activin.^392^ Embryonic stem cells with a double knockout of *MST1/MST2* can differentiate into mesodermal cells but are significantly affected by further differentiation into cardiac cells, likely due to the inhibition of ligands of noncanonical WNT signaling.^393^ In zebrafish, sphingosine-1-phosphate (S1P) signaling also regulates bilateral cardiac precursor cell migration toward the midline through YAP1-dependent endoderm survival, with disrupted migration of cardiac precursors resulting in cardiac bifida, highlighting the important role of the Hippo signaling pathway during early cardiac development.^394,395^ The Hippo pathway also determines the number of cardiac precursors from the SHF that form the venous pole of the heart tube, which further determines the size of the atria.^396^ During this process, increased epithelial tension during heart tube extension may promote YAP-mediated cell division and proliferation, thus facilitating heart tube extension.^397^ Because the most prominent phenotype of Hippo pathway dysfunction in *Drosophila* is changes in organ size,^398^ controlling heart size via the Hippo pathway has long been of interest to researchers. The application of *Nkx2.5*-Cre to *Salv* knockout mice resulted in excessive embryonic heart growth. Although the cardiomyocyte size was unaffected, there was increased cardiomyocyte proliferation, and the embryos died postmutation. *Mst1/2* and *Lats2* knockout embryos also exhibit similar phenotypes.^399^

Interestingly, researchers have also found that Hippo signaling negatively regulates WNT signaling and that β-catenin heterozygosity suppresses the Hippo cardiomyocyte overgrowth phenotype, indicating that an interaction between Hippo and WNT signaling limits CM proliferation and controls heart size.^399^ Furthermore, embryonic inactivation of *Yap1* leads to lethal myocardial hypoplasia and reduced cardiomyocyte proliferation, whereas activation of *Yap1* stimulates the proliferation of cardiomyocytes, particularly trabecular cardiomyocytes, accompanied by significant downregulation of the expression of the myocardial marker *Nppa*. The mechanism by which *Yap1* activation stimulates proliferation may involve the activation of IGF signaling in cardiomyocytes, leading to increased β-catenin abundance and the activation of the WNT pathway.^400,401^ Subsequent studies also revealed that defects in *Taz* lead to a decrease in the number of trabeculae in the heart, along with a disorganized cortical actin structure and abnormal cell-cell junctions.^402^

Recent studies have also suggested that Hippo plays a role in the development of the endocardium and epicardium. Conditional deletion of *Yap* in endothelial cells disrupts TGF-β-SMAD signaling, inhibiting EndMT and reducing endocardial cell proliferation, resulting in defective endocardial cushion development and embryonic lethality.^403^ Another study revealed that defects in endocardial YAP/TAZ suppress NRG1, leading to impaired myocardial growth and decreased ventricular wall thickness.^404^ The increase in myocardial chamber volume during development also promotes the nuclear localization of YAP1 and thereby promotes endocardial proliferation.^405^ Inhibition of YAP/TAZ also leads to impaired EMT in the epicardium, reduced proliferation of epicardial cells, and decreased differentiation into coronary endothelial cells, with embryos dying around E11.5-E12.5 due to cardiovascular insufficiency.^406^ Subsequent studies of epicardial-specific knockout of *Lats1/2* also revealed embryonic lethality at E15.5, accompanied by defects in coronary vessel remodeling, likely due to mutant cells persistently expressing epicardial markers and failing to activate fibroblast differentiation.^407^

### Epigenetic regulation

The genetic and epigenetic basis of most CHDs remains largely unknown. Interestingly, previous studies have found mutations in coding sequences in only a few CHD patients, while a high proportion of mutations have been identified in epigenetic regulatory factors, highlighting the importance of epigenetic regulation in cardiac development.^408,409^ Major epigenetic modifications include DNA modifications, histone modifications, chromatin structure changes, and regulation by noncoding RNAs (ncRNAs). These key molecules and processes of epigenetic modification have been extensively reviewed in other studies.^410,411^ Here, we primarily summarize their roles in cardiac development (Table 4).

**Table 4:** Epigenetic regulation involved in cardiac development

	Factor	Functions in cardiac development	Consequence of Loss of function	References
DNA methylation		Demethylation wave running through gene bodies of cardiomyocyte genes in embryonic cardiomyocytes; Involved in cardiomyocyte transcriptome	TOF; VSD	^413–416^
Chromatin remodeling factors	BRG1/BRM associated factor	Involved in left-right organizer; Involved in mesoderm induction; Regulation of proliferation of cardiomyocytes; Involved in trabecular, valve, and epicardial development; Expression of the cardiac and contractile gene expression	Heterotaxy; VSD; Thin ventricular wall; Abnormal cardiac function	^417–425^
Histone modifications	UTX	Involved in heart tube development; Differentiation of cardiac cells	Linear heart tube; Lack of chamber formation; Thin myocardial wall	^426^
	DOT1L	Regulation of gene expressions of cardiac cells		^427^
	Polycomb complexes	Differentiation of cardiac cells; Involved in heart tube and ventricular development; Regulation of proliferation of cardiomyocytes; Involved in endocardial cushions and trabecular development	VSD; AVC defects; Trabeculation defects	^428–434^
	HDACs	Involved in the development of ventricles, trabeculation and conduction system; Regulation of proliferation of cardiomyocytes	VSD; Thin myocardial wall; Right ventricular luminal obstruction; bradycardia	^435,436^
	NuRD	Involved in the development of ventricles and trabeculation; Regulation of proliferation of cardiomyocytes	Enlarged left ventricle; Trabeculation defects; Thin myocardial wall; VSD	^437–440^
miRNA	miR-335-3p/5p	Upregulate the expressions of mesodermal and cardiac genes		^459^
	miR-1	Induce cardiac progenitor cells to exit the cell cycle and differentiate into cardiomyocytes; Involved in the development of myocardial sarcomeres and the conduction system		^460,461,465–467^ ^471,472^
	miR-133	Inhibit the differentiation of ES cells into a cardiac fate		^460^
	miR-133a-1/miR-133a-2	Regulation of proliferation of cardiomyocytes; Differentiation of cardiomyocytes; Involved in the early development of the posterior cardiac tube segment	VSD	^468–470^
	miR-218	Involved in migration of cardiomyocytes and heart tube formation; Differentiation of cardiomyocytes		^477,478^
	miR-27b	Differentiation of cardiomyocytes		^479^
	miR-322/-503	Promote cardiac differentiation		^462^
	miRNA-17-92	Promote SHF myocardial differentiation	VSD; Pulmonary hypoplasia	^463,464^
	miR-499	Differentiation of cardiomyocytes;		^471,472^
	miR-143/miR-138/miR-21	Involved in the development of valve and chamber formation		^473–476^
	miR-302-367	Regulation of proliferation of cardiomyocytes	Thin myocardial wall; Septation defects	^480^
lncRNA	linc1405	Activate *Mesp1* in mesoderm specification		^450^
	Bvht	Involved in mesoderm induction		^453^
	CARMEN	Promote cardiac specification and differentiation of cardiac progenitor cells		^454^
	CARMA	Differentiation of cardiomyocytes		^492^
	Platr4	Involved in mesoderm specification		^455^
	novlnc6	Regulation of NKX2-5 and BMP10		^457^
	Moshe	Involved in SHF development		^458^
	Uph	Regulation of proliferation of cardiomyocytes		^485,486^
	uc.457/TUC40/uc.245/uc.167/uc.40	Regulation of proliferation of cardiomyocytes; Differentiation of cardiomyocytes	VSD	^487–491^
	BANCR	Regulation of cardiomyocyte migration	Increased heart size	^493^

*AVC* atrioventricular canal, *BANCR B-Raf* proto-oncogene serine/threonine kinase (BRAF)-activated noncoding RNA, *BMP* bone morphogenetic protein, *BRG1* Brahma-related gene 1, *BRM* Brahma, *Bvht* Braveheart, *CARMA* CARdiomyocyte Maturation-Associated, *CARMEN* cardiac mesoderm enhancer-associated non-coding RNA, *DOT1L* disruptor of telomeric silencing 1-like, *HDACs* histone deacetylases, *Mesp1* mesoderm posterior 1, *miR* *microRNA* Moshe myocardial skeletal and heart enhancer (SHE) enhancer, *NKX2-5 NK2* homeobox 5, *novlnc6* novel long non-coding RNA 6, *NuRD* nucleosome remodeling and deacetylase, *Platr4* plasma long noncoding RNA 4, *SHF* second heart field, *ToF* tetralogy of Fallot, *TUC40* transcript upregulated in cancer 40, *uc* ultraconserved element, *Uph* upper-hand, *UTX* ubiquitously transcribed tetratricopeptide repeat X chromosome, *VSD* ventricular septal defect

In developing heart DNA, there is a significant enrichment of differentially methylated genes related to muscle contraction and cardiomyopathies. Additionally, high methylation of the *GATA4* gene has been detected in fetuses with Down syndrome with or without congenital heart defects and in fetuses with isolated cardiac malformations.^412^ In myocardial biopsies of patients with ToF and VSD, a highly methylated novel developmental Cytosine-phosphate-Guanine (CpG) island was found in the synthesis of the cytochrome c oxidase (SCO)-2 promoter, which may contain binding sites for transcription factors involved in early cardiac development.^413^ These results suggest that the DNA methylation status of cardiac genes may be associated with congenital heart disease (CHD). Another study using DNA methylome analysis of purified E14.5 and neonatal cardiomyocytes revealed a wave of demethylation through the gene bodies of embryonic cardiomyocyte genes, followed by de novo DNA methylation, which shapes the epigenome of maturing cardiomyocytes after birth.^414^ Subsequent studies revealed that myocardial cell development is characterized by active CpG methylation and histone marks interacting in *cis*-regulatory and genic regions, thereby forming the myocardial cell transcriptome.^415^ Condorelli and colleagues mapped the hydroxymethylome of the embryonic heart and found that DNA methylation at 5-cytosine (5-mC) occurs at highly expressed genes as well as at distal regulatory regions with enhanced activity, correlating with gene transcription.^416^ These findings preliminarily suggest a role for DNA methylation in cardiac development, but the specific regulatory mechanisms involved require further study.

The role of BRG1/BRM-associated factor (BAF), a chromatin remodeling factor, in cardiac development has been extensively studied. In embryonic stem cells, BRG1 is crucial for regulating active and repressive chromatin states, especially by activating developmental enhancers during mesoderm lineage commitment, and its absence leads to failed mesodermal induction and cardiomyocyte differentiation.^417^ BRG1 also regulates CM proliferation by acting on BMP10 and cooperatively regulates fetal heart differentiation via histone deacetylase (HDAC) and [poly (ADP ribose) polymerase] (PARP).^418^ Other studies also indicate that BRG1 plays a role in the valve, trabeculation, and epicardial development, largely through the regulation of ECM signaling.^419–421^ The BAF complex subunit BAF60C interacts with TBX5, NKX2-5, and GATA4 and with myocardin (MYOCD), promoting the expression of contraction genes. Defects in *Baf60c* lead to cardiac morphological defects with significant cardiac dysfunction, impairing the proliferation and differentiation of SHF progenitors and CMs.^422,423^ A recent study using immunopurification with mass spectrometry revealed that BAF60C and BAF170, together with BRG1, coregulate gene expression in cardiac precursors, and their loss compromises the expression of myocardial genes in cardiomyocytes.^424^ The polybromo-associated BAF complex (PBAF)-specific subunit BAF180 regulates the promoters of RA target genes, and its loss leads to severe ventricular developmental defects.^425^

Histone modifications also play a regulatory role in cardiac development. Ubiquitously transcribed tetratricopeptide repeat X chromosome (UTX), a histone H3 lysine 27 (H3K27) demethylase, can be recruited to cardiac-specific enhancers and promote the recruitment of BRG1. Its deficiency can lead to defects in embryonic stem cell differentiation into cardiac cells and in mouse heart development.^426^ Through a genome-wide chromatin-immunoprecipitation DNA-sequencing approach, it was found that in cardiomyocytes, H3 methyltransferase disruptor of telomeric silencing 1-like (DOT1L) mediates enrichment of H3 lysine 79 (H3K79), which is enriched in genes expressed during cardiac differentiation and regulates their expression.^427^ Polycomb complexes (PRC1 and PRC2) are essential regulators of epigenetic gene silencing. PRC1 ubiquitinates histone H2A lysine 119 (H2AK119) through the Ring1 protein, while PRC2 methylates H3K27 through enhancer of zeste homolog (EZH)-1 and EZH-2. The PRC2 subunit EZH2 has been found to interact with GATA4 and directly methylate it at Lys 299, thereby attenuating its transcriptional activity.^428^ Polycomb complexes can regulate the differentiation of embryonic stem cells into mesodermal and cardiac cells.^429^ In zebrafish, mutations in the PRC1 subunit *Rnf2* lead to tubular heart morphology accompanied by the upregulation and ectopic expression of *Tbx2/3*, resulting in the downregulation of ventricular-specific genes such as *Myh6* and *Nppa*.^430^ Inactivation of another PRC1 subunit, *Rae28*, leads to defects in heart tube looping and ventricular development during embryonic heart development, accompanied by impaired *Nkx2-5* expression.^431^ Loss of *Ezh1* and *Ezh2* also results in severe cardiac developmental defects, including EndMT impairment, reduced myocardial proliferation, incomplete endocardial cushion development, VSD, and incomplete trabeculation of the ventricular cavity.^432–434^

Additionally, the loss of histone deacetylases (HDACs), particularly HDAC1, HDAC2, HDAC5, and HDAC9, also leads to defects in cardiac development. Loss of HDAC1 results in embryonic lethality at E9.5, while mice lacking HDAC2 can survive until birth but exhibit a range of cardiac defects, including right ventricular chamber obstruction, excessive proliferation and apoptosis of myocardial cells, and bradycardia.^435^ Compound mutations in *Hdac5* and *Hdac9* result in lethal VSD and thin-walled myocardium.^436^ The nucleosome remodeling and histone deacetylation (NuRD) complex couples ATP-dependent chromatin remodeling with histone deacetylation. ATP-dependent chromatin remodeling activity is mediated by chromodomain-helicase-DNA-binding protein 3 (CHD3) and CHD4, and mutations in *Chd4* can lead to left ventricular dilation, thinning of compact myocardium, and reduced myocardial trabeculation.^437^ During heart development, NuRD also interacts with TFs, such as a friend of GATA (FOG)-2,^438^ TBX5,^439^ and TBX20.^440^ Disruption of the interaction with FOG2 leads to defects in septum development, thinning of the ventricular myocardium, and reduced myocardial cell proliferation.^438^

Various histone methyltransferases also play roles in cardiac development, including Su(var)3-9, Enhancer-of-zeste, Trithorax (SET) and Myeloid-Nervy-DEAF-1 (MYND) domain containing 1 (Smyd1),^441^ Wolf-Hirschhorn syndrome candidate 1 (WHSC1),^442^ and lysine (K)-specific methyltransferase 2D (KMT2D).^443^ Their loss also leads to defects in cardiac development. In summary, the deficiency of various histone modification-related molecules has been associated with cardiac developmental defects, primarily through their impact on the expression of cardiac developmental genes. However, the detailed mechanisms of these effects require further investigation in future studies.

Noncoding RNAs, including long noncoding RNAs (lncRNAs, >200 nucleotides), microRNAs (miRNAs, up to 22 nucleotides), and circular RNAs (circRNAs, formed by 1-5 exons), are extensively studied ncRNAs. They serve as epigenetic regulators or modulate gene expression at the transcriptional or posttranscriptional level, thereby participating in the regulation of cardiac development. miRNAs regulate gene expression posttranscriptionally by silencing protein-coding and noncoding genes.^444^ LncRNAs regulate gene expression through various mechanisms, including DNA looping, the recruitment of chromatin modifiers and transcription factors, the ability to act as miRNA sponges, and the ability to influence mRNA splicing, translation, or degradation.^445^ CircRNAs can act as miRNA sponges to counteract miRNA-mediated repression and participate in the regulation of RNA-binding proteins.^446,447^ However, research on the role of circRNAs in cardiac development is still limited. Previous studies have reported the expression of various circRNAs during human embryonic heart development and differentiation into CMs from hESCs, but their specific functions require further investigation.^448,449^

During the early stages of cardiac development, ncRNAs participate in the regulation of the expression of the TFs Brachyury and eomesodermin (EOMES) in the mesoderm. For example, linc1405 mediates the activation of *Mesp1* in the specification of cardiac mesoderm.^450^ Several ncRNAs are coexpressed with cardiac precursor cells, and TFs, such as EOMES, ISL1, TBX5, and TBX2, but their specific roles require further investigation.^451,452^ Another lncRNA, Braveheart (Bvht), acts upstream of *Mesp1* during mouse embryonic stem cell differentiation and interacts with the PRC2 component suppressor of zeste 12 homolog (SUZ12) to promote mesoderm-to-cardiac fate development.^453^ Similarly, the lncRNA cardiac mesoderm enhancer-associated noncoding RNA (CARMEN) interacts with the PRC2 components SUZ12 and EZH2 as upstream regulators of the cardiac mesoderm-specific gene regulatory network, promoting the specification and differentiation of cardiac precursor cells.^454^ LncRNA plasma long noncoding RNA 4 (Platr4) interacts with the Hippo signaling pathway molecules *Yap* and *Tead4* to regulate cardiac mesodermal lineage differentiation.^455^ Belmonte and colleagues identified three new lncRNAs, namely, transcriptional endoplasmic reticulum retention regulating long noncoding RNA (TERMINATOR), abnormal limb identity enhancer noncoding RNA (ALIEN), and PUNISHER, in hPSCs. TERMINATOR specifically controls pluripotent stem cell properties, ALIEN impairs cardiovascular development, and PUNISHER compromises endothelial cell function.^456^ The lncRNA novel long noncoding RNA 6 (Novlnc6) has been found to regulate the expression of *Nkx2.5* and *Bmp10* during development.^457^ The lncRNA [myocardial skeletal and heart enhancer (SHE) enhancer] (Moshe), an upstream regulator of *Gata6*, participates in cardiac development. Its downregulation increases *Nkx2-5* and SHF lineage gene expression, indicating its role in the complex network of cardiac development.^458^ During hESC cardiac differentiation, miR-335-3p/5p activates the expression of WNT and TGFβ signaling pathways, upregulating *Gata4*, *Nkx2-5*, and troponin T (*Tnnt2*).^459^ MiR-1 and miR-133 play opposing roles in pluripotent embryonic stem cell differentiation: miR-1 promotes cardiac progenitor cell exit from the cell cycle and differentiation into cardiomyocytes by targeting the Notch ligand *Dll-1*, whereas miR-133 inhibits differentiation toward a cardiac fate.^460^ MiR-1 also promotes the differentiation of hESCs into CMs by suppressing the WNT and FGF signaling pathways.^461^

During heart tube looping, miR-322/-503, an X-chromosome miRNA cluster, is enriched in *Mesp1*^+^ cells and may drive cardiac muscle differentiation by targeting the RNA-binding protein CUG-binding protein Elav-like family member 1 (*Celf1*).^462^ In subsequent differentiation of the heart tube, BMP drives the miRNA-17-92 complex to inhibit the expression of *Isl1* and *Tbx1* during cardiac development, promoting SHF myocardial differentiation.^463^ Mice deficient in miRNA-17‒92 die shortly after birth due to pulmonary hypoplasia and VSD.^464^ MiR-1 affects cardiac morphogenesis, myocardial sarcomere and conduction system development, and cell cycle control.^465–467^ Double mutations in miR-133a-1 and miR-133a-2 result in lethal VSD, ectopic expression of cardiac smooth muscle genes, and abnormal myocardial cell proliferation in embryonic mice.^468^ MYOCD positively regulates the miR-1/133a cluster. Knock-out of miR-1-1/133a-2 and miR-1-2/133a-1 releases the transcriptional coactivator MYOCD, keeping embryonic cardiomyocytes in an immature state.^469^ Subsequent studies revealed that miR-133a and RA regulate the expression of RhoA and cell division cycle (CDC)-42 through a negative feedback mechanism, thereby controlling myocardial cell proliferation and the early development of the posterior cardiac tube segment.^470^ MiR-499 exhibits specific expression during cardiac differentiation. In silico analysis revealed that the predicted targets of miR-499 overlap significantly with cardiac-specific miRNAs. The overexpression of miRNA-499 and miRNA-1 promotes the upregulation of *MEF2C* during differentiation, suggesting potential roles for these miRNAs in cardiac development.^471^ In vitro studies have shown that overexpression of miR-1 and miR-499 promotes the differentiation of cardiac progenitor cells into CMs.^472^ Several other miRNAs, including miR-143, miR-138, and miR-21, have been found to be associated with ventricular morphogenesis and valve development.^473–476^ In zebrafish, knockdown of miR-218 leads to impaired cardiac cell migration and heart tube formation.^477^ Subsequent studies revealed that miR-218 promotes the migration of mESCs during differentiation into CMs and inhibits CM differentiation.^478^ MiR-27b shows significant cardiac muscle expression during heart development, with the transcription factor gene *Mef2c* being a target of miR-27b.^479^ MiR-302-367 is crucial for the proliferation of developing cardiac muscle cells; knockout of miR-302-367 results in thinning of the ventricular wall, abnormal ventricular septum development, and reduced myocardial cell proliferation, while overexpression increases myocardial cell proliferation but also restricts cardiac ejection function.^480^ Defects in the miRNA-processing enzyme Dicer lead to DORV with concurrent VSD.^481^

In recent studies using hPSCs, researchers identified 96 miRNAs that promote the proliferation of hPSC-derived cardiomyocytes. Sixty-seven of these miRNAs likely act on different components of the Hippo pathway; for instance, miR-520d-3p targets *LATS2* and *TEAD1*, and miR-590-3p targets *YAP* and *TEAD*, suggesting that these miRNAs converge redundantly on Hippo signaling to activate *YAP* and robustly sustain proliferation. Therefore, further studies are needed to confirm the roles of these miRNAs.^482^ Other studies have shown that miR-302d and miR-10b also promote the proliferation of hPSC-derived cardiomyocytes, but their significance in cardiac development requires further investigation.^483,484^

Several lncRNAs also play roles during cardiac development by regulating various cardiac development-specific molecules. The lncRNA upper-hand (Uph) plays a crucial role in regulating *Hand2* expression, thereby regulating cardiac muscle cell proliferation and coordinating the balanced development of cardiac cell lineages.^485,486^ The lncRNA ultraconserved element (uc).457 is differentially expressed in the hearts of patients with VSD and regulates the proliferation and differentiation of myocardial cells by suppressing the expression of the histone cell cycle regulation defective homologs A (*HIRA*), *NPPA*, *TNNT2*, and *MEF2C*.^487^ Interestingly, during the differentiation of P19 cells into CMs, the overexpression of long noncoding RNA transcript upregulated in cancer 40 (TUC40), uc.245, uc.167, and uc.40 in lncRNAs in tumors upregulated cancer cells inhibited the proliferation and differentiation of CMs, promoting cell apoptosis.^488–491^ A recently identified lncRNA named CARdiomyocyte Maturation-Associated lncRNA (CARMA) increases the expression of miR-1-1 and miR-133-a2, inhibits Notch signaling by regulating the expression of the key effector *RBPJ* and thus regulates the differentiation and maturation of cardiomyocytes.^492^ Another lncRNA, [B-Raf proto-oncogene, serine/threonine kinase (BRAF)-activated noncoding RNA] (BANCR), is exclusively present in fetal chimpanzee cardiomyocytes and regulates CM migration through TEAD/YAP signaling. Deletion of BANCR in mouse models results in increased heart size, indicating its potential impact on cardiac development, which requires further investigation.^493^

### Embryonic microenvironment

With a deeper understanding of heart development, researchers have recognized the critical role of the embryonic microenvironment in this process. This unique milieu provides the foundation for normal cardiac development and intercellular signaling. Here, we focus on the effects of changes in the ECM, hypoxia, and metabolic environment on heart development from a broader perspective.

Although initially viewed as a relatively inert scaffold providing structural support to cells in their environment, the ECM is now widely recognized for its dynamic and plastic role in facilitating cell signaling, proliferation, and differentiation within the body, particularly during embryogenesis.^494,495^ Spatially, the ECM is primarily divided into the basement membrane/pericellular matrix and the interstitial matrix. The former is composed of fibronectin, collagen IV, laminin, procollagens, hyaluronic acid (HA), and proteoglycans, which promote signal transduction through cell surface receptors. The latter consists mainly of collagens I and III, providing tissue with structural and mechanical support.^496^ Throughout various stages of heart development, the role of ECM macromolecules is evident. During early development, the primitive heart ECM comprises chondroitin sulfate, collagens I and IV, laminin, fibulin, fibrillin, and fibronectin, which contribute to the migration of cardiac precursors toward the embryo midline.^18,495,497^ Fibronectin deposition at the midline is essential for heart development;^498^ its inactivation results in severe defects in mesodermally derived tissues, including cardiac development and early embryo death.^499,500^ After the primitive heart tube is formed, the cardiac jelly separates the endocardium and myocardium and contains various ECM molecules. The subsequent accumulation of ECM at the AV junction is crucial for the local swelling of cardiac jelly, which is essential for AV cushion formation. In zebrafish, knocking down the ECM protein Nephronectin limits AVC differentiation and cardiac jelly expansion by suppressing BMP4-hyaluronan-synthase 2 (HAS2) signaling, thereby preventing leaflet formation and trabeculation.^501^ The cardiac cushion ECM is rich in HA, and its disruption by hyaluronidase treatment leads to abnormal formation of endocardial cushions, thinning of the ventricular myocardial wall, and changes in ventricular function.^502^ Defects in uridine 5’-diphosphate (UDP)-glucose dehydrogenase, which is required for HA production, similarly lead to malformed AV cushions and valve formation issues.^503^ Embryos with defects in Hyaluronan-synthase 2 display similar outcomes, including myocardial thinning and impaired atrioventricular cushion development, while endocardial ERBB2-ERBB3 receptors may mediate the role of HA in valve and septum development.^504,505^ Other signals, such as BMP2 and TGF-β3, regulate heart AV cushion and valve development through the versican/HA and periostin/collagen pathways, respectively.^506,507^ Studies using gene knockout mouse models also highlight the essential role of periostin in septal and valve development.^508,509^

Furthermore, precise temporal and spatial control of ECM synthesis and degradation is crucial for the formation of trabeculations in the heart. Mutations in *Has2* and *Versican* can lead to defects in trabeculation.^505,510^ The expression of matrix metalloproteinase A disintegrin and metalloproteinase with thrombospondin motifs (ADAMTS)-1 prevents excessive trabeculation.^419^ Notch regulates ECM synthesis and degradation through ECM proteases and NRG1, promoting ECM remodeling during trabeculation development and thus facilitating normal trabeculation.^88^ The matrix metalloproteinase ADAMTS9 has also been found to participate in myocardial compaction. ADAMTS9 is expressed during development in the SHF, vascular smooth muscle cells in the arterial wall, mesenchymal cells of the valves, and non-myocardial cells of the ventricular myocardium. Loss of *Adamts9* results in abnormal valve development and abnormal myocardial projections, as well as a ‘spongy’ myocardium consistent with noncompaction of the left ventricle.^511^ During embryonic development, cardiac fibroblasts secrete ECM components, including fibronectin, collagen, and hyaluronic acid, promoting myocardial cell proliferation.^512^ In recent research, mouse embryonic stem cell-derived embryoid bodies were shown to generate heart organoids through the action of laminin-entactin (LN/ET) complexes and FGF4, which contains cardiac muscle, conducting tissues, smooth muscle, and endothelial cells capable of myocardial contraction and action potentials,^513^ emphasizing the importance of the ECM in heart development.

Fetal development occurs under conditions of relative hypoxia compared to adult oxygen tension. A low-oxygen environment during cardiac development is crucial for normal heart formation and maturation. A previous study indicated an increase in hypoxia staining and the accumulation of hypoxia-inducible factor 1 (HIF-1) during the peak of cardiomyocyte apoptosis in the OFT, a process essential for OFT remodeling. This finding suggested that hypoxia plays a significant role in embryonic OFT remodeling.^514,515^ Hypoxia also contributes to coronary vessel formation through VEGF during heart development.^234,516^ HIF-1, which is composed of α and β subunits, plays a significant role in hypoxia-dependent signaling, providing new insights into the role of hypoxia in heart development. In mice, hypoxic regions in the fetal heart are observed throughout embryonic development, with HIF stabilization correlating with these areas.^517^ The absence of HIF-1α leads to cardia bifida, abnormal cardiac looping, and cephalic blood vessels caused by reduced expression of MEF2C and HAND1 and defective CNCCs migration.^518^ Deletion of HIF-1β also results in embryonic lethality with placental and heart defects.^519^ Moreover, tissue-specific conditional knockout technology has been utilized to explore the role of HIF genes in heart development. Conditional knockout of HIF-1α in the embryonic mesoderm results in heart abnormalities, including the overriding aorta, ectopia cordis, and incomplete septation.^520,521^

While low oxygen conditions during fetal development are essential for embryonic heart growth, pathophysiological hypoxia can adversely affect cardiogenesis. Insufficient oxygen in utero leads to myocardial thinning, ventricle dilation, epicardium detachment, and a decrease in fetal heart maturation in both chicken and mouse models.^522,523^ Another study utilizing proteomics and metabolomics demonstrated that antenatal hypoxia alters pathways related to energy metabolism, lipid metabolism, oxidative stress, and inflammation and leads to mitochondrial reprogramming.^524^ The prevalence of CHD has been reported to be ten times greater in individuals living at high altitudes and exposed to chronic hypoxia than in those living at sea level.^525^ However, the intricate mechanism through which hypoxia regulates heart development requires further exploration.

**Fig. 4:**
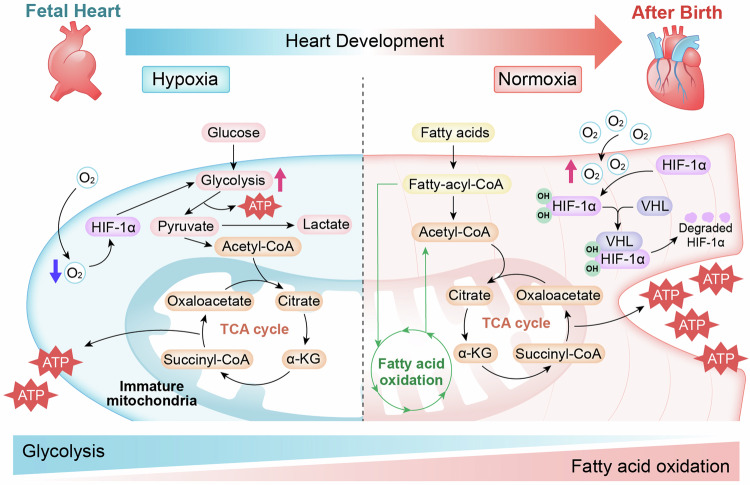
The role of hypoxia and metabolic transition during cardiac development. Significant changes in metabolic pathways accompany the maturation of cardiac development. During embryonic cardiac development, glucose and lactate serve as the primary sources of ATP. Hypoxic conditions activate HIF-1α during embryonic development, coupled with mitochondrial immaturity, which promotes a reliance on glycolysis for ATP production in the fetal heart, leading to increased lactate production. As cardiac development progresses, there is a simultaneous decrease in glycolysis and an increase in fatty acid β-oxidation during myocardial maturation. After birth, cardiac metabolism primarily relies on fatty acid oxidation, facilitated by an oxygen-rich environment. In this environment, the HIF-1α subunit is hydroxylated and targeted for degradation by VHL, thereby inhibiting its promotion of glycolysis. α-KG α-ketoglutarate, ATP adenosine triphosphate, CoA coenzyme A, HIF-1α hypoxia-inducible factor 1-alpha, OH hydroxide, O_2_ oxygen, TCA tricarboxylic acid, VHL von Hippel Lindau. This figure was created using Adobe Illustrator

During heart development, the embryonic heart undergoes significant changes in its metabolic environment. Due to the low levels of circulating free fatty acids in the fetal bloodstream, glucose and lactate serve as the main sources of ATP during early heart development and play crucial roles in cell proliferation. A decrease in glycolysis and a concurrent increase in fatty acid β-oxidation occur during myocardial maturation.^526^ However, due to the hypoxic environment, the embryonic heart continues to rely primarily on glycolysis rather than oxidative phosphorylation (OXPHOS) for ATP generation (Fig. 4).^527^ Furthermore, cardiomyocytes continue to depend on glycolysis and lactate oxidation for ATP production after birth, with a significant increase in fatty acid β-oxidation efficiency in subsequent days.^528^ A recent proteomic study in mouse embryos highlighted the significant upregulation of metabolic pathway-related proteins, including proteins involved in glycolysis, fatty acid oxidation, and OXPHOS, during late embryonic heart development (E13.5-E16.5). The mevalonate pathway also plays a critical role in regulating CM proliferation during heart development.^529^ The precise regulation of these metabolic transitions and their roles in fetal growth are increasingly being studied.

The Warburg effect refers to a metabolic state in which cells exhibit high glucose uptake and ferment glucose into lactate despite the presence of adequate oxygen and functional mitochondria. However, fetal cellular metabolism does not conform to the Warburg effect because lactate is actively consumed, and the intrauterine environment is relatively hypoxic. Transient exposure to lactate has been shown to induce significant and enduring transcriptional alterations in hPSC-derived cardiomyocytes, including the inhibition of WNT signaling, reduced proliferation, and elevated gene expression related to cardiac contractility and calcium signaling, suggesting that lactate may act not only as a metabolic waste product but also as a regulator of molecular signals during development.^530^ However, another study demonstrated that deleting *Hif1α* inhibited glycolytic activity but did not affect heart differentiation,^531^ possibly because metabolic products from other pathways during development compensate for the effects of glycolytic inhibition and because the impact of lactate on heart development may be dose-dependent. In 2019, Zhao et al. reported that lactate can lactylate histone lysine residues, a modification implicated in various physiological and pathological processes.^532^ Interestingly, lactylation has been linked to signaling pathways crucial for heart development, such as the WNT pathway,^533^ Hippo pathway,^534^ and Notch pathway,^535^ suggesting its potential importance in cardiac development. However, there is currently no research specifically addressing the role of lactylation in heart development, and further studies are needed to elucidate the effects of lactate in this context.

Diabetes is a risk factor for CHD. During normal embryonic development, glucose intake in the developing heart gradually decreases in late pregnancy. Disruption of glucose metabolism during diabetic pregnancies negatively affects the maturation of cardiomyocytes in the fetal heart. High glucose enhances nucleotide biosynthesis via the pentose phosphate pathway in hPSC-derived CMs.^536^ Mature cardiomyocytes adaptively rely on fatty acid β-oxidation (FAO) for ATP production. Recent studies in hPSC-derived cardiomyocytes suggest that fatty acids promote cardiomyocyte maturation by stimulating mitochondrial biogenesis, enhancing oxidative metabolism closer to adult ventricular tissue transcriptional characteristics, increasing muscle fiber density and arrangement, improving calcium handling, enhancing contractility, and enhancing physiological action potential dynamics.^537–541^ Activation of PPAR signaling pathways associated with fatty acid maturation processes similarly promotes hPSC-derived cardiomyocyte maturation.^530,542,543^ However, the current understanding of how metabolic environments influence heart development is limited primarily to pluripotent stem cell models, and in vitro differentiation protocols for pluripotent stem cells are still immature and rely on empirical evidence. Thus, insights gained from in vitro models regarding gene-environment interactions need validation in animal models.

### Advancements in understanding the molecular mechanisms of cardiac development

Autophagy is an intracellular process that generates double-membrane-bound vesicles called autophagosomes in the cytoplasm, which transport substrates to lysosomes for degradation. Recent studies have suggested that autophagy is involved in cardiac development by promoting the differentiation of myocardial cells and that the loss of autophagy can lead to defects in heart development. Autophagy plays an important role in regulating the pluripotency of embryonic stem cells.^544^ The activation of autophagy on days 0–4 of hPSC-derived cardiomyocyte differentiation decreased the expression of CM-specific genes, whereas the activation of autophagy on days 4–6 achieved the greatest CM differentiation efficiency by inhibiting the WNT signaling pathway, indicating that autophagy regulates CM differentiation in a stage-dependent manner. One possible explanation is that autophagy directly degrades cytoplasmic β-catenin, leading to the suppression of WNT signaling in the early stages of cardiomyocyte differentiation, while WNT activation is essential for the early phase of differentiation.^545^ Knockdown of genes required for the autophagic process led to a small, string-like heart with pericardial edema in zebrafish, with defects during the heart jogging process followed by imperfect cardiac looping.^546^ Moreover, the knockdown of core autophagy genes, including *Atg5*, *Atg7*, and *Becn1*, also causes defects in morphogenesis and abnormal heart structure in zebrafish.^547^

The development of single-cell RNA sequencing and single-nucleus RNA sequencing has uncovered the complex cell-to-cell communication and interactions between cardiomyocytes and numerous non-cardiomyocytes, which are crucial for normal heart development. Non-cardiomyocytes account for approximately 40%-70% of the total cells in the human heart, with the exact proportion varying across different studies, possibly due to differences in detection methods and sample types.^548^ Single-cell analysis of mouse hearts from embryonic days E9.5 to E18.5 identified eight different cell types, including cardiomyocytes, myoblasts, endothelial cells, vascular smooth muscle cells and pericytes, fibroblasts and myofibroblasts, T cells, macrophages, monocytes, and dendritic cells. ^549^ Another study analyzing the developing human heart [between 9 and 16 post-conception weeks (PCW)] identified cell types such as various subpopulations of cardiomyocytes, mesenchymal cells (epicardial cells, fibroblast-like vascular smooth muscle cells, and pericytes), endothelial cells (blood endothelial cells, lymphatic endothelial cells, and endocardial cells), blood cells (macrophages, white blood cells, and platelet-red blood cells), and neuronal cells (Schwann cells and neural crest cells).^550^ Further analysis revealed that Semaphorin (SEMA)-3C, secreted by ventricular fibroblasts, may attract Plexin A (*PLXNA2/4*)^+^ trabecular ventricular cardiomyocytes to migrate into the compact layer. Additionally, SEMA6A and SEMA6B in endothelial cells may compete with SEMA3C to prevent the further migration of trabecular ventricular cardiomyocytes within the compact layer. This theory is supported by the observation that specific deletion of the *Sema3c* gene in mouse cardiac fibroblasts leads to non-compacted, hypertrabeculated cardiac ventricles.^550^ In another study, Hou et al. used single-cell sequencing to identify 11 cell types in the developing human heart (at 8, 10, 11, and 17 PCW). CellChat analysis of intercellular communication revealed that at 8 PCW, the interaction strength between cell types was relatively low, but it significantly increased from 10 to 17 PCW. Furthermore, the interaction strength of the oncostatin M (OSM) signaling pathway gradually decreased during this period (from 8 to 17 PCW), while signaling pathways such as macrophage migration inhibitory factor (MIF), Pleiotrophin (PTN), and Granulin (GRN) became more complex, with increased cell-to-cell communication at 10, 11, and 17 PCW.^551^ Our previous research also explored cell communication between epicardial cells and other cardiac cells during mouse heart development. We found that epicardial cells interact with various cell types via ligand-receptor interactions. For example, they interact with immune cells through tumor necrosis factor (TNF)-Fms-related tyrosine kinase 4 (FLT4) and cluster of differentiation (CD)-44-FGFR2, with cardiomyocytes through CD74-MIF, and with fibroblast-like cells through CD46-JAG1.^552^ Although new technologies have provided vast amounts of information on the communication and interactions between different cell types during cardiac development, further validation is still required to clarify the specific roles of these interactions within complex signal network.

In recent years, the role of cardiac macrophages has gradually gained increased attention. Although recent single-cell studies have identified macrophages in the developing heart,^549–551^ their role in cardiac development remains largely unknown. Cardiac macrophages generally originate from two distinct sources: self-sustaining and renewable macrophages established during the embryonic stage, and macrophages derived from monocytes that migrate into tissues. The former makes up the majority of immune cells in the heart and is primarily active in development and homeostasis, where it functions in clearing apoptotic cells, regulating cardiomyocyte proliferation and angiogenesis, removing damaged mitochondria, and promoting electrical conduction. The latter is primarily associated with pathological conditions.^553,554^ Currently, through the use of cell surface markers [e.g., chemokine receptor-2 (CCR2); lymphatic vessel endothelial hyaluronan receptor 1 (LYVE1); and T-cell immunoglobulin and mucin domain-containing 4 (TIMD4)], genetic fate mapping, transcriptomic analysis, and functional studies, various subtypes of tissue-resident cardiac macrophages have been successfully identified and classified.^553^ In the mouse heart, three transcriptionally distinct macrophage subpopulations have been identified: TLF^+^ macrophages [expressing TIMD4, LYVE1, and/or folate receptor beta 2 (FOLR2)] maintained through self-renewal, CCR2^+^ macrophages dependent on monocytes [CCR2^+^ (TIMD4^−^LYVE1^−^FOLR2^−^)], and partially monocyte-dependent major histocompatibility complex (MHC-II)^hi^ macrophages (TIMD4^−^LYVE1^−^FOLR2^−^CCR2^−^).^555^ Recent studies have revealed that these macrophages play critical roles in various cardiac diseases, such as myocardial injury,^556,557^ atherosclerosis,^558^ and myocarditis,^559^ though these aspects are not elaborated on here. Macrophages first appear in the yolk sac between E6.5 and E8.5, and these yolk sac-derived macrophages colonize the heart and persist as TLF^+^ and MHC-II^hi^ macrophages into adulthood.^560^ Between E12.5 and E17.5, the heart primarily receives macrophages from the fetal liver, including TLF^+^, MHC-II^hi^, and CCR2^+^ macrophages. This occurs as hematopoietic stem cells migrate from the aorta-gonad-mesonephros (AGM) to the fetal liver around E10.5, making the fetal liver the primary hematopoietic site for macrophage production. From E16.5 onwards, hematopoietic stem cells colonize the developing bone marrow, and mature monocytes migrating into tissues differentiate into macrophages to replenish multiple cardiac macrophage lineages, primarily generating CCR2^+^ macrophages and minimally contributing to other subsets.^554,560^Other classification systems for cardiac macrophages include LYVE1^hi^MHCII^lo^ and LYVE1^lo^MHCII^hi^ macrophages,^561^ as well as yolk sac/AGM-derived macrophages [Chemokine (C-X3-C motif) receptor 1 (Cx3Cr1)^+^ Colony stimulating Factor 1 Receptor (Csf1r)^+^F4/80^+^), fetal liver monocytes (F4/80^lo^CD11b^hi^), and monocyte-derived macrophages (CCR2^+^MHC-II^hi^).^562^ Among these, LYVE1^hi^MHCII^lo^ macrophages, Cx3Cr1^+^Csf1r^+^F4/80^+^ macrophages, and F4/80^lo^CD11b^hi^ macrophages are similar to the TLF^+^ macrophages described earlier. The emergence of different classification methods reflects the complex spatiotemporal heterogeneity of cardiac macrophages, where different macrophage phenotypes may be expressed at various stages of heart development, or even macrophages with identical phenotypes may originate from different sources. Further research is needed to elucidate the ontogeny and functions of cardiac macrophages.

**Fig. 5:**
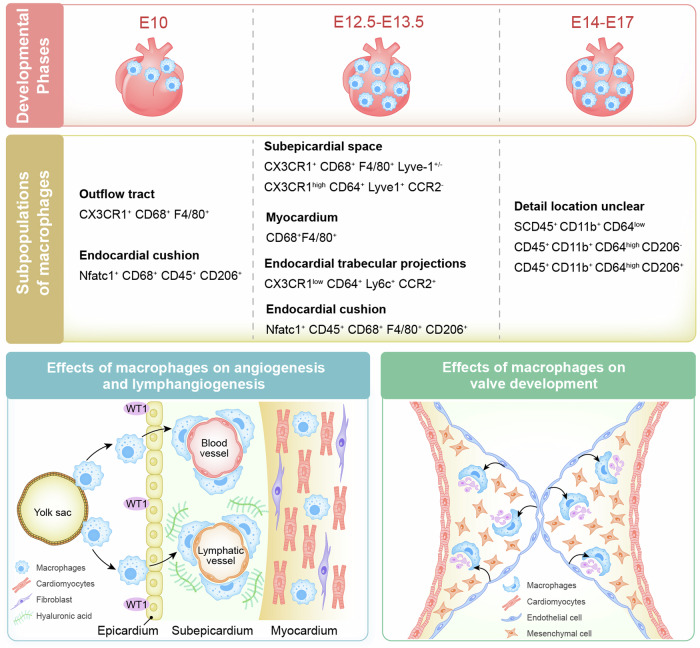
The role of macrophages during cardiac development. Macrophages first appear during mouse embryo development around E10 near the outflow tract, sinus node, and endocardial cushions. As the heart matures, they are distributed beneath the epicardium, within the myocardial layer, and in the endocardial layer throughout the entire heart. Various macrophage subpopulations have been identified at different times and locations, with significant research focused on subepicardial space subpopulations (CX3CR1^+^ CD68^+^ F4/80^+^ Lyve-1^+/-^; CX3CR1high CD64^+^ Lyve-1^+^ CCR2^-^). These macrophages originate from the yolk sac and migrate to the epicardium under the influence of the transcription factor WT1, subsequently entering the subepicardial space. In the subepicardial space, these macrophages adhere to the endothelial cells of blood vessels, promoting the normal development of coronary arteries. Simultaneously, they adhere to the endothelial cells of lymphatic vessels and facilitate normal lymphatic development through direct interactions involving hyaluronic acid. Macrophages within endocardial cushions (Nfatc1^+^ CD45^+^ CD68^+^ F4/80^+^ CD206^+^), derived from endothelial cells, likely contribute to valve development through phagocytic activities. CCR2 C-C chemokine receptor type 2, CX3CR1 chemokine (C-X3-C motif) receptor 1, Lyve-1 lymphatic vessel endothelial hyaluronan receptor 1, Nfatc nuclear factor of activated T cells, cytoplasmic, WT1 Wilms tumor 1. This figure was created using Adobe Illustrator

Currently, embryonic cardiac macrophages can be first observed near the OFT around E10, increasing up to E14.5, predominantly on the surface of the heart in the subepicardial space. These cells play crucial roles in vascular and lymphatic vessel formation, as well as valve development (Fig. 5).^560,562–564^ Yolk sac-derived CCR2^−^ macrophages are essential for the remodeling of the primitive coronary plexus, and IGF signaling potentially mediates the proangiogenic properties of embryonic-derived macrophages.^564^ Two other studies also revealed substantial numbers of embryonic tissue macrophages (phenotypes CD68^+^, F4/80^+^, CD206^+^, Lyve-1^+^, and Cx3cr1^+^) adhering to vascular and lymphatic walls around E12.5, with macrophages interacting directly with lymphatic endothelial cells via hyaluronic acid. Subsequently, between E14 and E17, cardiac macrophages can be classified into three subgroups expressing genes associated with vascular and lymphatic vessel formation and ECM remodeling.^565,566^ Shigeta and colleagues further discovered macrophages originating from endocardial cushion-associated hemogenic endothelial precursors in the embryonic heart, suggesting that these cells play an important role in valve development through phagocytic activity.^563^ In vitro studies have shown that hPSC-derived primitive yolk sac-like macrophages (CCR2^−^, LYVE1^+^, CD45^−^, CD14^+^, CD64^+^, and CD68^+^) cocultured with a tri-culture of endothelial cells, dental pulp stem cells, and CMs also localize around blood vessels and promote higher vessel density, junction number, and length, while reducing mean lacunarity. In the Biowire heart-on-chip platform, macrophage coculture resulted in denser and more intact ECM, highly dense and striated CMs, higher contractile force, and a greater excitation threshold. These effects may be attributed to the increased secretion of pro-angiogenic cytokines, cardioprotective upregulated cytokines, adiponectin, and the upregulation of genes involved in cardiac maturation and angiogenesis in tissues with macrophages.^567^ Another in vitro study using immuno-engineered human cardiac microtissues and hPSC-derived primitive yolk sac-like macrophages found that macrophages promote cardiomyocyte sarcomeric protein maturation and increase contractile force and relaxation kinetics.^568^ These findings suggest the potential roles of cardiac macrophages in heart development. However, since these results are based solely on in vitro studies, differences with the in vivo developmental environment remain, and further research is needed to confirm these observations.

While these molecular mechanisms regulating heart development have recently garnered researchers’ attention, in-depth studies still need to be completed. It remains to be investigated whether these mechanisms play other significant roles in heart development or whether these novel mechanisms impact classical mechanisms, such as whether autophagy influences heart development via WNT signaling or whether macrophages affect Notch signaling during valve development. Future research is needed to address these questions.

## Diseases related to defects in cardiac development

**Fig. 6:**
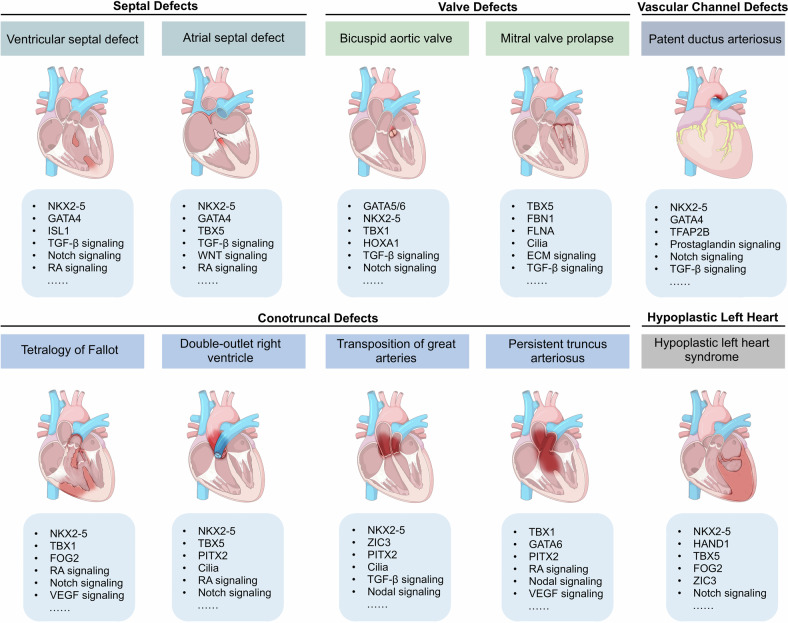
Common congenital heart defects and pathogenic mechanisms. Ventricular septal defect is a defect in the ventricular septum involving the loss of functions of various TFs, GFs, Notch signaling, RA signaling, etc.; atrial septal defect is a defect in the atrial septum, involving the loss of functions of various TFs, GFs, WNT signaling, RA signaling, etc; bicuspid aortic valve is a pathological condition where the aortic valve has only two leaflets instead of the normal three, involving the loss of functions of TGF-β signaling, Notch signaling, various TFs, etc.; mitral valve prolapse is a pathological condition where the mitral valve does not close properly, involving the loss of functions of TGF-β signaling, ECM signaling, FBN1, etc.; patent ductus arteriosus is a pathological condition in which the ductus arteriosus fails to close after birth, allowing a portion of oxygenated blood from the left heart to flow back to the lungs through the aorta, involving the loss of functions of TFAP2B, prostaglandin signaling, Notch signaling, etc.; tetralogy of Fallot includes ventricular septal defects, overriding of the aorta, right ventricular outflow obstruction and right ventricular hypertrophy, involving the loss of functions of various TFs, Notch signaling, VEGF signaling, etc.; double-outlet right ventricle is the defect that both the pulmonary artery and the aorta arise from the right ventricle instead of their normal positions, involving the loss of functions of various TFs, RA signaling, Notch signaling, etc.; transposition of the great arteries is a pathological condition where the pulmonary artery and aorta are switched in position, causing oxygen-rich blood from the lungs is pumped back to the lungs instead of being circulated to the rest of the body, and oxygen-poor blood from the body is pumped back to the body instead of being sent to the lungs to pick up oxygen, involving the loss of functions of cilia, various TFs, Nodal signaling, etc.; persistent truncus arteriosus is a pathological condition characterized by single large vessel arising from the heart that supplies blood to both the systemic and pulmonary circulations, involving the loss of functions of various TFs, RA signaling, Nodal signaling, etc.; hypoplastic left heart syndrome presents with different degrees of stenosis or atresia of the aortic and mitral valve along with hypoplasia of the left ventricle and ascending aorta involving the loss of functions of various TFs, Notch signaling, ECM signaling, etc. ECM extracellular matrix, FBN1 fibrillin 1, FLNA filamin A, FOG2 friend of GATA 2, GATA GATA binding protein, GFs Growth factors, HAND1 heart and neural crest derivatives expressed 1, HOXA1 homeobox A1, ISL1 islet 1, NKX2-5 NK2 homeobox 5, PITX2 paired-like homeodomain transcription factor 2, RA retinoic acid, TBX T-box transcription factor, TFs transcription factors, TFAP2B transcription factor AP-2 beta, TGF-β transforming growth factor beta, VEGF vascular endothelial growth factor, ZIC3 Zic family member 3. This figure was created using Adobe Photoshop

Tracing the precise etiology of CHD remains challenging. Initially, researchers proposed that the occurrence of CHD results from abnormal embryonic development of primitive cardiac segments, manifested anatomically through regional pathology. For instance, abnormal development of the AVC leads to anomalous connections between the atria and ventricles, while anomalies in the primitive outlet manifest as congenital malformations in subarterial ventricular components.^18^ Currently, with ongoing research into the roles of genes and signaling pathways in cardiac development, we can explore the pathogenesis of CHD from a deeper perspective. Each cell involved in cardiac development, starting from stem cell differentiation, requires a regulatory network blueprint to determine its location and function. Individual genes form proteins through transcription and translation processes, thereby fulfilling their predetermined tasks in heart development. Disruption of this process by any factor can lead to various types of heart development defects (Fig. 6). The clinical phenotype of CHD is relatively complex; most patients exhibit only a single type of defect, such as a single ASD or VSD. However, some patients carrying deleterious variants in specific genes, such as *TBX5* or *TBX1* variants, exhibit multiple congenital heart defects combined with developmental disorders in other systems,^569–571^ possibly because the gene mutations causing these complex conditions are upstream in the embryonic developmental regulatory network. For example, TBX5 and TBX1 have been confirmed to be expressed early in mesodermal and SHF development and their mutations lead to severe, multifaceted developmental defects.

In contrast, single types of congenital heart defects may occur when more downstream molecules are affected after specification in other areas, although the etiology of these defects in individuals is currently less understood. Currently, three main mechanisms disrupt normal heart development: inheritance of gene mutations from parents, acquisition of de novo somatic gene mutations during embryogenesis, and disruption by nongenetic factors such as infections, maternal exposure to alcohol or certain drugs, and metabolic disturbances.^572^ However, these mechanisms account for less than half of CHD patients, and further exploration is needed to elucidate the specific pathogenic mechanisms in the remaining CHD patients. Despite some understanding of the signaling pathways and multi-level regulatory mechanisms involved in heart development, our overall knowledge remains quite limited. This contributes to the fact that the etiology in over half of CHD patients is unknown, preventing personalized early prevention and treatment. The complexity of cardiac development is the major challenge. The developmental process is lengthy and involves dynamic changes in numerous molecules and signals at each developmental stage, making it difficult to study. Most studies focus on the role of single molecules or pathways in heart development, with only a small portion examining the cooperative roles of two or more molecules. Therefore, this falls short of explaining the complex developmental networks in both physiological and pathological states.

Advances in sequencing technologies now provide powerful tools for comprehensively understanding heart development and the causes of CHD. For example, metabolomics and epigenomics allow us to observe cardiac development from different perspectives. Single-cell sequencing enables us to study changes in cell phenotypes and signaling pathways at single-cell resolution. Additionally, it allows us to perform pseudo-time and cell communication analysis to observe temporal molecular changes and cell interactions, offering more information than traditional basic researches. As previously mentioned, numerous single-cell sequencing studies have enhanced our understanding of cardiac development at a higher resolution (Table 1). Matthew et al. employed single-nucleus RNA sequencing and imaging mass cytometry to observe cell states in CHD. They found that CHD patients exhibit deficient monocytic immune function and a perivascular microenvironment with spatial distribution characteristics consistent with an immunodeficient state. Additionally, cardiac fibroblasts in HLHS showed signs of activation. These findings provide a theoretical foundation for future personalized treatments for CHD.^573^ However, single-cell sequencing has limitations, such as loss of spatial information and the inability to directly provide functional information. In recent years, spatial transcriptomics has advanced, but its integration with single-cell sequencing is still being refined. While studies using novel sequencing technologies have yielded informative results, we are still far from fully understanding the complex regulatory networks of heart development and the causes of CHD. More research is needed to rigorously validate and explore the findings from sequencing studies. In this section, we will review common congenital heart defects and their cellular and molecular mechanisms.

### Septal defects

Septal defects primarily include ASDs, VSDs, and AVSDs. A ventricular or atrial septal defect is a hole in the septum, the wall that divides the left and right ventricles or atria of the heart. Depending on the location of the defect, ventricular defects can be classified as membranous VSDs, muscular VSDs, inlet VSDs, or infundibular VSDs.^574^ ASD can be classified as a patent foramen ovale, an ostium primum defect, an ostium secundum defect, a sinus venosus defect, a coronary sinus defect, or a common atrium defect.^575^ AVSDs are characterized by a defect in the atrioventricular septum accompanied by malformation of the atrioventricular valves, with or without ventricular septal defects, caused by inadequate development of the endocardial cushion. As mentioned earlier, dysfunctions in BMP4, TGF-β, HAND2, ISL1, TBX5, RA, Notch signals, and other molecules are involved in the formation of septal defects. One of the critical mechanisms through which these molecules contribute to VSD or ASD involves their impact on the normal development of endocardial cushions and the EndMT process. Although multiple molecules and signals have been established as key regulatory factors in cardiac septation, their specific roles in causing different types of septal defects remain unclear. For instance, individuals with *TBX5* mutations may develop ASD, VSD, or AVSD.^576^ The complexity of the regulatory network in cardiac development suggests that a single gene or molecular signal does not solely determine the phenotype of CHD. Development occurs in multiple stages over time, involving changes in cell types and numbers. Disease phenotypes are typically observed in the final stages of development, making it challenging to establish a direct link between abnormal gene mutations or molecular signals and the final disease phenotype. This complexity also implies that abnormal molecular signals may undergo further regulation or cause additional abnormalities in intermediate stages, thereby leading to different downstream disease phenotypes. Further in vitro and in vivo studies are needed to confirm these relationships and establish clearer connections between the discovered molecular abnormalities and specific congenital heart disease phenotypes.

### Conotruncal defects

Conotruncal defects arise due to inadequate or misaligned development of the ventricular septum, OFT, and/or great arteries, resulting in conditions such as ToF, PTA, DORV, and TGA. PTA, which is associated with DiGeorge syndrome (chromosome 22q11.2 deletion syndrome) and *TBX1* deficiency, is a rare congenital heart defect characterized by abnormal embryonic cardiac development, leading to the absence of normal separation between the aorta and pulmonary artery during fetal development. Instead of two separate arteries, there is a single large vessel arising from the heart that supplies blood to both the systemic and pulmonary circulations.^577,578^ DORV occurs when both the pulmonary artery and the aorta, the two main arteries that carry blood away from the heart, originate from the RV rather than from their usual positions. In Obler et al.‘s study, both chromosomal and nonchromosomal abnormalities were associated with this phenotype, with mutations in the Cryptic Family 1 (*CFC1*) and chicken skeletal muscle X-linked (*CSX*) genes identified in very few cases.^579^ ToF, the most common cyanotic CHD, consists of four abnormalities: a ventricular septal defect, overriding aorta, right ventricular outflow obstruction, and right ventricular hypertrophy. It typically occurs sporadically but is also observed in conjunction with other syndromes. The pathogenesis of conotruncal defects primarily involves molecular signaling dysfunction in two crucial pathways related to the development of SHF and CNCCs. Loss-of-function mutations affecting molecules and signaling, such as *FGF8*, *FGF10*, *NKX2-5*, *ISL1*, Shh, and Notch signals, are implicated in conotruncal defects. Specifically, disruptions in FGF signaling, ISL1, and Notch signaling during the migration and functional integration of CNCCs are considered major factors contributing to these diseases.^580,581^

### Valve defects

Common congenital valve developmental abnormalities include BAV, mitral valve prolapse (MVP), mitral stenosis, tricuspid atresia, and PVS. Although these developmental malformations are collectively referred to as valve defects, the molecular mechanisms underlying their disease phenotypes may be entirely distinct. As discussed in the previous section, valve formation begins with the expansion of cardiac jelly and the differentiation of endocardium-derived mesenchymal cells, with varying cellular origins and regulatory mechanisms involved in different valve developments. For instance, the mitral and tricuspid valves derive from atrioventricular valve development, while OFT cushions form the aortic and pulmonary valves, requiring migration from the CNCCs. Therefore, disruptions in related molecular regulatory networks, such as Notch signaling and TGFβ signaling, may lead to valve defects, but a precise understanding of the pathogenesis of various valve defects is still lacking. BAV is a congenital defect characterized by the aortic valve having only two leaflets instead of the normal three leaflets. Animal models of BAV are primarily constructed using genes such as *Notch1* and *Gata5/6*, and multiple genes, including *TGFBR2*, *TGFBR1*, *NOTCH1*, *ACTA2*, and *KCNJ2*, have been associated with BAV.^582^ MVP is a pathological condition in which the mitral valve fails to close properly due to myxomatous degeneration, primarily caused by dysregulated TGFβ signaling, leading to the differentiation of valve interstitial cells into myofibroblasts and subsequent production and deposition of extracellular matrix, resulting in structural changes in the mitral valve.^583,584^ Ebstein anomaly is a rare congenital heart defect characterized by abnormalities in the tricuspid valve, which is located between the right atrium and the right ventricle of the heart. In individuals with Ebstein anomaly, the tricuspid valve is positioned abnormally low in the right ventricle, resulting in decreased blood flow to the lungs and reduced oxygen levels in the body.^585^ The pathogenesis of Ebstein anomaly remains unclear, but previous studies have suggested that mutations in the *MYH7* gene are associated with this condition.^586,587^

### Hypoplastic left heart syndrome

HLHS presents with varying degrees of aortic and mitral valve stenosis or atresia, along with hypoplasia of the left ventricle and ascending aorta. Studies using hPSCs to investigate HLHS have indicated intrinsic myocardial cell defects as potential causes, characterized by decreased cardiac differentiation efficiency, sarcomere disarray, abnormal mitochondrial structure, and impaired Notch signaling.^588–590^ Single-cell sequencing of induced pluripotent stem cell-derived endothelial cells (iECs) revealed abnormal ECM deposition and EndoMT in endothelial cells, leading to reduced proliferation and maturation of cardiomyocytes.^591^ Various gene mutations, such as those in RNA binding fox-1 homolog (*RBFOX*)-*2*, myelin regulatory factor (*MYRF*), and *HAND1*, have also been implicated in the occurrence of HLHS.^592^ However, while these in vitro studies provide insights into disease onset, a precise explanation for HLHS pathogenesis, particularly how abnormal differentiation of myocardial and endothelial cells leads to valve developmental defects, requires further validation through in vivo studies.

### Patent ductus arteriosus

PDA is a condition in which the ductus arteriosus fails to close after birth, allowing some oxygenated blood from the left heart to flow back to the lungs through the aorta. The ductus arteriosus is crucial for fetal survival in the uterus. After birth, as the partial pressure of oxygen increases and the levels of prostaglandins and other vasodilators decrease, the ductus arteriosus constricts and closes, eventually transforming into the ligamentum arteriosum.^593^ This process involves two main stages: initial “functional” closure through smooth muscle contraction, followed by “anatomical” closure characterized by neointimal thickening and a reduction in smooth muscle cells in the inner muscle layer.^594^ Thus, normal smooth muscle contraction and the accumulation of various ECM components in the subendothelial area are critical for the closure of the ductus arteriosus. Deficiencies in multiple ECM molecules can lead to the PDA phenotype.^595–597^ Clinically, the use of nonsteroidal anti-inflammatory drugs (NSAIDs) to inhibit cyclooxygenase and induce ductus arteriosus closure highlights the critical role of prostaglandin signaling in this process.^598^ Gene knockout studies have shown that various molecules involved in prostaglandin signaling are essential for ductus arteriosus closure, but their absence often results in a PDA phenotype and neonatal lethality.^599–602^ Notably, recent research suggests that the occurrence of PDA may be due not only to postnatal dysfunction of smooth muscle cells, ECM, and endothelial cells but also to abnormal vascular smooth muscle differentiation during heart development. For example, a defect in the smooth muscle cell-related gene *Myh11* can delay ductus arteriosus closure,^603^ and knocking out *Myocd* in neural crest-derived SMCs results in a PDA phenotype with a loss of SMC markers,^604^ underscoring the importance of normal vascular smooth muscle development for ductus arteriosus closure. Furthermore, in mice, defects in smooth muscle Notch signaling can lead to PDA. Conditional knockout of *Jag1* in mouse smooth muscle cells causes defects in the PDA and OFT, with reduced expression of mature smooth muscle cell markers in the OFT; moreover, indomethacin can partially rescue the PDA in these mice.^605^ Subsequent studies on smooth muscle *Rbpj* deficiency revealed phenotypes similar to those of *Jag1* deficiency, with only a few gene-deficient mice being rescued by indomethacin.^606^ Another study indicated that *Notch2* and *Notch3* play crucial roles in promoting vascular smooth muscle cell development and functional closure of the ductus arteriosus.^607^

### Developmental diseases presenting with cardiac congenital defects

Numerous congenital conditions affect various parts of the body and are often accompanied by a high incidence of cardiac congenital defects, leading to a spectrum of distinctive features and potential health complications. Most of these diseases are caused by specific genetic defects, with some of the key signals involved in heart development also playing a role. We have summarized these conditions in Table 5.

**Table 5:** Developmental diseases presenting with cardiac congenital defects

Disease	Molecular mechanisms	Genetic Defects	Cardiac phenotypes	Occurrence of CHD	Ref (PMID)
Down syndrome		The presence of three copies of the Homo sapiens chromosome 21	AVSD, VSD, ASD, PDA and ToF	40–50%	36769235
CHARGE syndrome		Pathogenic variants in CHD7	PDA, VSD, ASD, ToF and aortic abnormalities	76%	37675914
Heterotaxy	Abnormalities in cilia; Dysregulation of FGFs, Nodal, Notch, PITX2 and BAF60C	Pathogenic variants in ZIC3, CRYPTIC, NODAL, CFC1, ACVR2B、LEFTY2, CITED2, GDF1, DNAH10, KIF7, FLNA and KMT2D	AVSD (most common), TGA, DORV, ToF, single ventricle, PA, anomalous pulmonary venous drainage, PS, left ventricular outflow tract obstruction, double inlet left ventricle, aortic coarctation, atrial isomerism, bilateral/ hypoplastic/absent sinus node(s), single coronary artery, interrupted inferior vena cava and bilateral superior vena cava	90%	27541719, 32738303, 22864291
DiGeorge syndrome	Abnormalities in RA signaling	Deletion on the long arm (q) at the 11.2 locus in chromosome 22 (22q11.2)	ToF (most common), IAA, TA, VSD, PA, MAPCA, RAA, ASD, PDA, DORV, APV, PS, BAV	60–80%	36897497, 37090828, 32049433
Holt-Oram syndrome		Pathogenic variants in TBX5	ASD (most common), VSD, AVSD, ToF, cardiac arrhythmias, CoA, HLHS, persistent superior vena cava and MVP	70–95%	30552424, 35514310
Noonan syndrome	Dysregulation of RAS-MAPK signaling pathway	Pathogenic variants in PTPN11, SOS1, KRAS, NRAS, RAF1, BRAF, SHOC2, CBL	PVS (most common), ASD, VSD, AVCD, AVSD, AS, PDA, ToF, aortic coarctation, peripheral pulmonary stenosis, mitral valve abnormalities and coronary artery abnormalities	80-90%	32022400, 27541719
Turner syndrome		Monosomy X	BAV (most common), CoA, partial anomalous pulmonary venous connection, left superior vena cava, elongated transverse, arch and dilatation of the brachiocephalic arteries, HLHS, mitral valve anomalies, interrupted inferior vena cava with azygous continuation, cardiac dextroposition, VSD, AVSD, pulmonary valve abnormalities and coronary artery anomalies	50%	33307001
Jacobsen Syndrome		Deletion of chromosome 11q	VSD (most common), left ventricular outflow tract defects, mitral valve abnormalities, HLHS, DORV, TGA, AVSD, ASD, dextrocardia, aberrant right subclavian artery, PDA, persistent left superior vena cava, tricuspid atresia, IAA, TA, and PVS	50%	36661903
1p36 syndrome		Deletion of chromosome 1p36	ASD (most common), VSD, valvular abnormalities, PDA, ToF, CoA, infundibular stenosis of the right ventricle, and Ebstein’s anomaly	71%	18245432
Alagille syndrome		Pathogenic variants in JAG1 or Notch2	Peripheral pulmonary artery stenosis and/or hypoplasia (most common), ToF, valvular/supravalvular aortic stenosis, PA	90%	35868679
Ellis-van Creveld syndrome		Pathogenic variants in DYNC2H1, DYNC2LI1, EVC, EVC2, GLI, SMO, WDR35, PRKACA or PRKACB	ASD (most common), VSD, single atrium, and left superior vena cava, hypoplastic left ventricle, pulmonary valve stenosis/atresia, and CoA	66%	37903214
Costello syndrome	Dysregulation of RAS-MAPK signaling pathway	Pathogenic variants in HRAS	PVS (most common), ASD, VSD	40%-50%	21344638
Cardiofaciocutaneous syndrome	Dysregulation of RAS-MAPK signaling pathway	Pathogenic variants in BRAF, MAP2K1, MAP2K2, KRAS or YWHAZ	PVS (most common), ASD, VSD, mitral valve dysplasia, arrhythmias, tricuspid valve dysplasia, and BAV	75%	38136934
Kabuki syndrome		Pathogenic variants in KMT2D or KDM6A	CoA (most common), septal defects, BAV, mitral valve anomalies, conotruncal heart defects, and HLHS	70%	21882399
Marfan syndrome		Pathogenic variants in FBN1	Aortic root dilatation, plurivalvular regurgitation, BAV and MVP	50-75%	38667733
Cantu syndrome		Pathogenic variants in ABCC9 or KCNJ8	Cardiac enlargement (most common), PDA, BAV, mitral valve regurgitation, aortic valve stenosis, dilated aortic root, and pericardial effusion	75%	25275207 30571578
Char syndrome		Pathogenic variants in TFAP2B	PDA (most common), VSD	74%	20301285
Carpenter Syndrome		Pathogenic variants in RAB23	VSD, ASD, PDA, PS, ToF	18%-50%	25162549
Mowat-Wilson syndrome		Pathogenic variants in ZEB2	Septal defects and patent ductus arteriosus (most common), PS, CoA, BAV, Aortic valve stenosis, ToF, pulmonary artery sling	58%	20301585
Smith-Lemli-Opitz syndrome		Pathogenic variants in DHCR7	AVCD, anomalous pulmonary venous return, PS	50%	20301322

*ACVR2B* activin A receptor type 2B, *APV* absent pulmonary valve, *ASD* atrial septal defect, *AVCD* atrioventricular canal defects, *AVSD* atrioventricular septal defect, *BAV* bicuspid aortic valve, *BRAF* B-Raf proto-oncogene serine/threonine kinase, *CBL* Casitas B-lineage lymphoma, *CHD* congenital heart disease, *CHD7* chromodomain helicase DNA binding protein 7, *CITED2* cAMP response element-binding protein-binding protein(Cbp)/p300-interacting transactivator with glutamic acid (Glu)/aspartic acid (Asp)-rich carboxy-terminal domain 2, *CoA* coarctation of the aorta, *CFC1* cripto fibroblast growth factor receptor-like 1 (FRL-1) cryptic family 1, *CRYPTIC* cryptic family protein, *DNAH10* dynein axonemal heavy chain 10, *DORV* double outlet right ventricle, *DYNC2H1* dynein cytoplasmic 2 heavy chain 1, *DYNC2LI1* dynein cytoplasmic 2 light intermediate chain 1, *EVC* Ellis-van Creveld syndrome protein, *FBN1* fibrillin 1, *FGFs* fibroblast growth factors, *FLNA* filamin A, *GDF1* growth differentiation factor 1, *HLHS* hypoplastic left heart syndrome, *IAA* interrupted aortic arch, *JAG1* jagged 1, *KCNJ8* inward rectifier potassium channel 8, *KIF7* kinesin family member 7, *KDM6A* lysine-specific demethylase 6A, *KMT2D* lysine methyltransferase 2D, *KRAS* Kirsten rat sarcoma viral oncogene homolog, *LEFTY2* left-right determination factor 2, *MAP2K1* mitogen-activated protein kinase kinase 1, *MAP2K2* mitogen-activated protein kinase kinase 2, *MAPK* mitogen-activated protein kinase, *MAPCA* major aortopulmonary collateral arteries, *MVP* mitral valve prolapse, *NRAS* neuroblastoma RAS viral oncogene homolog, *PA* pulmonary atresia, PDA patent ductus arteriosus, *PITX2* paired-like homeodomain transcription factor 2, *PRKACA* protein kinase A catalytic subunit alpha, *PRKACB* protein kinase A catalytic subunit beta, *PS* pulmonary stenosis, *PVS* pulmonary valve stenosis, *RA* retinoic acid, *RAA* right aortic arch, *RAS* rat sarcoma viral oncogene homolog, *RAB23* RAB23 member RAS oncogene family, *RV* right ventricle, *SHOC2* SHOC2 leucine-rich repeat scaffold protein, *SOS1* SOS Rat sarcoma (Ras)/Ras-related C3 botulinum toxin substrate (Rac) guanine nucleotide exchange factor 1, *TA* truncus arteriosus, *TBX* T-box transcription factor, *TGA* transposition of the great arteries, *ToF* Tetralogy of Fallot, *TFAP2B* transcription factor activating protein (AP)-2 beta, *VSD* ventricular septal defect, *WDR35* tryptophan-aspartate (WD) repeat domain 35, *YWHAZ* tyrosine 3-monooxygenase/tryptophan 5-monooxygenase activation protein zeta, *ZEB2* zinc finger E-box binding homeobox 2, *ZIC3* zinc finger protein of the cerebellum (Zic) family member 3

## Treatment for congenital heart defects

For a long time, many children born with CHD had limited treatment options, often receiving only palliative care during their early years. In the early to mid-20th century, physicians began attempting surgical treatments for children with CHD. Recently, more surgical techniques have been developed and applied to various types of CHD. Procedures such as Fontan surgery, atrial redirection, and arterial switch operations for treating complex CHD have since been established. Currently, surgery has become the primary treatment for CHD.^608^ While these new surgical techniques, along with interventional catheterizations, have reduced risks for less complex CHD cases, CHD remains a global health challenge due to the complexity of surgeries, high treatment costs, poor quality of life for patients, and challenges in managing complications.^609^ Compared to the development of surgical treatments for CHD, progress in pharmacological treatments has been slower. Most medications are used to improve and stabilize the preoperative condition of patients, manage postoperative complications, or provide palliative care. The use of NSAIDs for treating PDA has provided an alternative treatment method because of a deeper understanding of PDA pathogenesis. The scarcity of medical treatment options for CHD reflects our insufficient understanding of the mechanisms underlying congenital heart defects. In this section, we summarize the current strategies for treating CHD and discuss advancements in therapeutic approaches for this condition.

### The current treatment strategies for CHD

Currently, CHD is considered a lifelong chronic condition. CHD patients often require one or more surgeries or interventional treatments during infancy and early childhood. After surgery, patients may experience complications such as heart failure, stroke, arrhythmias, reduced exercise tolerance, and increased risk of sudden death throughout their lives. Thus, early surgical treatment and lifelong management are equally important in treating CHD.^610^ Current treatment strategies for CHD encompass a range of approaches, including surgical interventions such as surgical closure for atrial/ventricular septal defects, PDA occlusion, the Mustard or Senning atrial switch procedure for transposition of the great arteries, and heart transplantation for end-stage heart failure. However, for some of the most lethal CHDs, such as HLHS, although surgical interventions have significantly improved survival rates, complex and severe complications may still develop, and most patients inevitably progress to heart failure.^611^ The staged palliative surgical approach for HLHS has been developed to simulate a physiological circulation and includes three stages: the first stage palliation, the second stage palliation, and the Fontan procedure.^612^ The first-stage treatments include the Norwood procedure with Blalock-Taussig shunt (BTS), Norwood with Sano modification, and the hybrid procedure. Long-term survival data for these methods remain limited. In high-volume centers, the postoperative mortality rate for Norwood surgery is approximately 22%, with common complications including respiratory insufficiency (21.6%), arrhythmias (19%), and cardiac arrest (12%).^613^ The other two surgical approaches do not show significant advantages in survival probability compared to the Norwood procedure.^611^ The second stage, typically performed 2 to 6 months after the Norwood surgery, is known as the bidirectional Glenn procedure. Several years later, the third stage, the Fontan procedure, is performed to direct blood flow from the superior and inferior vena cava directly to the pulmonary arteries, bypassing the right ventricle. However, up to 50% of patients with Fontan circulation may experience significant adverse events before adulthood, including late failure, reoperation, percutaneous interventions, pacemaker implantation, thromboembolic events, or supraventricular tachycardia.^614^

Although the Fontan procedure can temporarily improve cardiac function, over time, Fontan circulation can lead to increased central venous pressure, organ congestion, cardiac remodeling, and decreased cardiac output, resulting in cardiovascular and non-cardiovascular complications. Single ventricle patients after Fontan palliation, particularly those with heart failure, can exhibit significant metabolic changes, including altered substrate consumption, induced ketolysis, and abnormal glucose-lipid metabolism patterns, which may affect the function of other organs such as the liver, kidneys, nervous system, and lymphatics.^615^ Post-Fontan myocardial fibrosis and electrophysiological remodeling greatly increase the risk of heart failure.^616^ SGLT2 inhibitors can reduce edema and lower plasma brain natriuretic peptide levels in post-Fontan patients.^617,618^ Case series have reported that Angiotensin Receptor Neprilysin Inhibitors (ARNIs) can improve right ventricular systolic function, reduce hospitalization rates, and significantly improve protein-losing enteropathy symptoms, though NYHA functional class symptoms do not show significant changes.^619^ Post-Fontan surgical trauma, fibrosis, and cardiac remodeling may contribute to the development of electrophysiological remodeling and arrhythmias. Common arrhythmias include sick sinus syndrome, supraventricular tachycardias, atrial tachycardia, and atrial fibrillation.^620^ In canine models, post-Fontan surgery has been shown to cause electrophysiological changes and ionic remodeling, including shortened refractory periods, altered ion channel expression, and induced atrial tachycardia.^621^ For atrial arrhythmias such as intra-atrial re-entrant tachycardia (IART), nonautomatic focal atrial tachycardia (NAFAT), and atrial fibrillation, catheter ablation can reduce arrhythmia burden, but it is also associated with complications such as atrioventricular block and residual inducible arrhythmias, with a lower success rate compared to non-Fontan patients.^622–624^ Pacemaker implantation benefits hemodynamics and symptom relief, and recent guidelines recommend pacemaker implantation for treating Fontan circulation sinus node dysfunction.^624^ However, permanent ventricular pacing increases the risk of transplantation and late death in post-Fontan patients, potentially due to the wide QRS complex produced. Optimizing pacemaker therapy, including shortening QRS duration and maintaining atrioventricular synchrony through atrial pacing or adjusting the atrioventricular interval, may mitigate the adverse hemodynamic effects of pacemaker treatment.^625–627^ Thus, current treatment options for adverse outcomes due to Fontan surgery-related remodeling remain limited, and clinical evidence is still insufficient, posing challenges for managing post-Fontan patients. The risk of severe complications remains very high for post-Fontan patients, including ventricular systolic dysfunction, arrhythmias, Fontan-associated liver disease, renal dysfunction, protein-losing enteropathy, and plastic bronchitis.^628^ Therefore, while surgical interventions have significantly improved survival probabilities for patients with lethal CHDs, lifelong management remains a challenge.

Additionally, for treatment of CHD, interventional catheterizations can be performed either as standalone procedures or as part of hybrid procedures, avoiding the need for sternotomy/thoracotomy. Common interventional catheterizations include closure of shunt lesions or unusual collaterals, balloon dilation or stenting of narrowed great vessels, or transcatheter pulmonary valve implantation. Pharmacological treatments are also utilized to manage circulation issues. These include diuretics, beta-blockers, and angiotensin-converting enzyme inhibitors for heart failure; prostaglandin drugs and endothelin receptor antagonists for pulmonary hypertension;^629,630^ and anticoagulants, antithrombotics, antiarrhythmics, and antihypertensive medications for symptomatic treatment. Dedicated centers should provide specialized lifelong care for CHD patients with expertise in pediatric cardiology and cardiac surgery. These strategies aim to address the diverse spectrum of CHD severity and complexity, with advancements in surgical techniques and ongoing research contributing to improved outcomes and quality of life for patients. The interventional and randomized clinical trials for the treatment of CHD are provided in Table 6.

**Table 6:** Current randomized trials of treatment of congenital heart diseases

Treatment class	Conditions	Interventions	Start time	Status	Location	Identifier
Procedural treatment	Tetralogy of Fallot/Pulmonary Valve Insufficiency/Ventricular Dysfunction, right	PROCEDURE: PVR plus RV remodeling/PROCEDURE: Standard PVR	2004-04-01	NA/Completed	United States	NCT00112320
	Heart Defects, Congenital	PROCEDURE: Blalock-Taussig pulmonary artery shunt/PROCEDURE: Right ventricular to pulmonary artery shunt	2005-05-01	Phase III/Completed	United States/Canada	NCT00115934
	Congenital Heart Disease	PROCEDURE: Remote ischemic preconditioning	2008-01-01	Phase I&II/Completed	Brazil	NCT00868101
	Congenital Heart Disease	PROCEDURE: Norwood management strategy/PROCEDURE: Hybrid Strategy	2010-03-01	Phase III/Unknown	Canada	NCT01134302
	Heart Defects, Congenital	PROCEDURE: Remote Ischemic Preconditioning/OTHER: Control	2011-09-01	NA/Completed	United States	NCT01835392
	Hypogammaglobulinemia/Congenital Heart Disease	Drug: IVIG/Other: Placebo	2014-05-01	NA/Completed	United States	NCT02043379
	Congenital Heart Disease	Procedure: Surgery with CPB/Other: Fresh Frozen Plasma/Other: Plasmalyte	2015-10-01	NA/Completed	Belgium	NCT02567786
	Persistent Ductus Arteriosus	PROCEDURE: Surgical treatment/DRUG: Control group	2015-10-01	Phase II/Unknown	Mexico	NCT02602054
	Perimembranous Ventricular Septal Defect	PROCEDURE: transthoracic device closure/PROCEDURE: surgical repair	2015-10-01	Phase II&III/Unknown	China	NCT02644330
	Cardiac Surgery/Congenital Heart Defects/Cardiopulmonary Bypass	Drug: Dexamethasone/Drug: Placebo	2015-12-01	Phase III/Completed	Brazil/China/ Russian Federation	NCT02615262
	Heart Defects, Congenital	PROCEDURE: Right lateral position (2 hours)/PROCEDURE: Left lateral position (30 minutes)/PROCEDURE: Supine position (30 minutes)/PROCEDURE: Left lateral position (2 hours)/PROCEDURE: Supine position (2 hours)	2016-01-30	NA/Unknown	Egypt	NCT02622152
	Pulmonary Atresia with Ventricular Septal Defect/Tetralogy of Fallot with Pulmonary Atresia	PROCEDURE: Experimental: RVOT reconstruction by femoral allogenic vein valve conduit/PROCEDURE: Systemic-to-pulmonary artery shunts	2016-05-05	NA/Completed	Russian Federation	NCT02861963
	Coarctation of Aorta	PROCEDURE: Surgical repair of coarctation of aorta	2016-07-01	NA/Unknown	Russian Federation	NCT02835703
	Congenital Heart Disease/Oral Aversion	PROCEDURE: Endotracheal intubation	2018-07-01		United States	NCT05378685
	Congenital Heart Disease	PROCEDURE: Paravertebral Nerve Block/DRUG: Standard of Care Anesthesia	2018-07-18	Phase IV/Recruiting	United States	NCT03408340
	Congenital Heart Disease/Mechanical Ventilation Complication/Cardiopulmonary Bypass	PROCEDURE: Ventilation during cardiopulmonary bypass	2018-08-20	NA/Unkown	Italy	NCT03629574
	Tetralogy of Fallot/Pulmonary Regurgitation	PROCEDURE: Pulmonary valve replacement	2019-04-01	NA/Recruiting	Denmark	NCT04084132
	Grown-up Congenital Heart Disease	PROCEDURE: Oxygen application/PROCEDURE: Sham room air	2019-09-12	NA/Completed	Switzerland	NCT04076501
	Heart Defects, Congenital	PROCEDURE: Ventricular septal defect closure surgery/PROCEDURE: Ventricular septal defect closure catheter	2020-06-12	NA/Completed	Egypt	NCT05306483
	Congenital Heart Disease	PROCEDURE: SC TAP/PROCEDURE: Control	2021-01-15	NA/Completed	Korea	NCT04570878
	Cardiac Septal Defects with Coarctation of the Aorta/Mitral Regurgitation	PROCEDURE: Ultrasound guided Pecto-intercostal- fascial plane block/PROCEDURE: Ultrasound guided erector spinae plane block/DRUG: Bupivacain	2022-05-25	Phase II/Completed	Egypt	NCT05475561
	Aortic Stenosis with Bicuspid Valve	PROCEDURE: TAVR for BAV Using Down Sizing with the Evolut Pro platform/PROCEDURE: TAVR for BAV Using Traditional Sizing strategy with the Evolut Pro platform	2022-06-27	NA/Recruiting	China	NCT05511792
	Congenital Heart Disease	PROCEDURE: Serratus anterior plane block/PROCEDURE: erector spinae plane blocks/DRUG: Bupivacaine 0.25% Injectable Solution	2022-07-24	NA/Completed	Egypt	NCT05526469
	Atrial Septal Defect/Ventricular Septal Defect/Pain, Procedural	PROCEDURE: Regional Anesthesia/PROCEDURE: Wound infiltration/DRUG: Ropivacaine 0.2% Injectable Solution	2023-03-29	Phase IV/Recruiting	United States	NCT05688670
	Transposition of Great Vessels/Tetralogy of Fallot/Truncus Arteriosus/Pulmonary Artery Stenosis Supravalvular Congenital/Stent Stenosis/Right Ventricular Dysfunction/Congenital Heart Disease	PROCEDURE: Percutaneous intervention (stent) for PA stenosis	2023-04-18	NA/Recruiting	Netherlands	NCT05809310
	Complex Congenital Heart Disease/Enhanced Recovery After Surgery	PROCEDURE: ERAS	2023-07-01	NA/Not yet recruiting	China	NCT05914103
	Congenital Heart Disease	PROCEDURE: Umbilical Cord Clamping at ~30 seconds/PROCEDURE: Umbilical Cord Clamping at ~120 seconds/PROCEDURE: Umbilical Cord Milking	2023-12-19	NA/Recruiting	United States/Canada	NCT06153459
	Congenital Heart Disease	PROCEDURE: Bilateral two-level serratus anterior block	2024-02-10	NA/Not yet recruiting		NCT06221150
	Congenital Heart Disease	PROCEDURE: High Flow Nasal Canula following extubation/PROCEDURE: Non-Invasive Positive Pressure Ventilation following extubation	2024-05-01	NA/Not yet recruiting	United States	NCT05869825
Pharmacological treatment	Turner Syndrome	DRUG: estrogen/DRUG: androgen/OTHER: placebo	1992-11-01	Phase III/Completed	United States	NCT00029159
	Patent Ductus Arteriosus	DRUG: Continuous indomethacin/DRUG: ibuprofen	2002-02-01	Phase III/Completed	Israel	NCT00485160
	Cardiac output, low	Drug: Hydrocortisone/ Other: Placebo	2003-02-01	Phase II/Completed	United States	NCT00590018
	Heart Defects, Congenital/Heart Failure, Congestive	Drug: Enalapril/Drug: Placebo	2003-08-01	Phase III/Completed	United States	NCT00113087
	Transposition of Great Vessels/Congenital Heart Disease	DRUG: N-acetylcysteine/DRUG: Placebo	2005-02-01	Phase II/Completed	United States	NCT00374088
	Turner Syndrome	DRUG: estradiol	2005-06-01	Phase IV/Completed	Denmark	NCT00134745
	Turner Syndrome	DRUG: ZOMACTON	2005-10-01	Phase III/Completed	Czech Republic/France/Netherlands	NCT00250250
	Blood Loss/Congenital Heart Disease	DRUG: Tranexamic Acid	2006-01-01	NA/Completed		NCT00994994
	Heart Defects, Congenital	Drug: Clopidogrel (SR25990)/ Drug: placebo	2006-11-01	Phase III/Completed	United States	NCT00396877
	Marfan Syndrome	DRUG: Losartan Potassium/DRUG: Atenolol	2007-01-01	Phase III/Completed	United States/Belgium/Canada	NCT00429364
	Congenital Heart Disease/Disorder of Fetus or Newborn	Drug: IVMP/Drug: two doses IVMP	2007-03-01	NA/Completed	United States	NCT00934843
	Turner Syndrome	DRUG: Growth Hormone/DRUG: Placebo/OTHER: Healthy controls	2007-08-01	NA /Completed	Denmark	NCT00420654
	Cerebral Oxygenation	DRUG: nitroprusside/DRUG: nitroglycerine/DRUG: sevoflurane	2007-10-01	Phase II /Completed	Belgium	NCT00535808
	Marfan Syndrome	DRUG: Atenolol/DRUG: Losartan	2007-10-01	Phase III /Completed	United States	NCT00723801
	Hypoplastic Left Heart Syndrome/Tricuspid Atresia	DRUG: Sildenafil/DRUG: Placebo	2007-12-01	Phase II /Completed		NCT00507819
	Congenital Heart Disease	DRUG: Nitric Oxide	2008-01-01	Phase I&II /Completed	United States	NCT00585013
	Eisenmenger Syndrome	DRUG: Tadalafil, placebo	2008-02-01	Phase I /Completed	India	NCT01200732
	Eisenmenger Syndrome	DRUG: Bosentan and Sildenafil	2006-03-01	Phase III /Completed	Denmark	NCT00303004
	Patent Ductus Arteriosus	DRUG: Indomethacin/DRUG: Pentoxifylline	2008-03-01	Phase II/Unknown	Israel	NCT00616382
	Ductus Arteriosus, Patent	DRUG: Ibuprofen	2008-06-01	Phase II&III/Completed	Italy	NCT01243996
	Marfan Syndrome	DRUG: Losartan and nebivolol/DRUG: Losartan/DRUG: Nebivolol	2008-07-01	Phase III/Unknown	Italy	NCT00683124
	Congenital Cardiovascular Defects/Low Cardiac Output Syndrome	Drug: Levosimendan/Drug: Milrinone	2008-07-01	NA/Completed	Belgium	NCT00695929
	Transposition of Great Vessels/Atrial Switch Procedure	Drug: Eplerenone	2008-07-01	Phase IV/Completed	Spain	NCT00703352
	Marfan Syndrome	DRUG: Losartan/DRUG: Atenolol	2008-10-01	Phase III/Unknown	Spain	NCT01145612
	Tetralogy of Fallot /Ventricular Septal Defects /Atrioventricular Septal Defects	DRUG: Fentanyl (High Dose)/DRUG: Fentanyl (Low Dose)/DRUG: Fentanyl (Low Dose) + Dexmedetomidine	2008-11-01	Phase II/Completed	United States	NCT00848393
	Patent Ductus Arteriosus	DRUG: oral ibuprofen	2009-01-01	NA/Completed	Turkey	NCT01261117
	Patent Ductus Arteriosus	Drug: Ibuprofen EchoG/Drug: Standard ibuprofen treatment	2009-05-01	Phase III/Completed	Spain	NCT01593163
	Congenital Heart Disease	DRUG: moderate potassium group/DRUG: High potassium group	2009-10-01	NA/Completed	China	NCT01479049
	Low Cardiac Output Syndrome	DRUG: Milrinone/DRUG: Levosimendan	2009-11-01	Phase I&II/Completed	Spain	NCT01576094
	Marfan Syndrome	DRUG: Aliskiren/DRUG: Atenolol	2010-06-01	Phase III/Completed	Korea	NCT01715207
	Atrial Septal Defect/Ventricular Septal Defect/Atrioventricular Septal Defect	DRUG: Intravenous L-Citrulline/DRUG: Placebo of Intravenous L-Citrulline	2010-09-01	Phase I&II/Completed	United States	NCT01120964
	Heart Septal Defects, Atrial	DRUG: Bosentan/DRUG: Placebo	2010-10-01	Phase III/Completed	Belgium	NCT01218607
	Pediatric Ventricular Septal Defects	DRUG: sufentanil anesthesia/PROCEDURE: Hybrid closure/PROCEDURE: Control	2011-01-01	NA/Completed		NCT02794584
	Hypoplastic Left Heart Syndrome/Tricuspid Atresia/Other Specified Congenital Anomalies of Heart	DRUG: Bosentan/DRUG: Placebo	2011-02-01	Phase II/Completed	Denmark/ Sweden	NCT01292551
	Children With a Univentricular Heart Undergoing Surgery for Creation of a Fontan Circulation	DRUG: Administration of an ACE-inhibitor or not	2011-09-01	Phase II/Completed	Belgium	NCT00263406
	Congenital Heart Disease	DRUG: Bosentan	2011-09-01	NA/Unknown	Netherlands	NCT01184404
	Congenital Heart Diseases	DRUG: Sevoflurane/OTHER: Control	2011-09-01	NA/Unknown	China	NCT01450956
	Patent Ductus Arteriosus	DRUG: Oral paracetamol/DRUG: Oral ibuprofen	2012-02-01	Phase IV/Completed	Turkey	NCT01536158
	Patent Ductus Arteriosus	DRUG: Ibuprofen/DRUG: Placebo	2012-03-01	Phase III/Completed	France	NCT01630278
	Heart Disease Congenital Complex	Drug: Hydrocortisone/Drug: Normal Saline	2012-04-01	NA/Completed	United States	NCT01595386
	Patent Ductus Arteriosus	DRUG: Paracetamol/DRUG: NSAID/DRUG: D5W	2012-04-01	Phase II/Unknown	Israel	NCT01291654
	Congenital Heart Disease/Disorder of Fetus or Newborn	DRUG: Methylprednisolone/DRUG: Placebo	2012-06-01	NA/Completed	United States	NCT01579513
	Pulmonary Hypertension	DRUG: iloprost nebulizer solution/DRUG: distilled water	2012-06-01	Phase III/Completed	China	NCT01598441
	Single Ventricle Fontan Palliation	DRUG: Ambrisentan	2012-08-01	Phase IV/Completed	United States	NCT01971580
	Congenital Heart Disease/Heart Valve Disease	DRUG: simvastatin	2013-01-01	NA/Recruiting	China	NCT01653223
	Congenital Heart Disease	DRUG: Milrinone	2013-04-01	Phase II/Completed	Canada	NCT01841177
	Pulmonary Arterial Hypertension	DRUG: Macitentan 10 mg/DRUG: Placebo	2013-05-21	Phase III/Completed	United States/ Austria /Bulgaria/Chile/China/France/Germany/ Greece/ Mexico/Poland/ Romania/ Russian Federation/ Serbia/ Spain/ United Kingdom/Turkey/ Vietnam	NCT01743001
	Alagille Syndrome	DRUG: LUM001/DRUG: Placebo	2013-08-01	Phase II/Completed	United Kingdom	NCT01903460
	Persistent Ductus Arteriosus/Complication of Prematurity/Pain or Discomfort in Intensive Care of Preterm Infants	DRUG: paracetamol/DRUG: 0.45% saline solution	2013-08-01	Phase II/Recruiting	Finland	NCT01938261
	Congenital Heart Defects/Tetralogy of Fallot	DRUG: recombinant human brain natriuretic peptide /DRUG: Placebo (0.9% sodium chloride)	2013-09-01	NA/Completed	China	NCT01941576
	Patent Ductus Arteriosus/Surgery/Necrotizing Enterocolitis/Intestinal Perforation	OTHER: pharmacologic treatment of the PDA/OTHER: no pharmacologic treatment of the PDA/DRUG: NSAID	2013-12-01	Phase II/Completed	United States	NCT01958320
	Tetralogy of Fallot/Heart Defects, Congenital/Ventricular Dysfunction, Right	DRUG: Losartan/DRUG: Placebo	2013-12-01	Phase II/Unknown	Netherlands	NCT02010905
	Congenital Heart Disease	DRUG: continuous infusion/DRUG: as needed dosing/DRUG: Acetaminophen/DRUG: ketorolac	2014-06-01	NA/Completed	United States	NCT02112448
	Ductus Arteriosus, Patent	DRUG: Paracetamol/DRUG: Placebo	2014-08-01	Phase II&III/Completed	Israel	NCT02002741
	Alagille Syndrome	DRUG: LUM001 (Maralixibat)/DRUG: Placebo	2014-10-28	Phase II/Completed	France/ Belgium/ Australia/ Spain/ United Kingdom	NCT02160782
	Congenital Heart Defects	Drug: Triostat/Drug: Placebo	2014-11-01	Phase III/Completed	United States	NCT02320669
	Alagille Syndrome	DRUG: LUM001/DRUG: Placebo	2014-11-24	Phase II/Completed	United States	NCT02057692
	Congenital Heart Diseases	DRUG: Ulinastatin	2015-04-01	Phase IV/Unknown	China	NCT02527811
	Patent Ductus Arteriosus	DRUG: ibuprofen/DRUG: paracetamol	2015-06-01	Phase IV/Unknown		NCT03265782
	Hypoplastic Left Heart Syndrome/Hypoplastic Right-sided Heart Complex	Drug: Ambrisentan/Other: Placebo	2015-07-01	Phase II/Completed	United States	NCT02080637
	Congenital Heart Disease	DRUG: Histidine Tryptophan Ketoglutarate Solution/OTHER: Terminal Warm Blood Cardioplegia	2015-12-01	Phase II/Unknown	Indonesia	NCT02618824
	Ductus Arteriosus, Patent	DRUG: Paracetamol/DRUG: Ibuprofen	2015-12-01	Phase II/Completed	Italy	NCT02422966
	Heart Defects, Congenital	DRUG: pH Stat	2016-03-01	NA/Unknown	United Kingdom	NCT02358382
	Analgesia/Congenital Heart Disease/Surgery	DRUG: paracetamol/DRUG: Morphine	2016-03-09	NA/Completed		NCT05853263
	Congenital Heart Disease	DRUG: Custodiol Solution/DRUG: Blood cardioplegia	2016-03-16	Phase III/Completed	Saudi Arabia	NCT03082716
	Bicuspid Aortic Valve	DRUG: Atorvastatin/DRUG: Placebo	2016-06-01	Phase III/Completed	Spain	NCT02679261
	Patent Ductus Arteriosus	DRUG: Paracetamol drops/OTHER: Placebo	2016-06-01	Phase II/Unknown	Israel	NCT02819414
	Heart Valve Disease/Heart Septal Defects, Atrial	DRUG: Dexmedetomidine/DRUG: Ketofol	2016-06-01	Phase IV/Completed	India	NCT02867930
	Single Ventricle Heart Disease	DRUG: Udenafil/DRUG: Placebo	2016-07-22	Phase III/Completed	United States/Canada	NCT02741115
	Ventricular Septal Defect	DRUG: Salbutamol/DRUG: Norflouran (Placebo Evohaler(R))	2016-10-05	Phase IV/Completed	Denmark	NCT02914652
	Coarctation of Aorta	DRUG: Dexamethasone/DEVICE: ultrasound/PROCEDURE: paravertebral block/DRUG: Bupivacaine/OTHER: isotonic saline	2016-10-30	Phase I/Completed	Egypt	NCT03074773
	Single Ventricle/Fontan	DRUG: Carvedilol/DRUG: Placebo	2016-11-01	Phase IV/Completed	United States	NCT02946892
	Patent Ductus Arteriosus	DRUG: Ibuprofen/OTHER: Expectative Management/DRUG: Indomethacin	2016-12-23	NA/Completed	Belgium/Netherlands	NCT02884219
	Patent Ductus Arteriosus	Drug: Acetaminophen/DRUG: Ibuprofen	2017-01-01	NA/Completed	United States	NCT03008876
	Noonan Syndrome	DRUG: Simvastatin/DRUG: Placebo	2017-01-25	Phase III/Completed	France	NCT02713945
	Persistent Ductus Arteriosus	DRUG: Ibuprofen in continuous (24 hours) iv infusion and EchoG/DRUG: IV bolus Ibuprofen slow (15 minutes) and EchoG	2017-02-20	Phase III/Unknown	Spain	NCT04282941
	Circulatory Perfusion Disorder/Congenital Heart Disease/Single-ventricle	DRUG: Vasopressin, Arginine/DRUG: Placebo	2017-03-06	Phase II&III/Completed	United States	NCT03088345
	Congenital Heart Disease	DRUG: Dexmedetomidine	2017-05-20	Phase II/Unknown	Egypt	NCT03425734
	Patent Ductus Arteriosus After Premature Birth	DRUG: Paracetamol/DRUG: Ibuprofen	2017-07-07	Phase III/Unknown	Spain	NCT04037514
	Congenital Heart Disease	DRUG: Macitentan 10 mg/DRUG: Placebo	2017-08-14	Phase III/Completed	United States/Australia/China/Denmark/ Germany/France/New Zealand/ United Kingdom	NCT03153137
	Congenital Heart Disease in Children/Inflammatory Response	DRUG: Methylprednisolone/DRUG: Isotonic saline	2017-10-18	Phase III/Completed	United States	NCT03229538
	Heart Defects, Congenital/Transposition of Great Vessels with Ventricular Inversion	DRUG: Tadalafil 20 Mg/DRUG: Placebo 20 Mg	2017-10-25	Phase III/Completed	Switzerland	NCT03049540
	Congenital Heart Disease/Oral Aversion	PROCEDURE: Endotracheal intubation	2018-07-01		United States	NCT05378685
	Congenital Heart Defects	DRUG: Sevoflurane/DRUG: TIVA	2018-08-20	Phase IV/Unknown	Brazil	NCT03630796
	Ductus Arteriosus, Patent	DRUG: Rectal Solution/DRUG: Intravenous Infusion	2018-09-01	Phase II&III/Unknown	Israel	NCT03604796
	Patent Ductus Arteriosus	Drug: Paracetamol 10Mg/mL/Drug: 0.45% Sodium Chloride/ Drug: Ibuprofen/ Drug: Indomethacin	2018-09-03	Phase I/Recruiting	Finland	NCT03648437
	Low Cardiac Output Syndrome/Cardiac Surgical Procedures/Infant	DRUG: Milrinone/DRUG: Normal saline	2019-02-01	Phase III/Unknown	China	NCT03823781
	Congenital Heart Disease in Children/Neuroprotection	DRUG: Allopurinol/DRUG: Mannitol	2020-02-14	Phase III/Recruiting	Netherlands	NCT04217421
	Patent Ductus Arteriosus	DRUG: Ibuprofen/DRUG: Paracetamol/OTHER: Expectant Management	2019-02-15	NA/Completed	Ukraine	NCT03860428
	Congenital Heart Disease/Upper Gastrointestinal Bleeding/Stress Ulcer/Infection	DRUG: Famotidine/DRUG: Placebo	2019-03-10	Phase IV/Completed	United States	NCT03667703
	Congenital Heart Disease	DRUG: Dexmedetomidine/DRUG: Normal saline	2020-08-25	NA/Unknown	Korea	NCT04484922
	Myocardial Injury/Cardiac Surgery	DRUG: Cyclosporin/PROCEDURE: remote ischemic preconditioning/DRUG: Placebo	2020-09-01	NA/Completed	Indonesia	NCT05691764
	Aortic Coarctation	DRUG: Saline Solution/DRUG: Levosimendan/DRUG: Magnesium Sulfate	2021-02-04	Phase IV/Completed	Egypt	NCT04330755
	Congenital Heart Disease in Children	DRUG: Continuous ketorolac	2021-03-01	Phase IV/Recruiting	United States	NCT04040452
	Alagille Syndrome	DRUG: Odevixibat/DRUG: Placebo	2021-03-19	Phase III/Completed	United States/Canada/France/ Germany/Israel/Italy/Malaysia/ Netherlands/New Zealand/Poland/ Turkey/United Kingdom	NCT04674761
	Patent Ductus Arteriosus After Premature Birth/Patent Ductus Arteriosus Conservative Management	Drug: Ibuprofen oral suspension/Drug: Placebo	2021-04-15	Phase II/Completed	Egypt	NCT05493540
	Patent Ductus Arteriosus	DRUG: Paracetamol injection/DRUG: Ibuprofen injection	2021-09-03	Phase II&III/Recruiting	United Kingdom	NCT04986839
	Congenital Heart Disease in Children/Cardiopulmonary Bypass	DRUG: Placebo/DRUG: Dexmedetomidine Hcl 100 Mcg/mL Inj/DRUG: Dexmedetomidine Hcl 100 Mcg/mL Inj	2021-12-16	Phase II&III/Completed	Indonesia	NCT05300802
	Patent Ductus Arteriosus After Premature Birth	DRUG: Ibuprofen	2022-01-10	Phase III/Recruiting	United States/Canada	NCT05011149
	22q11 Deletion Syndrome	DRUG: NB-001/OTHER: Placebo	2022-02-10	Phase II/Completed	United States/Canada	NCT05290493
	Congenital Heart Disease/Congenital Heart Defect/Congenital Heart Malformations	DRUG: Nitric Oxide 20 part per million/OTHER: Standard of care cardiopulmonary bypass	2022-04-25	Phase II&III/Enrolling by invitation	United States	NCT05101746
	Patent Ductus Arteriosus After Premature Birth	DRUG: Standard Dose Ibuprofen/DRUG: High Dose Ibuprofen	2022-06-01	Phase IV/Recruiting	Canada	NCT05325177
	Eisenmenger Syndrome	DRUG: Pentoxifylline	2022-06-03	NA/Recruiting	Brazil	NCT05611268
	Ventricular Septal Defect/Atrioventricular Septal Defect/Primum Atrial Septal Defect	DRUG: L-citrulline/DRUG: Plasmalyte A	2022-06-29	Phase III/Recruiting	United States	NCT05253209
	Congenital Heart Disease in Children/Cardiopulmonary Bypass/Tetralogy of Fallot	DRUG: Dexmedetomidine Hcl 100 Mcg/mL Inj/DRUG: Placebo	2022-10-10	Phase II&III/Completed	Indonesia	NCT05579964
	Patent Ductus Arteriosus After Premature Birth	Drug: Acetaminophen Injection/DRUG: Ibuprofen 20 Mg/mL oral suspension or Ibuprofen lysine 10 Mg/mL injection solution (Neoprofen)/OTHER: Sodium chloride 0.9% injection	2022-12-12	Phase II/Recruiting	Canada	NCT05340582
	Tetralogy of Fallot/Double Outlet Right Ventricle	Other: N-thymidine/Other: Urine Collection/Procedure: Echocardiogram/Procedure: Cardiac MRI/Drug: Propranolol Hydrochloride/Drug: Placebo/Procedure: Physical Exam/Other: Specimen Collection	2022-12-16	Phase I/Recruiting	United States	NCT04713657
	Cardiac; Dysrhythmia, Postoperative/Congenital Heart Surgery	DRUG: Dexmedetomidine/DRUG: Magnesium Sulfate/DRUG: Amiodarone/DRUG: Procainamide	2023-01-01	Phase IV/Unknown		NCT04234906
	Heart Defects, Congenital	DRUG: DEX group/OTHER: Control group	2023-01-05	Phase IV/Not yet recruiting		NCT05369949
	Congenital Heart Disease/Heart Failure/Heart Failure with Reduced Ejection Fraction	DRUG: Sacubitril 49 Mg / Valsartan 51 Mg [Entresto] BID/DRUG: Empagliflozin 10 Mg OD	2023-02-06	Phase II&III/Not yet recruiting	Mexico	NCT05580510
	Single Ventricle Heart Disease	DRUG: Udenafil/DRUG: Placebo	2023-10-30	Phase III/Recruiting	United States	NCT05918211
	Patent Ductus Arteriosus	DRUG: Paracetamol/DRUG: Ibuprofen	2023-12-01	Phase II/Not yet recruiting		NCT06152796
	Adult Congenital Heart Disease/Heart Failure	DRUG: Empagliflozin 10 Mg/DRUG: Placebo	2024-02-01	Phase IV/Not yet recruiting	United States	NCT06260059
	Patent Ductus Arteriosus	DRUG: Paracetamol	2024-03-01	Phase I&II/Not yet recruiting		NCT06256211
	Coarctation of Aorta/High Blood Pressure	DRUG: Losartan/DRUG: Amlodipine/DRUG: Placebo	2024-04-01	Phase III/Recruiting	United States	NCT06150560
	Congenital Heart Disease/Thiamine Deficiency/Patent Ductus Arteriosus/Ventricular Septal Defect/Atrial Septal Defect	DRUG: Thiamine	2024-05-01	Phase I/Recruiting	Indonesia	NCT06298344
Device treatment	Congenital Heart Defects	Device: Implantation and testing of CRT	2007-05-01	Phase II & III/ Completed	United States/Germany	NCT00450684
	Congenital Heart Disease	OTHER: Biventricular pacing	2007-12-01	NA/Completed	Canada	NCT02806245
	Hypoplastic Left Heart Syndrome	DEVICE: Peritoneal dialysis	2010-09-01	NA/Completed	Canada	NCT01215240
	Congenital Heart Defect/Surgery-Induced Tissue Adhesions/Hemorrhage	DEVICE: CoSeal Surgical Spray Group	2011-08-01	Phase II/ Completed	United States	NCT01330433
	Secundum Atrial Septal Defects	DEVICE: transcatheter closure of secundum atrial septal defects in patients	2012-05-21	NA/Completed		NCT04488120
	Congenital Heart Disease	DEVICE: Biventricular Pacing	2012-07-01	NA/Completed	Canada	NCT02644824
	Congenital Heart Disease	DEVICE: Use of high flows versus oxygen therapy/DEVICE: oxygen therapy	2012-08-01	NA/Unknown	Italy	NCT01633801
	Heart Septal Defects, Ventricular/Double Outlet Right Ventricle, Noncommitted VSD/Double Outlet Right Ventricle, Subaortic VSD/Double Outlet Right Ventricle, Subpulmonary VSD/Supracristal Ventricular Septal Defect	DEVICE: TEE-guided perventricular device closure without CBP/PROCEDURE: Surgery repair with CBP	2012-12-01	NA/Completed	China	NCT02361008
	Congenital Heart Defects	DEVICE: Warfarin Dosing Aid/OTHER: Standard Practice	2015-08-01	NA/Unknown		NCT02475863
	Mitral Valve Insufficiency/Heart Septal Defects, Atrial	DEVICE: Figulla Flex Occluder (Occlutech)	2016-01-01	NA/Unknown	Germany	NCT03024268
	Congenital Heart Defect	DEVICE: electro-acupuncture /DEVICE: sham	2017-11-15	NA/Unknown	United States	NCT03297658
	Atrial Septal Defect	DEVICE: Guidewire for echo-guided interventions/DEVICE: Cook lunderquist guidewire	2018-07-07	NA/Unknown	China	NCT04096924
	Ventricular Septal Defect	DEVICE: Fully Absorbable VSD Occlusion System/DEVICE: VSD Occluder	2019-04-11	NA/Unknown	China	NCT03941691
	Aortic Stenosis/Aortic Stenosis with Bicuspid Valve	DEVICE: TAVR with Venus A plus using supra-annular sizing and THV implantation technique (Hangzhou solution)/DEVICE: TAVR with Venus A plus using annular sizing and THV implantation technique	2021-04-12	NA/Recruiting	China	NCT04722796
	Atrial Septal Defect	DEVICE: ASD closure with the novel occluder/DEVICE: ASD closure with normal occluder	2022-04-22	NA/Unknown	China	NCT05371366
	Congenital Heart Disease in Children	DEVICE: Ductal Arterial Stent/PROCEDURE: Systemic-to-Pulmonary Artery Shunt	2022-06-02	NA/Recruiting	United States/Canada	NCT05268094
	Congenital Heart Disease/Systemic Right Ventricle/Congenitally Corrected Transposition of the Great Arteries/Transposition of Great Vessels/Heart Failure Congenital	DEVICE: CRT ON (biventricular pacing) / CRT OFF (inactive or univentricular pacing)/DEVICE: CRT OFF (inactive or univentricular pacing) / CRT ON (biventricular pacing)	2022-09-01	NA/ Active yet recruiting	France	NCT05524324
	Ductus Arteriosus, Patent	DEVICE: Percutaneous Patent Ductus Arteriosus Closure / COMBINATION_PRODUCT: Responsive Management Intervention/DIAGNOSTIC_TEST: Echocardiogram, cardiac	2023-02-21	NA/Recruiting	United States	NCT05547165
	Congenital Heart Disease	DEVICE: method comparison	2023-03-17	NA/Recruiting	United States	NCT06078943
Biological treatment	Hypoplastic Left Heart Syndrome/Single Right Ventricle/Single Left Ventricle	GENETIC: Cardiac progenitor cell infusion	2013-04-01	Phase II/ Completed	Japan	NCT01829750
	Congenital Heart Disease	BIOLOGICAL: Fibrinogen Concentrate/BIOLOGICAL: Cryoprecipitate	2016-03-01	Phase IV/ Completed	United States	NCT03014700
	Turner Syndrome	BIOLOGICAL: PEG-rhGH low dose/BIOLOGICAL: PEG-rhGH high dose/OTHER: Non-treatment control group	2016-03-01	Phase II/Unknown	China	NCT03189160
	Hypoplastic Left Heart Syndrome/Single Ventricle	GENETIC: Autologous cardiac stem cells		Phase III/Recruiting	Japan	NCT02781922
	Patent Ductus Arteriosus	BIOLOGICAL: Liberal platelet transfusion/BIOLOGICAL: Restrictive platelet transfusion/DRUG: Paracetamol/DRUG: Ibuprofen	2016-03-01	Phase III/ Completed	India	NCT03022253
	Congenital Heart Disease/Total Cavo-pulmonary Connection	DRUG: Nesiritide/DRUG: Normal saline	2017-07-05	NA/Unknown	China	NCT03207295
	Hypoplastic Left Heart Syndrome/Atrioventricular Canal	Biological: MPC; rexlemestrocel-L	2017-11-27	Phase I/Active not recruiting	United States	NCT03079401
	Hypoplastic Left Heart Syndrome	BIOLOGICAL: Longeveron Mesenchymal Stem Cells	2018-02-21	Phase I&II/Active not recruiting	United States	NCT03525418
	Congenital Heart Disease	BIOLOGICAL: Platelet transfusion/BIOLOGICAL: Fibrinogen concentrate	2018-03-14	Phase II/Unknown	Sweden	NCT04807621
	Hypoplastic Left Heart Syndrome	BIOLOGICAL: c-kit^+^ cells	2019-10-16	Phase I/Recruiting	United States	NCT03406884
	Hypoplastic Left Heart Syndrome	Biological: Lomecel-B medicinal signaling cells	2021-06-25	Phase II/Recruiting	United States	NCT04925024
	Turner Syndrome	BIOLOGICAL: Lonapegsomatropin/DRUG: Somatropin	2023-02-15	Phase II/Active not recruiting	United States	NCT05690386
Behavioral treatment	Heart Defects, Congenital	BEHAVIORAL: Physical Activity/BEHAVIORAL: Education	2006-08-01	Phase III/ Completed	Canada	NCT00363363
	Heart Defects, Congenital	BEHAVIORAL: Motorpedagogic exercise	2007-02-01	NA/Unknown	Germany	NCT00436098
	Transposition of Great Vessels	BEHAVIORAL: Training	2009-02-01	NA/Completed	Germany	NCT00837603
	Congenital Heart Defects	BEHAVIORAL: Clinic-based Educational Intervention	2011-01-01	NA/Completed	Canada	NCT01286480
	Congenital Heart Disease/Post Cardiac Surgery	BEHAVIORAL: Daily Messages, Virtual Home Visits/OTHER: Usual Care	2012-07-01	NA/Completed	United States	NCT01941667
	Congenital Heart Defects	BEHAVIORAL: Home based interval training	2012-09-01	NA/Completed	Sweden	NCT01671566
	Congenital Heart Disease	BEHAVIORAL: Educational/BEHAVIORAL: Self-management	2012-12-01	NA/Completed	Canada	NCT01723332
	Heart Defects, Congenital	BEHAVIORAL: ACHD-CARE Program	2013-06-01	NA/Completed	Canada	NCT01881893
	Congenital Heart Defect	BEHAVIORAL: home-based exercise training	2015-01-01	NA/Completed	Belgium	NCT02240147
	Communication/Heart Defects, Congenital/Infant Conditions	BEHAVIORAL: Guided Participation	2015-02-25	NA/Completed	United States	NCT04452201
	Heart Defects, Congenital	BEHAVIORAL: CHAPTER III Study Intervention	2015-03-01	NA/Completed	Canada	NCT02374892
	Congenital Heart Disease	BEHAVIORAL: Developmental recommendations/ BEHAVIORAL: Standard care	2016-01-05	NA/Completed	United States	NCT02700646
	Congenital Heart Defect	BEHAVIORAL: Home based resistance training	2016-02-01	NA/Unknown	Sweden	NCT02658266
	Congenital Heart Disease	BEHAVIORAL: exercise training	2016-02-01	NA/Unknown	Netherlands	NCT02825472
	Congenital Heart Disease/Neurodevelopment/Executive Function/Working Memory Training/Infant Open-heart Surgery	BEHAVIORAL: Cogmed Working Memory Training	2016-06-01	NA/Completed	United States	NCT02759263
	Heart Defects, Congenital	BEHAVIORAL: Pacifier activated music player	2016-09-15	NA/Completed	United States	NCT03035552
	Congenital Heart Defect/Executive Function/Children/Neurodevelopmental Disorders/Working Memory/Infant Open-Heart Surgery	BEHAVIORAL: Cogmed Working Memory Training	2017-02-21	NA/Completed	United States	NCT03023644
	Fragile X Syndrome/Williams Syndrome	BEHAVIORAL: Cooperative Parent Mediated Therapy /BEHAVIORAL: As usual	2017-05-17	NA/Unknown	Italy	NCT04610424
	Cardiovascular Disease Other/Physical Activity	BEHAVIORAL: Physical Activity Lifestyle Intervention/BEHAVIORAL: Physical Activity Monitoring	2017-11-07	NA/Completed	United States	NCT03335475
	Heart Failure/Congenital Heart Disease	BEHAVIORAL: Respiratory muscle training	2018-02-15	NA/Completed	Switzerland	NCT03297918
	Congenital Heart Disease/Heart; Surgery, Heart, Functional Disturbance as Result	BEHAVIORAL: Teaching session/BEHAVIORAL: Just TRAC It! /BEHAVIORAL: MyHealth Passport	2018-04-17	NA/Completed	Canada	NCT03429335
	Heart Defects, Congenital	BEHAVIORAL: REMOTE-CR/OTHER: Control	2019-02-03	NA/Recruiting	Sweden	NCT03479957
	Congenital Heart Defects	BEHAVIORAL: Psychoeducational intervention	2019-03-07	NA/Completed	Portugal	NCT03724006
	Cardiovascular Disease Other/Physical Activity	BEHAVIORAL: Physical Activity Lifestyle Intervention/BEHAVIORAL: Physical Activity Monitoring	2019-03-14	NA/Completed	United States	NCT04135859
	Congenital Heart Disease/Congenital Heart Defect	BEHAVIORAL: Peer Health Coaching	2020-10-08	NA/Active not recruiting	United States	NCT04271358
	Congenital Heart Disease/Child Development/Early Intervention	BEHAVIORAL: Remotely monitored parent-mediated hybrid home and clinic based multidisciplinary Early Intervention protocols.	2021-03-16	NA/Recruiting	Brazil	NCT05907109
	Congenital Heart Disease	BEHAVIORAL: Transition care model	2021-05-03	NA/Recruiting	Italy	NCT05713591
	Congenital Heart Disease/Psychological Intervention/Parenting/Quality of Life/Psychological Disturbance	BEHAVIORAL: problem prevention therapy with congenital heart disease	2021-05-07	NA/Unknown	Pakistan	NCT05109806
	Down Syndrome	BEHAVIORAL: Combined exercise intervention/BEHAVIORAL: Usual care / Sham intervention	2021-09-09	NA/Recruiting	United States	NCT04854122
	Congenital Heart Disease/Congenital Heart Disease in Adolescence	BEHAVIORAL: Use of the congenital heart disease app/BEHAVIORAL: Nurse-led intervention	2022-01-10	NA/Recruiting	Canada	NCT04463446
	Congenital Heart Disease/Congenital Heart Defects	BEHAVIORAL: Cardiac rehabilitation	2022-02-07	NA/Enrolling by invitation	Canada	NCT05195788
	Congenital Heart Disease	Behavioral: Promoting Resilience in Stress Management/Other: Usual Care	2023-03-15	NA/Recruiting	United States	NCT04738474
	Heart Defects, Congenital/Breast Feeding	OTHER: Conventional care/BEHAVIORAL: Breastfeeding behavioral intervention for mothers of infants with congenital heart disease	2023-05-25	NA/Recruiting	China	NCT05961540
	Congenital Heart Disease in Children	BEHAVIORAL: Remote Cardiac Rehabilitation/ BEHAVIORAL: Active Control	2023-08-31	NA/Recruiting	United States	NCT06015191
	Congenital Heart Disease/Child Development/Neurodevelopmental Disorders/Parents	BEHAVIORAL: Parent-child yoga	2023-10-01	NA/Recruiting	Canada	NCT05997680
	Cardiology/Infant Development/Development Delay	BEHAVIORAL: NIDCAP Developmental Care	2023-10-10	NA/Recruiting	United States	NCT05885113
	22Q11 Deletion Syndrome	BEHAVIORAL: Aware Program	2023-12-18	NA/Recruiting	United States	NCT05849441
	Congenital Heart Disease	BEHAVIORAL: I-InTERACT-North	2024-02-15	NA/Recruiting	Canada	NCT06075251
	Congenital Heart Disease/Executive Dysfunction/Attention Disorder	Behavioral: Kleuter Extra training program	2024-03-01	NA/Not yet recruiting	Netherlands	NCT05885113
	Congenital Heart Disease/Executive Dysfunction/Attention Disorder	BEHAVIORAL: Kleuter Extra training program	2024-03-01	NA/Not yet recruiting	Netherlands	NCT06267430
	Heart Failure Congenital/Single-ventricle	BEHAVIORAL: Cardiac rehabilitation/OTHER: Usual care	2024-05-05	NA/Recruiting	United States	NCT06150950
	Transition to Adult Care/Congenital Heart Disease	BEHAVIORAL: Get Ready with My Heart Program/BEHAVIORAL: Conventional intervention	2024-06-01	NA/Not yet recruiting	China	NCT06418373
	Congenital Heart Disease in Adolescence	BEHAVIORAL: Transitional care training with brochures, posters and slide training materials	2024-06-17	NA/Not yet recruiting	Turkey	NCT06433401
Other treatments	Adults With Congenitally Malformed Hearts	OTHER: Physician and nurse consultation	2006-05-01	NA/Completed		NCT01234753
	Congenital Heart Disease	OTHER: transfusion strategy/OTHER: Low Hb transfusion group	2006-07-01	Phase II/Completed	United States	NCT00350220
	Fontan Procedure/Hypoplastic Left Heart Syndrome/Tricuspid Atresia	DIETARY_SUPPLEMENT: Vitamin C / DIETARY_SUPPLEMENT: Placebo	2009-06-01	NA/Completed	United States	NCT00974025
	Congenital Heart Disease	DIETARY_SUPPLEMENT: Standard protein delivery/DIETARY_SUPPLEMENT: Intervention 1 (2.2 g/kg/day)/DIETARY_SUPPLEMENT: Intervention 2 (3.0 g/kg/day)	2009-08-01	Phase IV/Completed	Canada	NCT01368705
	Heart Defects, Congenital	OTHER: NIRS based management/OTHER: Control	2011-11-01	NA/Completed	United States	NCT02157597
	Impaired Oxygen Delivery/Congenital Heart Disease	OTHER: Red blood cell transfusion/OTHER: Red blood cell transfusion	2012-01-01	NA/Completed	United States	NCT01484886
	Congenital Heart Disease/Single Ventricle/Univentricular Heart	OTHER: Aerobic Exercise Training/OTHER: Inspiratory Muscle Training/OTHER: No Exercise Training	2013/1/31	NA/Completed	Brazil	NCT02283255
	Congenital Heart Disease/Heart Defects, Congenital	OTHER: Cardiopulmonary rehabilitation/OTHER: Standard of care	2013-04-01	NA/Completed	United States	NCT01822769
	Congenital Heart Disease	OTHER: STAGES-Booklet	2013-06-01	NA/Completed	Canada	NCT01909583
	Tetralogy of Fallot	OTHER: Exercise training	2015-06-01	NA/Completed	Slovenia	
	Congenital Heart Disease	BEHAVIORAL: MyHeartBaby Program	2015-07-01	NA/Completed	United States	NCT02895334
	Congenital Heart Disease	OTHER: Osteopathy/OTHER: superficial palpatory agreement	2016-05-01	NA/Completed	France	NCT02710825
	Heart Defects, Congenital	OTHER: Fontan Education Videos	2016-07-01	NA/Completed	Canada	NCT02831790
	Congenital Heart Disease in Children/Malnutrition, Child	DIETARY_SUPPLEMENT: High-calorie density formula (1 kcal/ml)/DIETARY_SUPPLEMENT: Standard formula (0.67 kcal/ml)	2018-02-14	NA/Completed	Indonesia	NCT05945459
	Aortic Valve Stenosis/Bicuspid Aortic Valve	DIETARY_SUPPLEMENT: Vitamin K2/OTHER: Placebo	2016-08-01	Phase II/Unknown	Netherlands	NCT02917525
	Congenital Heart Disease	DIETARY_SUPPLEMENT: Nutritional perioperative prehabilitation program for two weeks/DIETARY_SUPPLEMENT: Nutritional perioperative prehabilitation program for one week	2017-11-01	NA/Completed	Egypt	NCT02475759
	Patent Ductus Arteriosus After Premature Birth/Hyper Bilirubinemia	OTHER: Group 1: non chest shielding/OTHER: Group 2: chest shielding	2018-07-01	NA/Unknown	Turkey	NCT03675425
	Heart Defects, Congenital/Adolescent/Young Adult	OTHER: Cardiac rehabilitation	2018-07-27	NA/Completed	France	NCT03690518
	Infant ALL/Congenital Heart Disease	OTHER: Control/OTHER: Experimental Group (Absorption Group)/OTHER: Experimental Group (Milking Group)	2018-07-28	NA/Completed	Turkey	NCT04454294
	Congenital Heart Disease/Transition/Pediatric Congenital Heart Disease	BEHAVIORAL: Web-based Educational Intervention	2018-08-27	NA/Active not recruiting	United States	NCT03303248
	Infant, Premature/Patent Ductus Arteriosus/Infant, Newborn, Diseases/Patent Ductus Arteriosus After Premature Birth	OTHER: Active Treatment/OTHER: Expectant Management	2018-12-03	Phase III/Recruiting	United States	NCT03456336
	Congenital Heart Disease	OTHER: Sophrology sessions/OTHER: usual care	2019-07-19	NA/Completed	France	NCT03999320
	Patient Decision Aids/Congenital Heart Disease/Congenital Heart Defect	OTHER: Decision Aid/OTHER: Values Clarification Exercise	2020-10-01	NA/Recruiting	United States	NCT04437069
	Tetralogy of Fallot/Hypoplastic Left Heart Syndrome/Univentricular Heart/Heart Defects, Congenital	OTHER: Neurally adjusted ventilatory assist first/OTHER: Conventional ventilation first	2020-10-05	NA/Recruiting	Canada	NCT04581668
	Congenital Heart Defect	OTHER: Individualized Home and Play-Based Physical Activity Plans	2020-11-1	NA/Recruiting	Canada	NCT04619745
	Mother-Infant Interaction	OTHER: infant massage practice/OTHER: Safe Swaddling/OTHER: Control	2020-12-01	NA/Active yet recruiting	Turkey	NCT06158373
	Marfan Syndrome	OTHER: Endurance training/OTHER: Muscle building training	2021-01-04	NA/Completed	France	NCT04553094
	Hypoplastic Left Heart Syndrome/Total Anomalous Pulmonary Venous Return/Truncus Arteriosus/Pulmonary Atresia with Ventricular Septal Defect/Transposition of the Great Arteries/Double Outlet Right Ventricle, Subpulmonary VSD/Tetralogy of Fallot/Double Outlet Right Ventricle with Subaortic Ventricular Septal Defect and Pulmonary Stenosis/Cardiopulmonary Bypass	OTHER: Normoxia (with controlled re-oxygenation)/OTHER: Standard of care ventilation	2021-01-18	NA/Not yet recruiting	United States	NCT04452188
	Congenital Heart Disease	OTHER: passive range of motion exercise therapy	2021-02-23	Phase III/Recruiting	United States	NCT04702373
	Congenital Heart Disease	DIETARY_SUPPLEMENT: Infant formula	2021-04-03	NA/Completed	Iran	NCT04795076
	Congenital Heart Disease in Children	OTHER: early motor intervention	2021-05-31	NA/Completed	Switzerland	NCT04666857
	Congenital Heart Disease in Children/Stress, Psychological	OTHER: Access to mobile phone application/OTHER: Treatment as usual	2021-06-01	NA/Recruiting	Norway	NCT04315610
	Congenital Heart Disease in Children/Stress, Psychological	Other: Access to mobile phone application/Other: Treatment as usual	2021-06-01	NA/Recruiting	Norway	NCT04315610
	Congenital Heart Disease in Children/Post-cardiac Surgery/Malnutrition, Infant	DIETARY_SUPPLEMENT: Energy- Protein Enriched Nutritional Formula	2021-07-21	NA/Recruiting	Saudi Arabia	NCT05826769
	Mothers/Quality of Life/Self Efficacy/Training	OTHER: Web-Based Education Program	2022-02-01	NA/Completed	Turkey	NCT06168344
	Congenital Heart Disease	OTHER: Weaning from mechanical ventilation	2022-04-15	NA/Completed	Egypt	NCT05344872
	Congenital Heart Disease	OTHER: Conventional treatment/OTHER: Limb Range of Motion Exercises + Chest Physical Therapy	2022-06-20	NA/Completed	Pakistan	NCT05425173
	Breastfeeding/Congenital Heart Disease	OTHER: Breastfeeding	2022-07-01	NA/Recruiting	Brazil	NCT06025864
	Congenital Heart Disease/Emotional Regulation	OTHER: CHD-specific web-based emotion regulation intervention/OTHER: General web-based emotion regulation intervention	2022-08-05	NA/Recruiting	Germany	NCT05862909
	Congenital Heart Disease/Mother Child Interaction	OTHER: Creative Music Therapy	2022-11-22	NA/Recruiting	Switzerland	NCT05702203
	Congenital Heart Defects/Underweight	DIETARY_SUPPLEMENT: pre-surgical nutritional intervention protocol/OTHER: Control	2023-01-09	NA/Not yet recruiting		NCT05457712
	Quality of Life/Congenital Heart Disease/Heart; Surgery, Heart, Functional Disturbance as Result	OTHER: pre-operative exercise therapy group/OTHER: control group	2023-03-01	NA/Completed	Pakistan	NCT05763238
	Congenital Heart Disease	OTHER: blood use for priming cardiopulmonary bypass circuit/OTHER: clear prime for cardiopulmonary bypass	2023-05-10	NA/Recruiting	United States	NCT05881564
	Congenital Heart Disease	OTHER: Surgical Simulation with 3D Heart Model and Parental Education with “Congenital Heart Disease Parent Education Booklet” and tailored 3D Heart Modeling	2023-07-01	NA/Not yet recruiting	Turkey	NCT05852106
	Heart Defects, Congenital	OTHER: cardiac rehabilitation program/OTHER: TELEA platform	2024-01-01	NA/Not yet recruiting		NCT06185140
	Pulmonary Arterial Hypertension/Heart Defects, Congenital	OTHER: Training	2024-02-01	NA/Not yet recruiting	Turkey	NCT06172790

The data are up to 14 June 2024

*ACHD-CARE* adult congenital heart disease-coping and resilience, *app* application, *ASD* atrial septal defect, *BAV* bicuspid aortic valve, *CPB* cardiopulmonary bypass, *CR* cardiac rehabilitation, *CRT* cardiac resynchronization therapy, *DEX* dexmedetomidine, *D5W* dextrose 5% in water, *ERAS* enhanced recovery after surgery, *Hb* hemoglobin, *Hcl* hydrochloride, *I-InTERACT-North* a virtual mental health parenting stepped-care intervention, *Inj* injection, *IV* intravenous, *IVIG* intravenous immunoglobulin, IVMP intravenous methylprednisolone pulse, *Just TRAC It! study* transitioning responsibly to adult care using smartphone technology, *Ketofol* ketamine and propofol, *LUM* Lumena, *Mcg* microgram, *Mg* milligram, *mL* milliliter, *MPC* mesenchymal precursor cell, *MRI* magnetic resonance imaging, *NA* not applicable, *NIDCAP* newborn individualized developmental care and assessment program, *NIRS* near infrared reflectance spectroscopy, *NSAID* nonsteroidal anti-inflammatory drug, *PA* pulmonary artery, *PDA* patent ductus arteriosus, *PEG-rhGH* pegylated recombinant human growth hormone, *PVR* pulmonary vascular resistance, *RV* right ventricle, *SC TAP* subcostal transverse abdominis plane block, *TAVR* transcatheter aortic valve replacement, *TELEA* transurethral endoscopic laser ablation, *CHAPTER* congenital heart adolescents participating in transition evaluation research, *THV* transcatheter heart valve, *TIVA* total intravenous anesthesia, *VSD* ventricular septal defect

### Advances in therapeutic strategies for CHD

**Fig. 7:**
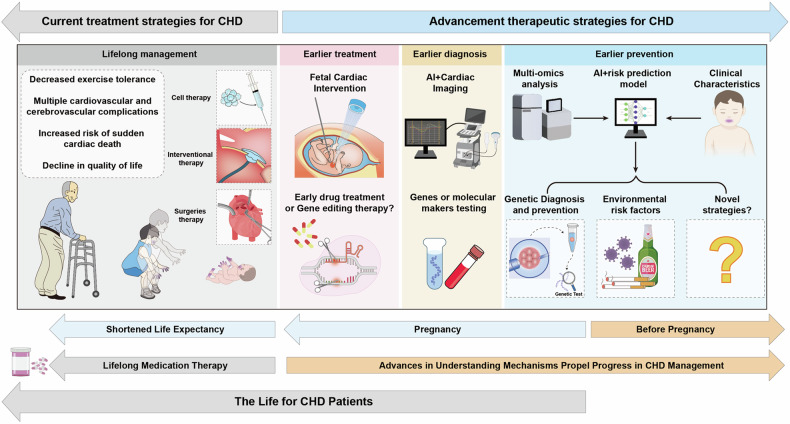
**Current and future advanced treatment strategies for CHD**. Currently, CHD is considered a lifelong condition and is primarily treated through early surgical interventions, multiple interventional catheterizations, and cell therapies. However, postsurgery CHD patients often face challenges such as decreased exercise tolerance, multiple cardiovascular and cerebrovascular complications, increased risk of sudden cardiac death, and decreased quality of life. Therefore, lifelong management, including long-term medication and care, is necessary for CHD patients. Advances in technology and a deeper understanding of cardiac development mechanisms aim to achieve earlier diagnosis and prevention of CHD, minimizing its impact and improving patient quality of life and longevity. Early intervention strategies, such as FCI in select CHD patients, are already underway, although further research into related drug therapies and gene treatments is needed. The development of cardiac imaging combined with AI technology enhances CHD diagnosis accuracy, while genetic testing aids in detecting pathogenic variants associated with CHD, facilitating early diagnosis. PGT offers the possibility for early prevention of CHD by identifying genetic defects or chromosomal abnormalities in embryos early in pregnancy; thus, selecting embryos most likely to result in successful pregnancies and healthy offspring is highly desirable. Utilizing AI to integrate multi-omics and clinical data for constructing risk prediction models will enable the identification of more precise genetic or environmental risk factors, further promoting early prevention strategies for CHD in the future. AI artificial intelligence, CHD congenital heart disease, FCI fetal cardiac intervention, PGT preimplantation genetic testing. This figure was created using Adobe Illustrator

Cell therapy has been explored for the treatment of several types of CHD, including the injection of autologous umbilical cord blood-derived cells in patients with HLHS,^631,632^ and the intracoronary delivery of autologous cardio-sphere-derived cells in patients with a single ventricle.^633^ These studies have shown improvements in cardiac function. However, such treatments currently serve only as adjuncts to surgical interventions for CHD patients. With an increased understanding of cardiac developmental mechanisms and technological advancements, it is hoped that future treatments will enable earlier diagnoses, early interventions, and preventive measures (Fig. 7). The advent of fetal cardiac intervention (FCI) represents a significant step toward modifying CHD before birth, potentially improving neonatal health outcomes. FCI is mainly used for conditions such as a restricted or intact atrial septum, severe aortic stenosis with evolving HLHS, severe mitral stenosis, and pulmonary atresia with an intact ventricular septum.^634^ The results are promising, with most children showing significant symptom improvement.^635,636^ However, due to the limited number of patients treated with FCI, the long-term effects of FCI on patients require further observation.

Currently, there are no targeted drugs or gene therapies specific to different types of CHD. This limitation is due to an incomplete understanding of the mechanisms of heart development and the immature nature of the technology. Targeted therapies, which act on specific molecules or genetic pathways to block or interfere with disease progression, are primarily used in oncology and autoimmune diseases, such as EGFR or VEGFR-tyrosine kinase inhibitors for non-small cell lung cancer, benefiting from an in-depth understanding of these diseases. A recent study used targeted drugs (phosphodiesterase type 5 [PDE-5] inhibitors and prostacyclin analogs) therapy to reduce pulmonary artery pressure in 13 pregnant women with Eisenmenger syndrome, thereby increasing maternal survival rates.^637^ However, this approach is currently limited to the treatment of adult CHD patients. The complex regulatory mechanisms of cardiac development make it challenging to develop targeted drug therapy for the early treatment of CHD. However, as our understanding of cardiac development mechanisms improves, targeted therapies may become promising treatment methods for CHD, potentially avoiding surgical interventions. The emergence of clustered, regularly interspaced short palindromic repeats/CRISPR-associated protein 9 (CRISPR/Cas9) technology has made gene editing therapy possible, but it has raised significant ethical concerns, such as whether humans should modify their genes. Additionally, the technology still needs to be mature. For instance, off-target effects may unintentionally alter genes outside the target sequence, making its future application uncertain.

Clinical genetic and genomic testing for genetic defects causing CHD can aid in early diagnosis, including chromosome analysis, chromosomal microarray, targeted gene sequencing/genetic testing panels, genomic testing with exome sequencing, genomic testing with genome sequencing, and mitochondrial genome sequencing.^638^ For example, one study applied whole exome sequencing to analyze 69 known CHD genes and identified likely pathogenic mutations in up to 33% of familial CHD patients, demonstrating the successful use of a CHD candidate gene list for rapid and early identification of likely pathogenic variants from large datasets.^639^ However, due to insufficient knowledge of pathogenic variants in most CHDs, genetic and genomic testing can only identify a small proportion of fetuses carrying genetic defects, highlighting significant limitations in early diagnosis. A greater understanding of cardiac developmental mechanisms is needed to enhance the application of genetic testing in early CHD diagnosis.

Preventing CHD remains challenging. Preimplantation genetic testing (PGT) during in vitro fertilization (IVF) can screen embryos for genetic abnormalities before implantation, identifying those most likely to result in a successful pregnancy and healthy offspring. These methods include PGT-A (aneuploidy screening), PGT-M (monogenic/single-gene disorder screening), and PGT-SR (structural rearrangement screening).^640^ However, there are several limitations: our understanding of pathogenic variants in CHD is incomplete, making full prevention difficult; this method is only applicable to IVF; it raises psychological and ethical considerations, such as eugenics and sex selection. In addition to genetic defects, many environmental factors contribute to CHD.^14^ While factors such as infections during pregnancy, diabetes, smoking, and alcohol consumption are known to be closely related to CHD, the specific mechanisms by which these risk factors lead to clinical phenotypes of CHD are still unclear. Therefore, while preventing the occurrence of CHD can reduce the need for complex surgeries and lifelong medical management, significantly enhancing the overall quality of life for affected individuals, our limited understanding of cardiac development mechanisms and incomplete knowledge of risk factors for most CHD mean that we can currently only prevent the occurrence of a small number of CHD cases.

### The application of AI for management of CHD

The development of AI technology is encouraging. AI has been applied in various aspects of CHD diagnosis, risk prediction and treatment, including analyzing cardiac imaging data to assist in diagnosing and monitoring CHD, assessing disease severity, and planning interventions.^641–643^ Advances in cardiac imaging and artificial intelligence (AI) technology have allowed us to understand fetal CHD development during pregnancy better.^644,645^ In prenatal diagnosis of CHD, AI has the potential to enhance detection rates by automatically extracting standard imaging planes from streams of ultrasound imaging data. One algorithm based on a deep learning framework evaluated its performance in automatically detecting and assessing the quality of cardiac four-chamber planes.^646^ A random forest algorithm was found to improve the sensitivity of prenatal CHD screening.^647^ Another study trained neural network models to differentiate between normal hearts and complex CHD, finding that the model’s sensitivity was comparable to that of clinicians and remained robust even with out-of-hospital or low-quality images.^648^ These algorithms can help clinicians detect CHD earlier, improve detection rates, and more accurately assess disease severity, thereby aiding in clinical decision-making. Future optimization of AI algorithms or development of learning algorithms specifically targeting fetal CHD could further enhance the detection of unique CHD.

The AI is also used to predict clinical outcomes by inputting clinical, imaging, and genomic data from CHD patients, particularly in the management of adult congenital heart disease (ACHD) patients.^649^ Today, 97% of children with CHD survive into adulthood, but the increased lifespan also brings a higher risk of acquired diseases, such as type 2 diabetes, hypertension, acquired cardiovascular diseases, and cancer. Managing the growing number of ACHD patients has become a challenge.^609,650^ Transferring ACHD patients to specialist ACHD centers is a reasonable approach. Staff at these centers typically include adult and pediatric cardiologists with ACHD certification, CHD surgeons, pulmonary vascular disease experts, clinical geneticists, and psychologists, who work together to develop the most appropriate management and follow-up plans for each patient.^610^ The development of AI technology also supports improving the quality of ACHD patient management. Risk stratification systems are central to managing ACHD patients. Traditional ACHD risk stratification systems are based on limited data from single or few institutions, while AI can facilitate the integration of larger or longitudinal datasets, better fitting the underlying data and thus improving predictive capabilities.^651^ Currently, several studies have applied deep learning algorithms based on natural language processing,^652^ cardiac magnetic resonance (CMR) data,^653^ and recurrent neural networks^654^ for risk stratification in ACHD patients, predicting outcomes such as heart failure, prognosis, and mortality. These studies suggest the feasibility of using AI models for risk stratification. AI-based algorithms are also helpful in predicting postoperative complications, disease progression, and treatment outcomes.^655^ For instance, in managing heart transplant patients, AI models can more accurately predict graft failure and mortality and promote positive behavioral changes to reduce future cardiovascular risks.^656^ AI-driven risk prediction models also assist in predicting individual mortality risks for CHD patients undergoing surgery and ICU length of stay, thereby improving care planning and resource management.^657,658^ In addition to enhancing early diagnosis of CHD, AI algorithms can analyze electrocardiograms (ECGs) and cardiac imaging data to precisely identify changes in characteristics, aiding in more accurate risk prediction. AI-based ECG evaluation can address the issue of limited data in certain CHD populations. For example, Mayourian and colleagues developed and validated an AI-ECG model for predicting biventricular dysfunction and dilation in ACHD patients, which reduces the frequency of echocardiograms and CMRs to lower costs and improve access to care.^659^ Another study significantly improved the accuracy of junctional ectopic tachycardia detection in CHD patients using deep neural networks, allowing for precise diagnosis and timely intervention for this life-threatening postoperative arrhythmia.^660^ Machine learning models based on cardiac CMR can also help predict deterioration in patients with repaired ToF.^661^

The combination of AI and imaging technology facilitates surgical treatment for CHD.^645^ For example, generative adversarial networks have been successfully used to predict the optimal size, shape, and position of transannular patches to improve cardiac CT outcomes for patients with ToF.^662^ A random forest model based on preoperative cardiac CT has been shown to predict which post-Glenn shunt patients are at low or high risk for a mean pulmonary arterial pressure (mPAP) > 15 mmHg, thereby reducing the need for right heart catheterization in these patients.^663^ Another study utilized a Cycle Generative Adversarial Network to align pre-procedural CTs with intra-procedural transesophageal echocardiographic images, improving surgical navigation for CHD patients.^664^ One significant advantage of AI is its ability to integrate and synthesize multi-layered medical data, including clinical information, imaging data, genetic information, and environmental factors, to provide personalized analysis and precision medicine. While congenital heart defects can be broadly classified as mild, moderate, or severe, more detailed classifications can exceed 30 types. This underscores the complexity of ACHD patients and importance of personalized treatment for them.^610^ Although AI technology in the field of CHD and healthcare is still in its early stages, it has already yielded many promising results. In the future, AI technology is expected to advance the development of personalized treatments and interventions for ACHD patients.

At the same time, applying AI algorithms to integrate multi-omics data and clinical information from CHD patients to identify genetic variations and environmental factors associated with CHD susceptibility and severity, and developing corresponding risk prediction models, could advance early prevention of CHD. A recent study combined metabolomics and a machine learning model to screen maternal serum metabolites, identifying a metabolomic fingerprint for CHD and providing a non-invasive screening method for early detection of the condition.^665^ Another study using AI and epigenomics found that DNA methylation could predict aortic coarctation in neonates.^666^ However, these studies have only applied single-omics and AI algorithms, and the full potential of AI technology has yet to be realized. In the future, AI technology, by integrating multi-layered big data, could identify more genetic and non-genetic risk factors for CHD, thereby advancing early prevention and diagnosis.

## Conclusion and perspective

This review provides an overview of the fundamental processes and molecular mechanisms underlying heart development, along with insights into CHD and current treatment approaches. First, from a historical perspective, we review the progress of heart development research. Each significant breakthrough in cardiac development research has been accompanied by advancements in related disciplines and technologies. Early observational studies elucidated the origins and processes of heart development, while molecular biology techniques have enabled the study of molecular mechanisms across in vivo and in vitro models. Technological advances continue to provide us with more research tools, allowing for a multifaceted and multilevel understanding of heart development. Heart development is divided into five stages: pre-cardiac and cardiac mesoderm induction, cardiac crescent formation, heart tube formation, cardiac looping, and four-chambered fetal heart formation. Numerous molecular signals regulate each stage of heart development, including GFs, TFs, WNT signal, Shh signal, Hippo signal, Notch signal, ECM, and epigenetic modifications. These signals often play roles in multiple stages of heart development, sometimes exerting opposite effects. For instance, FGF and BMP signals are crucial from mesoderm cardiac induction to chamber formation. WNT signaling is essential for early mesoderm specification, and its subsequent inhibition is critical for myocardial cell differentiation, reflecting the complexity of the regulatory network involved in heart development. Additionally, additional molecular mechanisms, including metabolism, autophagy, and macrophages, have been shown to be involved in heart development regulation. Research into these mechanisms in heart development is still in its early stages, and their roles will undergo further investigation in the future.

Finally, we summarize diseases caused by abnormalities in heart development, including common CHDs such as septal defects, conotruncal defects, valve defects, PDA, HLHS, and various developmental diseases associated with a high incidence of CHD. Currently, CHD requires lifelong management, primarily through surgical and palliative care approaches. The limited treatment options available for CHD stem from our incomplete understanding of the mechanisms regulating heart development. As our understanding of cardiac development mechanisms deepens in the future, we can prevent the occurrence of most CHDs and diagnose and intervene early in those cases, thereby preventing abnormal cardiac development and restoring it to its normal trajectory. However, this goal remains distant for now, but the development of new technologies offers hope. Therefore, further understanding of the processes and mechanisms of heart development is essential for developing new and effective treatments for CHD.

With the advancement of new technologies, especially in recent years, with the development of stem cell and sequencing technologies, we are gradually shifting from a perspective focused on single molecules or signals to understanding heart development from the viewpoint of the entire regulatory network. This network encompasses not only the participation of many genes or proteins but also integrates changes across multiple levels, including DNA, RNA, proteins, epigenetic or posttranslational modifications, metabolism, and more, over time and space. The development of multi-omics technologies and advancements in AI facilitates this holistic approach to understand heart development. Stem cell technologies play a crucial supportive role in this process, providing new tools for studying heart development. hPSCs, derived from patients with genetic backgrounds associated with heart developmental defects, address the limitations of most heart development studies conducted on animal models. These cells can differentiate into various cardiac cell types, including cardiomyocytes,^271^ endothelial cells,^667^ epicardial cells,^668^ and smooth muscle cells,^669^ allowing the study of pathogenic mechanisms in different cardiac cells in vitro.

Furthermore, the application of novel technologies has expanded the potential utility of hPSC-derived CMs in heart development research. The integration of CRISPR/Cas9 technology with hPSCs enables the exploration of the effects of variants with unknown significance in cardiac development by introducing these variants into wild-type cell lines or correcting them in patient-specific cell lines.^670^ Additionally, unlike cardiac microtissues and engineered cardiac tissues, cardiac organoids are developed by applying tissue-specific differentiation factors and hydrogels to promote the self-organization of pluripotent stem cells into tissue patterns resembling the embryonic heart, exhibiting key features of cardiac development.^671^ Recent studies have demonstrated the creation of hPSC-derived complex, highly structured, three-dimensional heart-forming organoids, including myocardial layers surrounded by septum-transversum-like anlagen and endocardial-like cell layers.^672^ Another study utilized hPSCs to construct heart organoids with complex lumens and vascular systems capable of mimicking congenital heart defects induced by gestational diabetes.^673^ Another study generated cardioids from three different types of cardiac progenitors, including FHF progenitors differentiating into left ventricle-like cardioids, anterior SHF progenitors differentiating into right ventricle and OFT-like cardioids, and posterior SHF progenitors forming atrial and AVC cardioids. These parts were functionally connected to form a multi-chamber heart model with shared cavities. This study successfully used these cardioids to in vitro model cardiac defects, including the effects of *ISL1*-KO on the RV, atria, and OFT, and the impact of *TBX5*-KO on AVC and CM differentiation efficiency.^674^ With further development of stem cell and organoid technologies, constructing in vitro models of CHD through molecular signaling and gene editing may become feasible, offering a promising approach for exploring heart development mechanisms and drug screening.

Moreover, with the advancement of sequencing technologies, various sequencing techniques, including single-cell transcriptomics, spatial transcriptomics, metabolomics, and epigenomics, have been applied in the field of cardiac development, providing a comprehensive understanding of the mechanisms and processes of cardiac development. However, the accompanying large datasets make subsequent comprehensive analysis challenging, which can potentially be addressed by powerful AI technologies. The application of AI technologies such as machine learning or deep learning to process sequencing data related to cardiac development enables the integration of data from different sources, such as genomic data, epigenomic data, transcriptomic data, and clinical data. This approach helps identify gene variants and molecular features associated with CHD susceptibility, severity, and treatment response.^675,676^ Subsequent validation and research in in vitro and in vivo models will further advance our understanding of cardiac development processes and mechanisms, enabling early prevention and intervention for CHD. For instance, this approach could facilitate early screening of CHD in affected children by providing a more comprehensive CHD candidate genetic defect list or identifying risk factors and molecular signaling characteristics leading to CHD during pregnancy for early pharmacological treatment development, thereby avoiding the necessity for later surgical treatment. Nevertheless, this remains a distant goal requiring further research in the future.
